# Genome-wide identification and expression analysis of late embryogenesis abundant (*LEA*) genes reveal their potential roles in somatic embryogenesis in hybrid sweetgum (*Liquidambar styraciflua* × *Liquidambar formosana*)

**DOI:** 10.48130/FR-2023-0012

**Published:** 2023-05-29

**Authors:** Ying Li, Shuaizheng Qi, Siyuan Chen, Hongxuan Li, Ting Zhang, Fen Bao, Dingju Zhan, Zhenwu Pang, Jinfeng Zhang, Jian Zhao

**Affiliations:** 1 National Engineering Research Center of Tree Breeding and Ecological Restoration, Key Laboratory of Genetics and Breeding in Forest Trees and Ornamental Plants, Ministry of Education, The Tree and Ornamental Plant Breeding and Biotechnology Laboratory of National Forestry and Grassland Administration, College of Biological Sciences and Biotechnology, Beijing Forestry University, Beijing 100083, China; 2 Henan Province Key Laboratory of Germplasm Innovation and Utilization of Eco-Economic Woody Plant, Pingdingshan University, Pingdingshan, China; 3 Guangxi Bagui Forest and Flowers Seedlings Co., Ltd., Nanning, China

**Keywords:** Hybrid sweetgum, Late embryogenesis abundant (LEA) proteins, Somatic embryogenesis

## Abstract

Late embryogenesis abundant (LEA) proteins are widely distributed in higher plants that play significant roles in embryonic development and abiotic stress response. Hybrid sweetgum is an important forest tree resource around the world, and somatic embryogenesis is an efficient way of reproduction and utilization. However, a systematic analysis of the *LEA* family genes in hybrid sweetgum is lacking, this is not conducive to the efficiency of its somatic embryogenesis. From the whole genome of the hybrid sweetgum, utilizing hidden Markov models, an identification of a total of 79 *LEA* genes was successfully conducted. They were classified into eight different groups based on their conserved domains and phylogenetic relationships, with the *LsfLEA2* group of genes being the most abundant. The gene structure and sequence characteristics and chromosomal localization, as well as the physicochemical properties of LEA proteins were meticulously carried out. Analysis of the cis-acting elements shows that most of the *LsfLEA* genes are associated with light-responsive-elements. In addition, some genes are associated with biosynthetic pathways, such as abscisic acid response, growth hormone response, methyl jasmonate response, somatic embryogenesis, meristematic tissue expression. Furthermore, we systematically analyzed the expression patterns of hybrid sweetgum *LEA* genes in different stages of somatic embryogenesis and different tissues, in *LEA* family genes we also found significant specificity in gene expression during somatic embryogenesis. This study provides new insights into the formation of members of the *LsfLEA* family genes in hybrid sweetgum, while improving the understanding of the potential role of these genes in the process of hybrid sweetgum somatic embryogenesis and abiotic stress response. These results have a certain guiding significance for the future functional study of *LsfLEA* family genes, and provide a theoretical basis for exploring the regulatory mechanism of *LsfLEA* genes in the somatic embryo development stage of hybrid sweetgum.

## Introduction

LEA proteins were first identified in cotton seeds at the late embryonic development more than 30 years ago^[1]^. Following this discovery, these proteins have also been found in roots, stems, leaves, flowers and other tissues of plants. According to the similarity of amino acid sequences and conserved motifs, LEA proteins were divided into at least eight different groups (LEA1(PF03760), LEA2(PF03168), LEA3(PF03242), LEA4(PF02987), LEA5(PF00477), LEA6(PF10714), dehydrin (DHN) (PF00257) and seed maturation protein (SMP) (PF04927) in the Pfam database^[2]^. LEA proteins are widely distributed in higher plants^[3−5]^. To date, genome-wide identification and analysis of the *LEA* family genes have only been carried out in a few sequenced plant species genomes, such as *Arabidopsis thaliana*^[4]^, *Oryza sativa*^[6]^, maize^[7]^, *Solanum Tuberosum*^[8]^, cucumber^[9]^, *Solanum lycopersicum*^[10]^, *Brassica napus*^[6]^, cassava^[11]^, *Populus trichocarpa*^[12]^, *Prunus mume*^[13]^, *Pinus tabulaeformis*^[14]^ and *Canavalia rosea*^[15]^, linseed flax^[16]^, peanut^[17]^, *Dendrobium officinale*^[5]^, Brassica campestris^[18]^, Moso Bamboo^[19]^, watermelon and melon^[20]^, as well as in algae, fungi, bacteria^[21]^and even invertebrates^[22]^.

Intrinsically disordered in their natural form, a large proportion of LEA proteins are predominantly characterized by their remarkable hydrophilicity, owing to the substantial presence of charged amino acid residues. These residues, along with glycine or other small amino acids such as alanine, serine, and threonine, exhibit a remarkable lack or, in some instances, minimal amounts of cysteine and tryptophan^[23, 24]^. *LEA* genes are considered pivotal players in the intricate processes of plant growth and development, as they are thought to assume an important role in alleviating the adverse effects of various stress conditions on cells. A study has unveiled intriguing insights into the dynamic nature of these genes, revealing that they exhibit an intriguing pattern of accumulation during the final stages of seed maturation, which coincides with the onset of dehydration. Specifically, these genes were found to be present in high concentrations during this phase, suggesting a potential role in safeguarding the developing seed against environmental perturbations. The results of the study also shed light on the impressive adaptive capabilities of *LEA* genes, as they were shown to be significantly induced under abiotic stress conditions, such as cold, heat, and drought stress^[19, 25]^. Several scientific investigations have propounded a compelling postulate that the *LEA* genes may potentially be implicated in a defense mechanism against a plethora of deleterious abiotic stressors. These mechanisms include, but are not limited to, the fortification and maintenance of the structural integrity of the membrane^[26, 27]^, scavenging of free radicals^[28, 29]^, sequestering ions^[30]^ or biotin^[31]^.

Somatic embryogenesis, a process of profound significance in the arsenal of modern biotechnological methodologies, has been widely recognized as a highly efficient technique for generating complete embryos that can subsequently develop into fully-fledged plants. It is characterized by two distinct pathways, namely the somatic embryogenesis direct pathway and the somatic embryogenesis indirect pathway. These pathways offer an unparalleled degree of relative genetic stability, reproducibility, and remarkable efficiency, rendering them as one of the most prominent technical means of achieving successful plant regeneration, aside from the syncytial embryogenesis pathway^[32]^. The significance of somatic embryogenesis extends far beyond the mere confines of basic research on cell differentiation, gene expression, hormone signaling, and metabolic regulation^[33]^. In addition to their profound implications for these domains of scientific inquiry, somatic embryogenesis also furnish an efficacious avenue for the large-scale clonal propagation of forest trees, the protracted conservation of finely-tuned germplasm, and the consequential transformation of genes^[34]^.

*Liquidambar L.* species are literally multi-objective and multi-purpose tree species that possess a myriad of economic, ecological, and medicinal values. These trees are not only globally renowned for their outstanding forest resource status but also the main tree species in China's National Reserve Forest Construction Plan (2018−2035). *L. formosana* is recognized for its wide range of distribution, impressive adaptability to various ecological niches, and remarkable suitability as timber, *L. styraciflua* is characterized by a fast growth rate. With *L. styraciflua* as the female parent and *L. formosana* as the male parent, the hybrid sweetgum offspring demonstrates a salient and significant advantage over its parental counterparts, in fact, studies have confirmed that the growth velocity of this hybrid sweetgum exceeds that of *L. styraciflua*^[35]^. At present, hybrid sweetgum is mainly propagated and utilized by somatic embryogenesis, but its propagation efficiency needs to be further improved, the mechanism of somatic embryogenesis is still unclear, and there is a lack of research on the mining of key regulatory genes and their functions.

In this study, the genome-wide identification of the *LsfLEA* family genes were performed on the hybrid sweetgum genome and transcriptome using the Hidden Markov Model (HMM), and its sequence features, phylogenetic relationships, conserved motifs and gene structure were investigated. Meanwhile, the expression profiles of hybrid sweetgum *LEA* genes were analyzed in a series of developmental processes in non-embryogenic callus (NEC), embryogenic callus (EC), globular embryos (GE), heart-shaped embryos (HE), torpedo-shaped embryos (TE), cotyledonary embryos (CE), roots (R), stems (S), and leaves (L).This study provides new insights into the *LEA* family genes of hybrid sweetgum, which will contribute to the study of the function of genes related to somatic embryogenesis of hybrid sweetgum and the interpretation of the mechanism.

## Materials and methods

### Plant materials and growing conditions

The SF15SH-5a embryonic cell line was induced from hybrid seeds obtained from *L. formosana* and *L.* *styraciflua* trees, as described previously^[36]^. The non-embryogenic callus (NEC) cells and embryogenic callus (EC) cells were previously reserved in our laboratory. To analyze the tissue- specific expression profile of the identified *LsfLEA* genes, each 90-mm plastic petri plate was inoculated with 10 clumps of EC on fresh induction medium of the same composition in three replicates and callus were transferred to fresh medium every 3−4 weeks, the medium was transferred to fresh media of the same composition monthly. The samples were incubated in an incubator at a constant temperature of (25 ± 2) °C and cultured in the dark. The EC obtained after induction for a period of time was transferred into the maturation medium in the same way as described above, embryos with different morphologies were collected as samples. At the stage of cotyledon embryo, they were moved to the germination medium in the tissue culture room of Beijing Forestry University (China), and cultured into plants. The roots and leaves were collected as materials for subsequent experiments. Composition of the medium for each stage are given in Table 1. All samples were immediately frozen in liquid nitrogen and stored at -80°C for subsequent gene expression analysis. Three independent biological replicate experiments were performed.

**Table 1:** Medium used in the experiment.

Medium	Composition
Induction/ proliferation	Basic medium^[37]^ + 1 mg 2, 4-D + 0.5 mg 6-BA + 40 g/L sucrose + 1 g/L enzymatic hydrolyzed casein 2.6−2.7 g/L vegetable gel, pH adjusted to 5.6~5.7
Mature	Basic medium + 40 g/L sucrose + 6 g/L vegetable gel +5% PEG, pH was adjusted to 5.6~5.7
Germination	Basic medium + 40 g/L sucrose + 2.6-2.7 g /L vegetable gel, pH was adjusted to 5.6~5.7

### Identification and characterization of *LEA* genes in hybrid sweetgum

The HMM model's advantages over BLAST are significant. Its ability to handle probabilistic models with hidden states, flexibility in modeling different types of sequences, and accurate modeling of sequence variation within related sequences make it an invaluable tool for studying biological sequences. The hidden Markov models of *LEA* gene subfamilies (PF03760 (LEA1), PF03168 (LEA2), PF03242 (LEA3), PF02987 (LEA4) and PF00477) were downloaded from the Pfam database (http://pfam.xfam.org/) (LEA5), PF10714 (LEA6), PF00257 (DHN) and PF04927 (SMP). Tbtools^[38]^ was used to perform HMM search on the hybrid sweetgum genome database (our laboratory has successfully completed the whole genome sequencing of hybrid sweetgum, and we are eagerly anticipating the imminent publication of this invaluable data) to obtain all putative *LEA* genes of hybrid sweetgum. All identified candidate genes were analyzed by the Pfam database (http://pfam.xfam.org/)^[2]^ and the NCBI conserved domain search database (www.ncbi.nlm.nih.gov/Structure/cdd/wrpsb.cgi)^[39]^ to verify and ascertain the presence of conserved domains within the *LEA* genes. The *LsfLEA* genes that lacked a conserved domain and complete CDS sequence were removed. Additionally, any redundant *LsfLEA* genes were also deemed superfluous and therefore, justifiably excluded.

To thoroughly investigate the complex and multifaceted properties of the *LsfLEA* genes, a comprehensive array of analytical techniques were employed. To begin with, the molecular weight (MW), theoretical pI, instability index, aliphatic index and grand average of hydropathicity (GRAVY) were scrupulously predicted utilizing the powerful and sophisticated ProtParam tool (http://web.expasy.org/)^[40]^. In addition, with an eye towards elucidating the subcellular localization of the LsfLEA proteins, we leveraged the remarkable predictive capabilities of the PSORT Prediction tool (www.genscript.com/wolf-psort.html)^[41]^. We conducted a rigorous and exhaustive analysis of their cis-acting elements, leveraging the PlantCARE (http://bioinformatics.psb.ugent.be/webtools/plantcare/html) online website^[42]^. Moreover, protein hydrophobicity analysis utilizing ExPASy ProtScale (https://web.expasy.org/protscale), we conducted a detailed analysis of their signal peptides, leveraging the powerful and intuitive online website Signa1P Server (https://services.healthtech.dtu.dk/service.php?SignalP-5.0). Phylogenetic analysis of 79 *LsfLEA* and 51 *AtLEA*^[4]^ amino acid sequences was performed using the 1,000 bootstrapped Maximum Likelihood(ML) method^[43]^ of MEGA 7.0 software. Chromosome localization and visualization were done using TBtools^[38]^ software.

### Structural and protein conserved sequence analysis of *LsfLEA* genes

Structural analysis of the identified *LsfLEA* genes in the hybrid sweetgum genome was performed using Tbtools^[38]^ software. The conserved structural domains of the *LsfLEA* family genes were analyzed using the MEME (Multiple Expectation Maximization for motif Elicitation) Suite (http://meme-suite.org/)^[44]^. Different subfamilies of *LEA* genes were labeled with different colors. Fourty conserved structural domains (Motif 1-Motif 40) were identified in the *LsfLEA* subfamily genes and labeled with different colors and numbers.

### Chromosome location and gene duplication of the *LsfLEA* genes

DNA sequences from the *LEA* family genes were mapped onto the genome of hybrid sweetgum. Distribution of the genes on the chromosomes or scaffolds was calculated by the TBtools^[38]^ software. Duplicate events of the *PtrLEA* genes were calculated and visualized for mapping by using the TBtools^[38]^ software.

### Analysis of cis-acting elements of *LsfLEA* genes

To explore cis-elements in the gene promoter of *LsfLEA*, a 2 kb genomic sequence upstream of the transcription start site (TSS) of each gene was analyzed, the PlantCare database was extracted and searched to identify and count cis-elements associated with abiotic stress responses.

### Heatmap of the differentially expressed 79 *LsfLEA* genes during hybrid sweetgum SE and different tissues

Combined with transcriptome data^[36]^ and whole genome data, TBtools^[38]^ software was used to draw the heatmap of gene expression in different periods and different tissues of hybrid sweetgum. Heatmap indicate the gene expression level by Log2[FPKM] with a color scale, each row represents a single gene, the IDs are indicated to the outside of fan, and each circle represents a sample.

### Visualization of *LEA* key genes in different stages of somatic embryogenesis by qRT-PCR

These six genes were selected from the hybrid sweetgum *LEA* genes, specifically *LsfLEA1-1* (EVM0027957), *LsfLEA3-3* (EVM0021267), *LsfLEA6-2* (EVM0005932), *LsfDHN-2* (EVM0003818), *LsfLEA1-3* (EVM0000925), *LsfSMP-1* (EVM0023588). To validate the efficacy of these genes, we employed qRT-PCR gene expression analysis, and compared the gene expression levels with gene FPKM values obtained from transcriptomic data^[36]^. Using the plant RNA extraction kit, we extracted total RNA in accordance with the kit's instructions. In order to assess the concentration and quality of RNA samples, we utilized a NanoDrop 2,000 spectrophotometer (Thermo Scientific, Wilmington, DE, USA) and 1% agarose gel electrophoresis. The extracted total RNA was then subjected to reverse transcription using the TransScript® First Strand Synthesis Kit. For the purpose of qRT-PCR analysis, we designed specific primers utilizing the Primer Premier 5.0. The detailed primer sequences can be found in Table 2. The qRT-PCR analysis was conducted with the QuantStudio 6 Flex real-time PCR system. Each PCR reaction mixture consisted of 10 μL 2×PerfectStart ®Green qPCR SuperMix(Yisheng Biotechnology Shanghai Co., Ltd.), 1 μL cDNA, 0.4 μL forward primer, 0.4 μL reverse primer, and 8.2 μL ddH_2_O to achieve a final volume of 20 μL. The amplification conditions for qRT-PCR were as follows: 45 cycles of 94 °C for 30 s, 94 °C for 5 s, and 60 °C for 30 s. Apple *EF1-α* gene^[45]^ was utilized as an internal reference control. The relative gene expression was calculated using the 2^−ΔΔCᴛ^ method, with three biological replicates and three technical replicates being carried out.

**Table 2:** Primer sequence information of qRT-PCR.

Gene_ name	Gene_id	Primer sequence
*LsfLEA1-1*	EVM0027957	F: TATTGGGGTGATGACGGTCC R: TTCCTCTGCGTGGCCATATC
*LsfLEA3-3*	EVM0021267	F: ATACTCGGCGGCATCACAAG R: ACGTCAATCTCTTCCGCACG
*LsfLEA6-2*	EVM0005932	F: TGGAGGACTACAAGCGTCAAG R: CACCACCGGAAAGAGTGGG
*LsfDHN-2*	EVM0003818	F: CCAATTGGGTTGGAAACCGTC R: TCACCGAGCTAGAGCTTGAAC
*LsfLEA1-3*	EVM0000925	F: AGGAGAAGTTGAGCGACATGG R: TTTGCTTGAGCTTCCTTCGC
*LsfSMP-1*	EVM0023588	F: ATCGTGATCTGACCGGCATC R: CATCAACCACCACCACATGC
*EF1-r-07041*	/	F: ACTGCACGGTCATTGATGCT R: AAAGCATGCTCACGGGTCTG

### Plasmid construction and subcellular localization analysis

Based on the *LsfLEA1-3* CDS, cloning primers *LsfLEA1-3-F*: ATGCATTCTGCAAAGGAGAAGT; *LsfLEA1-3-R*: CTACATATACTCACGTCGAGGAGG were designed by Primer 5. The cDNA from embryogenic callus of hybrid sweetgum was used as a template to amplify the *LsfLEA1-3* CDS. The 50 µL PCR system included: 2 ×Phanta®Max Master Mix 25 µL, cDNA 2 µL, each primer 2.5 µL, ddH_2_O 18 µL, and the PCR program as follows: pre-denaturation at 95 °C for 3 min; denaturation at 95 °C for 15 s, annealing at 56 °C for 15 s, extension at 72 °C for 1 min, 34 cycles; extension at 72 °C for 5 min. Then that product was ligated into the pEasy-Blunt3 vector (TransGen Biotech) and sequenced. They were transformed into *E. coli* DH5α, cultured overnight for 12 h, and the monoclonal clones were selected for PCR identification, and the bacterial solution with the correct band was sequenced by using 1300-F：GACGCACAATCCCACTATCC；GFPfusion-R: CTCCACTGACAGAAAATTTG as primers. Primer sequences at both ends respectively introduced restriction enzymes Kpn1 and the corresponding protection bases, primer sequences for *LsfLEA1-3-1300-*F: 5'-CGGGGGACGAGCTCGGTACCATGCATTCTGCAAAGGAGAAGTTGA-3' and *LsfLEA1-3-1300-*R: 5'-CTAGAGGATCCCCGGGTACCCATATACTCACGTCGAGGAGGGTG-3', and the carrier of linearization, adopt the method of homologous recombination connect it. The *LsfLEA1-3* was amplified and ligated into an expression pCAMBIA1300 vector with a GFP fluorescent label and a CaMV35 S promoter. Then, the successfully constructed plasmid was transferred to Agrobacterium GV3101 through the conventional freezing-thawing method. Next, empty pCAMBIA1300-GFP or pCAMBIA1300- *LsfLEA1-3*-GFP GV3101 instantly infiltrated the strain into 5-week-old N. benthamiana leaves with expression buffer (10 mM MES pH 5.6, 10 mM MgCl_2_, 200 µM acetosyringone). After the infiltrated *N. benthamiana* was cultured in the dark for 12 h and then in low light for 24 h, the fluorescence signals were captured with a confocal laser‐scanning microscope (TCS SP8; Leica).

## Results

### Identification and characteristic analysis of *LEA* gene in hybrid sweetgum

**Figure 1:**
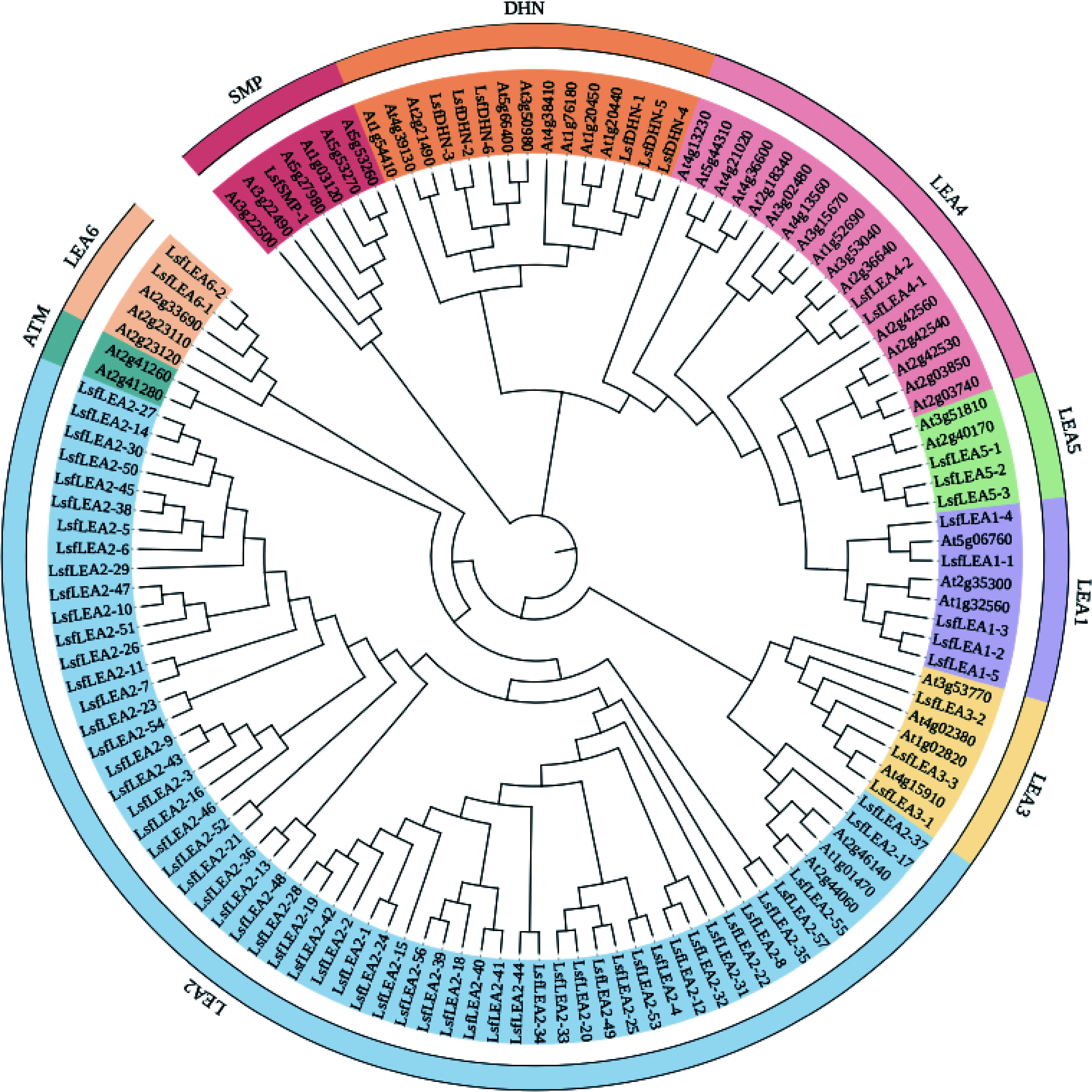
Maximum likelihood phylogenetic tree of 79 *LsfLEA* genes and 51 *AtLEA* genes. The phylogenetic tree was constructed using MEGA 7 software with 1000 bootstrap replicates. We used the online site iTOL for evolutionary tree beautification, and the 9 major groups are marked with different colored backgrounds.

Based on the HMM search of LEA1 (PF03760), LEA2 (PF03168), LEA3 (PF03242), LEA4 (PF02987), LEA5 (PF00477), LEA6 (PF10714), SMP (PF04927) and DHN (PF00257), a total of 79 *LEA* genes were identified from the whole genome of hybrid sweetgum, which were meticulously documented in Table 3. Upon close analysis, it became evident that the *LsfLEA* genes were divided into 8 subfamilies, each unique in their conserved domain, as displayed in Fig. 1. The *LEA2* subfamily genes were notably the most significant, consisting of an astonishing 57 members, while the numbers of *LEA1*, *LEA3*, *LEA4*, *LEA5*, *LEA6*, *DHN*, and *SMP* subfamilies were more modest, with 5, 3, 2, 3, 2, 6, and 1 member, respectively. Upon further investigation, it was discovered that the predicted 79 *LsfLEA* genes encoded peptides with an extensive range of amino acid lengths, spanning from a diminutive 89 (*LsfLEA6-1*, *LsfLEA6-2*) to an impressive 323 (*LsfLEA4-2*) amino acids. Additionally, the molecular weight ranged from a minute 9.37028 (*LsfLEA6-2*) to 36.9282 (*LsfLEA4-2*) kDa. Further analysis of the calculated grand average of hydropathicity indexes revealed a total mean ranging from a minuscule −1.533 to a noteworthy 0.407. Furthermore, the hydrophilicity values of 65 *LsfLEA* genes (82%) were lower than 0, indicating the hydrophilic nature of most *LsfLEA* genes. The prediction of subcellular localization indicated a diverse and intricate distribution of *LsfLEA* genes, with *LsfLEA1* genes found in both the nucleus and cytoplasm. Most *LsfLEA2* genes were placed in chloroplasts, nucleus, cell walls, and membranes, while all *LsfLEA3* genes were presented in chloroplasts. All *LsfLEA4*, *LsfLEA5*, and *LsfLEA6* genes were found in the nucleus, while *LsfDHN* genes were contained in both the cytoplasm and nucleus. These intricate and diverse details are documented in Table 3.

**Table 3:** The characteristics of *LsfLEA* genes.

*Name*	Gene ID	Family	Pfam ID	Length	kD	pI	GRAVY	Subcellular localization
*LsfLEA1-1*	EVM0027957	LEA1	PF03760	191	19.36702	6.8	−0.865	Cytoplasm. Nucleus.
*LsfLEA1-2*	EVM0018353	LEA1	PF03760	120	13.7979	9.25	−0.863	Nucleus.
*LsfLEA1-3*	EVM0000925	LEA1	PF03760	169	18.18858	8.77	−0.731	Nucleus.
*LsfLEA1-4*	EVM0022193	LEA1	PF03760	164	16.51795	8.83	−0.946	Cytoplasm. Nucleus.
*LsfLEA1-5*	EVM0016558	LEA1	PF03760	107	12.32725	9.33	−0.769	Nucleus.
*LsfLEA2-1*	EVM0002770	LEA2	PF03168	239	26.42419	10	−0.094	Cell membrane. Cell wall.
*LsfLEA2-2*	EVM0027034	LEA2	PF03168	205	22.97259	9.84	−0.017	Chloroplast.
*LsfLEA2-3*	EVM0005964	LEA2	PF03168	177	19.27253	10.27	0.042	Chloroplast.
*LsfLEA2-4*	EVM0015809	LEA2	PF03168	190	21.8101	10.03	−0.482	Chloroplast.
*LsfLEA2-5*	EVM0019694	LEA2	PF03168	214	23.44119	9.91	−0.121	Cell membrane. Nucleus.
*LsfLEA2-6*	EVM0004956	LEA2	PF03168	210	23.12978	9.97	−0.186	Chloroplast.
*LsfLEA2-7*	EVM0027836	LEA2	PF03168	219	24.18402	9.73	−0.195	Chloroplast.
*LsfLEA2-8*	EVM0001803	LEA2	PF03168	237	27.18655	9.15	−0.103	Chloroplast.
*LsfLEA2-9*	EVM0020637	LEA2	PF03168	178	19.33671	10.25	0.086	Chloroplast.
*LsfLEA2-10*	EVM0015055	LEA2	PF03168	184	19.86985	9.92	−0.223	Chloroplast. Nucleus.
*LsfLEA2-11*	EVM0017871	LEA2	PF03168	212	23.53223	10	−0.157	Chloroplast. Nucleus.
*LsfLEA2-12*	EVM0018801	LEA2	PF03168	260	28.41312	9.01	0.133	Chloroplast.
*LsfLEA2-13*	EVM0013310	LEA2	PF03168	198	21.34478	9.37	0.407	Cell membrane.
*LsfLEA2-14*	EVM0010683	LEA2	PF03168	215	23.44427	9.88	−0.083	Cell membrane. Nucleus.
*LsfLEA2-15*	EVM0023593	LEA2	PF03168	201	22.72988	10.05	0.109	Chloroplast. Nucleus.
*LsfLEA2-16*	EVM0028234	LEA2	PF03168	188	20.34671	10.27	−0.001	Chloroplast.
*LsfLEA2-17*	EVM0003550	LEA2	PF03168	151	16.49626	5.58	0.077	Chloroplast. Nucleus
*LsfLEA2-18*	EVM0008005	LEA2	PF03168	265	29.16785	9.35	−0.066	Chloroplast.
*LsfLEA2-19*	EVM0007435	LEA2	PF03168	267	29.08778	8.38	−0.215	Cell membrane. Cell wall. Nucleus.
*LsfLEA2-20*	EVM0028118	LEA2	PF03168	223	25.25915	10.05	−0.122	Chloroplast. Mitochondrion.
*LsfLEA2-21*	EVM0001898	LEA2	PF03168	215	23.68493	10.14	−0.021	Nucleus.
*LsfLEA2-22*	EVM0018273	LEA2	PF03168	232	24.94073	9.3	0.113	Cell membrane. Chloroplast.
*LsfLEA2-23*	EVM0025800	LEA2	PF03168	189	20.97945	10.01	−0.074	Chloroplast. Nucleus. Peroxisome.
*LsfLEA2-24*	EVM0013583	LEA2	PF03168	264	29.43273	8.93	−0.048	Cell wall. Nucleus.
*LsfLEA2-25*	EVM0020355	LEA2	PF03168	306	34.40006	9.47	−0.383	Cell membrane. Chloroplast.
*LsfLEA2-26*	EVM0011840	LEA2	PF03168	214	23.38122	10.14	−0.056	Chloroplast.
*LsfLEA2-27*	EVM0023465	LEA2	PF03168	215	23.46828	9.91	−0.064	Cell membrane. Nucleus.
*LsfLEA2-28*	EVM0021483	LEA2	PF03168	262	29.00554	6.51	−0.288	Cell wall.
*LsfLEA2-29*	EVM0024390	LEA2	PF03168	213	23.36631	9.94	−0.035	Nucleus.
*LsfLEA2-30*	EVM0024154	LEA2	PF03168	215	23.32817	9.88	−0.031	Chloroplast.
*LsfLEA2-31*	EVM0007128	LEA2	PF03168	244	27.2663	10.2	−0.063	Chloroplast.
*LsfLEA2-32*	EVM0007725	LEA2	PF03168	259	28.26897	9.17	0.09	Chloroplast.
*LsfLEA2-33*	EVM0019270	LEA2	PF03168	223	24.39271	9.61	0.041	Cell wall. Chloroplast.
*LsfLEA2-34*	EVM0011791	LEA2	PF03168	210	23.90993	9.78	0.129	Cell membrane. Chloroplast.
*LsfLEA2-35*	EVM0015677	LEA2	PF03168	191	20.8606	9.28	0.33	Chloroplast.
*LsfLEA2-36*	EVM0011399	LEA2	PF03168	209	22.83086	9.98	0.197	Chloroplast.
*LsfLEA2-37*	EVM0020583	LEA2	PF03168	151	16.45712	5.04	0.054	Cell membrane. Nucleus.
*LsfLEA2-38*	EVM0011512	LEA2	PF03168	213	23.27806	9.97	−0.17	Chloroplast. Nucleus
*LsfLEA2-39*	EVM0000290	LEA2	PF03168	251	28.02687	9.56	−0.076	Chloroplast.
*LsfLEA2-40*	EVM0006920	LEA2	PF03168	269	29.83144	10.03	−0.246	Nucleus.
*LsfLEA2-41*	EVM0027367	LEA2	PF03168	253	27.51712	10.3	−0.089	Cell membrane. Cell wall.
*LsfLEA2-42*	EVM0005315	LEA2	PF03168	311	34.5181	9.07	−0.186	Cell wall. Nucleus.
*LsfLEA2-43*	EVM0025389	LEA2	PF03168	178	19.27267	10.25	0.108	Chloroplast.
*LsfLEA2-44*	EVM0000222	LEA2	PF03168	186	21.21822	7.87	−0.163	Cell membrane. Chloroplast.
*LsfLEA2-45*	EVM0018178	LEA2	PF03168	212	23.1178	9.91	−0.134	Cell membrane. Cell wall.
*LsfLEA2-46*	EVM0005757	LEA2	PF03168	178	19.26954	10.06	0.087	Chloroplast.
*LsfLEA2-47*	EVM0016553	LEA2	PF03168	209	22.80349	10.14	−0.098	Chloroplast.
*LsfLEA2-48*	EVM0016432	LEA2	PF03168	210	23.00723	9.83	0.226	Mitochondrion. Nucleus.
*LsfLEA2-49*	EVM0021824	LEA2	PF03168	308	34.60045	9.64	−0.337	Cell membrane. Chloroplast.
*LsfLEA2-50*	EVM0023803	LEA2	PF03168	214	23.29603	9.88	−0.102	Cell membrane. Cell wall.
*LsfLEA2-51*	EVM0017004	LEA2	PF03168	197	21.68226	10.22	−0.131	Chloroplast.
*LsfLEA2-52*	EVM0012352	LEA2	PF03168	217	24.03337	10.21	0.105	Chloroplast.
*LsfLEA2-53*	EVM0023248	LEA2	PF03168	225	24.9939	9.55	−0.009	Cell membrane. Chloroplast.
*LsfLEA2-54*	EVM0006284	LEA2	PF03168	213	23.40402	9.56	−0.175	Chloroplast. Cytoplasm. Nucleus.
*LsfLEA2-55*	EVM0008344	LEA2	PF03168	315	34.64837	4.75	−0.403	Nucleus.
*LsfLEA2-56*	EVM0022790	LEA2	PF03168	253	28.07601	10.17	−0.1	Cell membrane. Cell wall.
*LsfLEA2-57*	EVM0012837	LEA2	PF03168	191	21.15586	9.58	0.26	Chloroplast.
*LsfLEA3-1*	EVM0028438	LEA3	PF03242	101	10.79223	9.88	−0.222	Chloroplast. Nucleus.
*LsfLEA3-2*	EVM0019119	LEA3	PF03242	94	10.47005	9.37	−0.418	Chloroplast.
*LsfLEA3-3*	EVM0021267	LEA3	PF03242	121	13.48961	10.28	−0.668	Chloroplast.
*LsfLEA4-1*	EVM0019164	LEA4	PF02987	212	23.759	5.42	−1.533	Nucleus.
*LsfLEA4-2*	EVM0021387	LEA4	PF02987	323	36.9282	9.53	−1.53	Nucleus.
*LsfLEA5-1*	EVM0020631	LEA5	PF00477	94	9.93673	5.48	−1.26	Nucleus.
*LsfLEA5-2*	EVM0022920	LEA5	PF00477	114	12.14812	5.53	−1.278	Nucleus.
*LsfLEA5-3*	EVM0002005	LEA5	PF00477	114	12.07605	5.91	−1.251	Nucleus.
*LsfLEA6-1*	EVM0004641	LEA6	PF10714	89	9.40021	4.75	−0.939	Nucleus.
*LsfLEA6-2*	EVM0005932	LEA6	PF10714	89	9.37028	5.77	−1	Nucleus.
*LsfSMP-1*	EVM0023588	SMP	PF04927	259	26.66671	4.66	−0.253	Nucleus.
*LsfDHN-1*	EVM0028566	Dehydrin	PF00257	290	32.91761	5.23	−1.388	Nucleus.
*LsfDHN-2*	EVM0003818	Dehydrin	PF00257	174	18.52736	8.01	−1.126	Cytoplasm.
*LsfDHN-3*	EVM0011412	Dehydrin	PF00257	174	18.62131	9.45	−1.31	Cytoplasm.
*LsfDHN-4*	EVM0018312	Dehydrin	PF00257	193	21.77947	5.56	−1.388	Nucleus.
*LsfDHN-5*	EVM0008852	Dehydrin	PF00257	1891	21.36301	5.46	−1.348	Nucleus.
*LsfDHN-6*	EVM0003289	Dehydrin	PF00257	155	16.54385	9.1	−1.369	Cytoplasm.

### Analysis of *LEA* gene structure and protein conserved sequence of hybrid sweetgum

**Figure 2:**
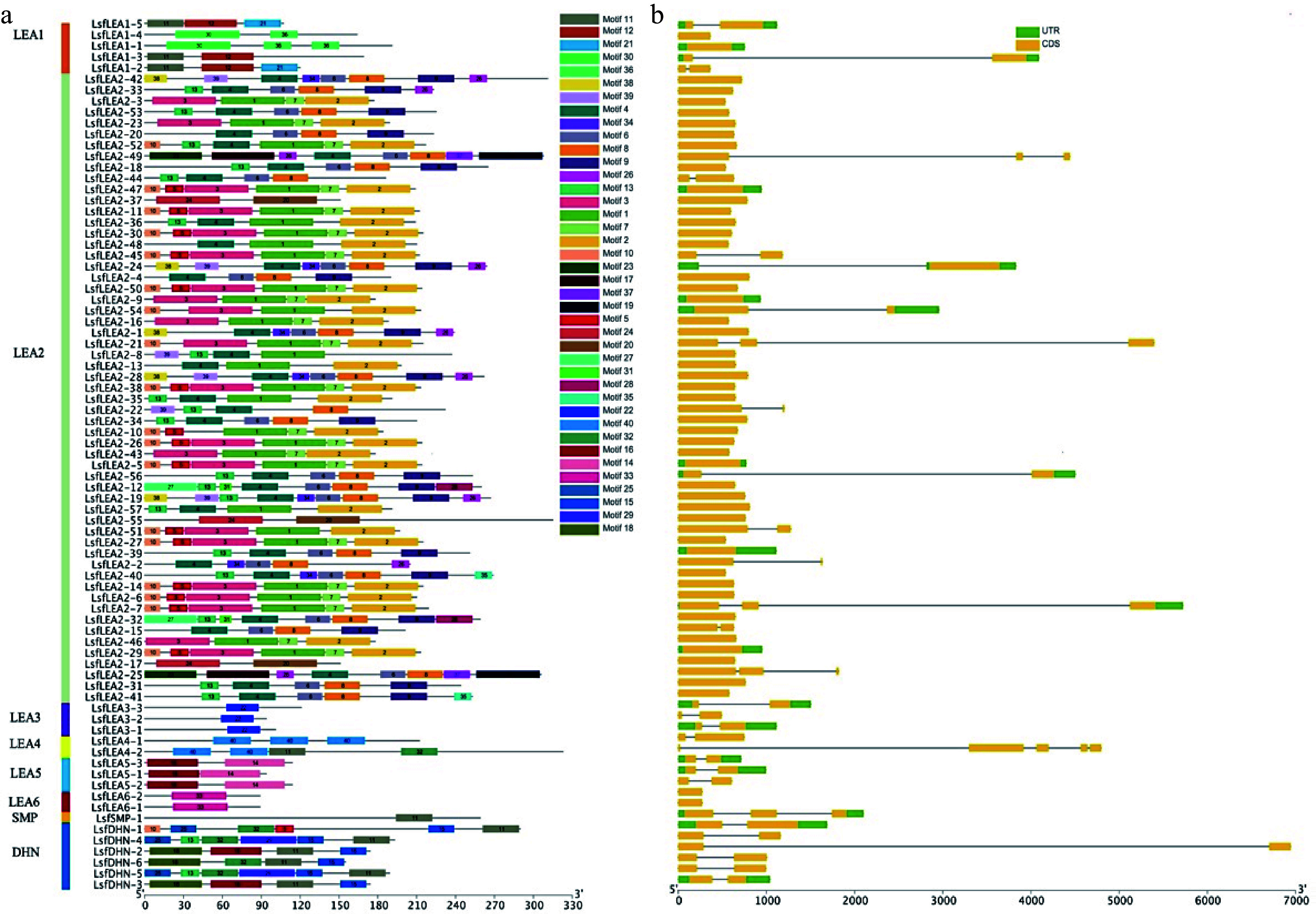
(a) Motif analysis of *LsfLEA* genes from hybrid sweetgum. The conserved motifs of each group on the right side were identified by the MEME web server. Different motifs are represented by different colored boxes (b) Gene structure distribution map of *LsfLEA*.

The number of introns in the *LsfLEA* gene is small, with the majority exhibiting a mere one or two introns. Upon scrutinizing conserved motifs, it was uncovered that the vast majority of homologous members within the same group shared indistinguishable conserved motifs (Fig. 2a). When undertaking an exhaustive analysis of gene structure, it was noticed that there existed a degree of differentiation in the gene structure of *LsfLEA* genes, with the exception of those in the same subfamily that possessed virtually identical structures. An exemplar of this was seen with *LsfLEA2-9*, *LsfLEA2-45*, *LsfLEA2-54*, *LsfLEA2-26*, *LsfLEA2-5* and *LsfLEA2-51*, which all exhibited similar gene structures (Fig. 2b). The members of the same group are presumed to have relatively similar biological functions. These results suggest that the composition of conserved structural domain motifs differs among different *LsfLEA* subfamilies, but the composition of conserved structural domain motifs of *LsfLEA* genes is extremely similar within the same subfamily, and the motifs of these structural domains are also conserved. Motif sequences and amino acid sequences of 79 *LEA* genes in hybrid sweetgum are given in Table 4 & Supplemental Table S1.

**Table 4:** The motif sequences of *LsfLEA* genes.

Motif number	Motif sequences
1	NTRFVAQVTVKNTNFGPYKFDNTTATFTYGGMTVGZVVIPKGKAGARSTK
2	SDISSGILKLSSQAKLSGKVELMFVMKKKKSAEMNCTLTFBLSAKALQAL
3	RRIKIAAYIVAFVVFQSIIIAIFAVTVMKYRTPKFRLGKVTVETLTSTPA
4	VVALJLWLVFRPKRPKFTVNSVSVSSFNL
5	DEESATLQSKEFRRK
6	RNPNKKJSIYYDSIEASVYY
7	KIBVTVDLNSDALTS
8	QQILSSGSLPPFYQGRKNTTVLSTTLAG
9	FBVKVDARVRFKVGRWKTKRVGVRVSCDG
10	MAEKNQQVYPAAP
11	SAAEKKKGMKEKIKEKLPGYKAKAEEEKE
12	HEGRKAKEAQAKMEMHEAKARHAEEKLNAKQSHLYGHQVHE
13	PKRERRRGCCCCGCL
14	KGGQTRKEQLGTEGYQEMGRKGGLSTTDQSGGERAAELGVDIDESKF
15	HHPEEKKGILEKIKEKLPGQH
16	GNREQREELEARARQGETVVPGGTGGGSLEAQEHLAEGRS
17	PHHYHCSPIHHSRESSTSRFSASLKNAHHHKHSAPWKKMHRVVDVDDDD
18	RDEYGNPIQLTDERGNPVQLTDEYGNPMHLSGVATTHGTTA
19	LNLTFVMRSRAYILGKLVKSKFYKRIRCSVTLRGNQLGKLQNLTDSCIYQ
20	NDITMLDVGLKVPHNVLVTLVKDIGADWDIDYELEVGLTIDLPIIGNFTI
21	HIIGTMNQWWVSMDTMGRSLLELRPLYLY
22	WVPDPVTGYYRPENRAGEIDVAELRD
23	MHAKSDSEVTSLDASSPPRSPRRPLYYVQSPSQHDVEKMSY
24	NFMVEKVANIKKPEASVEDVDLKDVSRECIDYNAKVAVKNPYGHALPICE
25	MADQHFQCHEKEPRDRGMFDF
26	GTMVGGPRECKVRL
27	MADSAIRKTEDSPPSSKPSPNPTSKPVRHVVFSEIPCRPHK
28	IQQSQVDIGQEPKCSVKMFSFRLHTFLFI
29	EGGEEKKKKKGLKEKIKEKMACQGEEEVTEIPVDKCDNIVDAET
30	GMDKTKATMQZKVEKMTARDPIZKEMATZKKEAKKTEAELNKQEAREHNA
31	CCAWGCMIGF
32	EPQEKKEVEKPTLVEELRRSGSSSSSSSD
33	GLPLESSPYVKNSDLEDYKRKGYGTEGHLDPKPGRGAGGTDAP
34	SSQITGNWNVSFYV
35	CKVDLRIKIWKWTF
36	GHPTGGHQMSAMPGEGTWQPTW
37	YQVPLYGGVSVLGGARNHYEN
38	MEDQKKPVIGYPVQFYHP
39	NTAAAHVVQPPPVVHHQQQ
40	GKVGEYKDYAAEKAKETKDSALEKAREYKD

### Chromosomal localization of the hybrid sweetgum *LEA* genes

**Figure 3:**
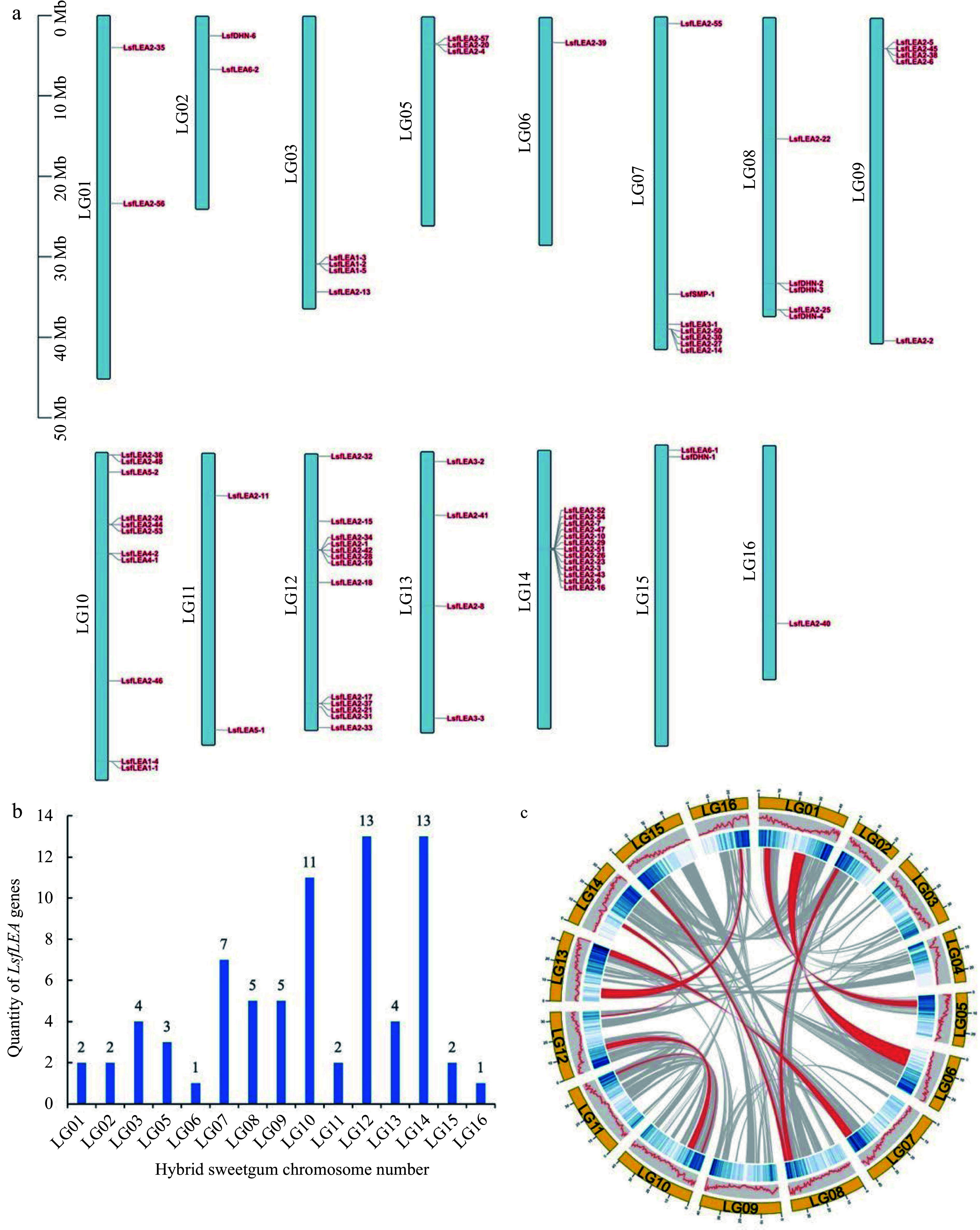
Chromosomal localization of the *LsfLEA* genes and gene duplication events. (a) Chromosomal localization of hybrid sweetgum *LsfLEA* gene. (b) Numbers of *LsfLEA* genes on each chromosome in hybrid sweetgum. (c) Duplicate pairs of the *LsfLEA* genes in hybrid sweetgum. The red lines represent collinear pairs of the *LsfLEA* genes.

The chromosomal locations of 79 hybrid sweetgum *LEA* genes were queried in the hybrid sweetgum genome database, and the chromosomal localization of the *LsfLEA* genes and gene duplication events were located by TBtools software. As the data poured in, we were able to pinpoint the precise chromosomal location of a staggering 75 genes, leaving only four genes (*LsfLEA2-12*, *LsfLEA5-3*, *LsfLEA2-49*, and *LsfDHN-5*) dispersed across unassembled scaffolds (Fig. 3a). The distribution of the *LsfLEA* genes across different chromosomes was marked by a noteworthy level of diversity and heterogeneity (Fig. 3b). It has been observed that the chromosomes LG12 and LG14 are equipped with the highest number of *LsfLEA* genes, 13 *LsfLEA* genes each. It is worthy of note that LG10 has 11 *LsfLEA* genes distributed across its length. In contrast, the chromosomes LG06 and LG16 have been found to be wanting when it comes to the number of *LsfLEA* genes present on them, boasting only one *LsfLEA* gene each. What's truly intriguing is that the *LsfLEA2* subfamily genes have been identified on as many as seven chromosomes. These chromosomes are LG01, LG05, LG06, LG09, LG12, LG14, and LG16. On the other hand, the *LsfLEA1* subfamily genes have been found to be distributed across only two chromosomes, LG03 and LG10. The *LsfLEA3* subfamily genes are confined to only two chromosomes as well, LG07 and LG13. Moreover, it is fascinating to observe that the *LsfLEA4* subfamily genes are present only on the LG10 chromosome. Further analysis has revealed that the *LsfLEA5* subfamily genes have been distributed across the LG10 and LG11 chromosomes, while the *LsfSMP* family genes have been identified solely on the LG07 chromosome. The *LsfDHN* family genes, meanwhile, are distributed across the LG02, LG08, and LG15 chromosomes. In addition, we found that the highest number of subfamilies were located on chromosome LG10, including four subfamilies of *LEA1*, *LEA2*, *LEA4* and *LEA5* genes. The evaluation of collinearity within the hybrid sweetgum species revealed an interesting discovery, characterized by a grand total of nine gene duplication events. (Fig. 3c).

### Analysis of cis-acting elements of *LEA* genes in hybrid sweetgum

**Figure 4:**
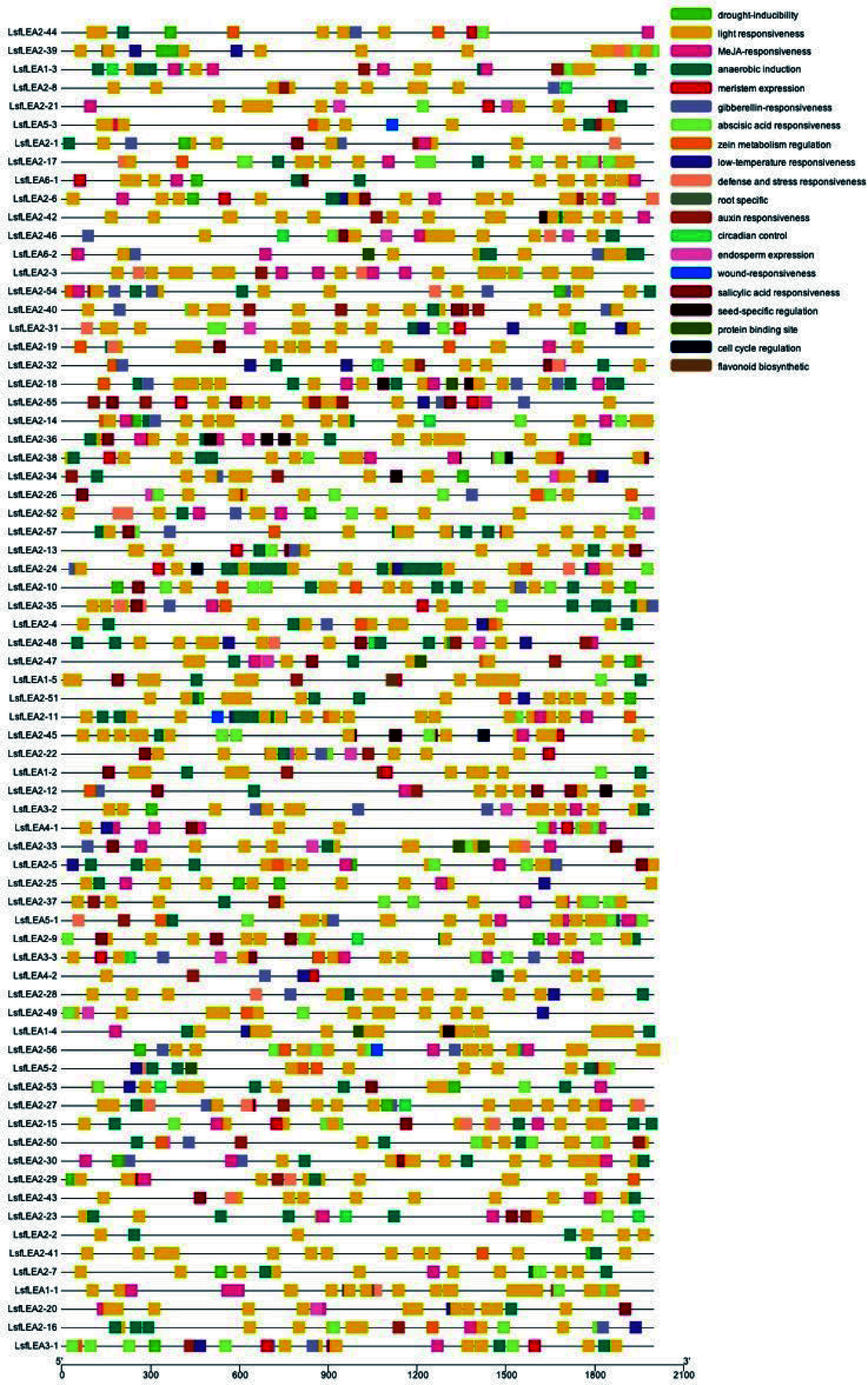
Cis-acting elements analysis of *LsfLEA* genes. The patterns in different colors on the right represent different cis-elements.

The presence of multiple distinct cis-elements in gene promoters may indicate that these genes have different functions. We identify cis-elements that regulate phytohormone response in the promoters of *LsfLEA* genes (Fig. 4). These elements included abscitic acid (ABA)-(ABRES), auxin-(AuxreS), methyl jasmonate (Mejares), and salicylic acid response elements (SARES). Upon further investigation, we found that the promoters of some *LsfLEA* genes boasted ABREs and MeJAREs, which could be activated by ABA and MeJA, respectively. We also uncovered a plethora of cis-elements linked to the plant's response to stress, including drought response elements (DREs), light response elements, MYB binding sites (MBS), and MYC binding sites (MYC). Perhaps the most fascinating discovery of all was that the *LsfLEA* genes promoter was heavily enriched with light-responsive elements, accounting for a staggering 87% of all cis-elements identified. The nucleotide sequences of 79 *LEA* genes in hybrid sweetgum are given in Supplemental Table S2.

### Expression profile analysis of hybrid sweetgum *LEA* genes in different stages of somatic embryogenesis and different tissues

**Figure 5:**
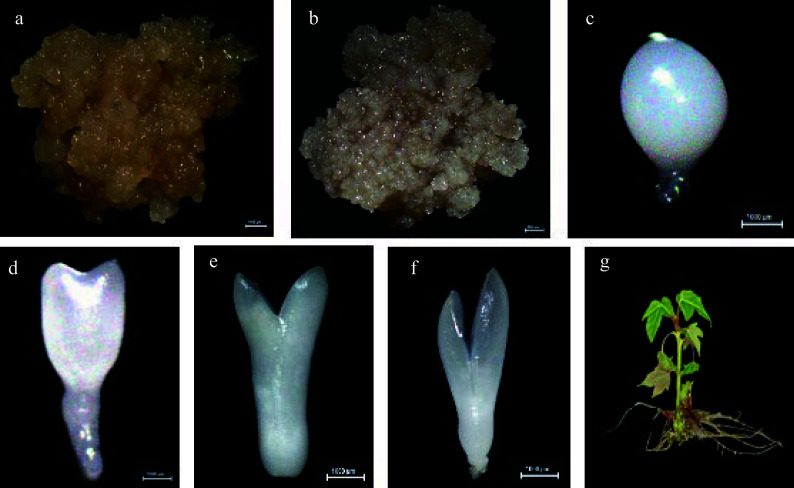
The cultures during hybrid sweetgum somatic embryogenesis and different tissues. (a) NEC: non-embryogenic callus. (b) EC: friable-embryogenic callus. (c)-(f) Somatic embryos at different developmental stages globular embryo (GE), heart-shaped embryo (HE), torpedo-shaped embryo (TE), cotyledonal embryo (CE). (a)-(f) Bars = 1000 μm. (g) Different tissues in root (R), stem (S), and leaf (L) of hybrid sweetgum.

**Figure 6:**
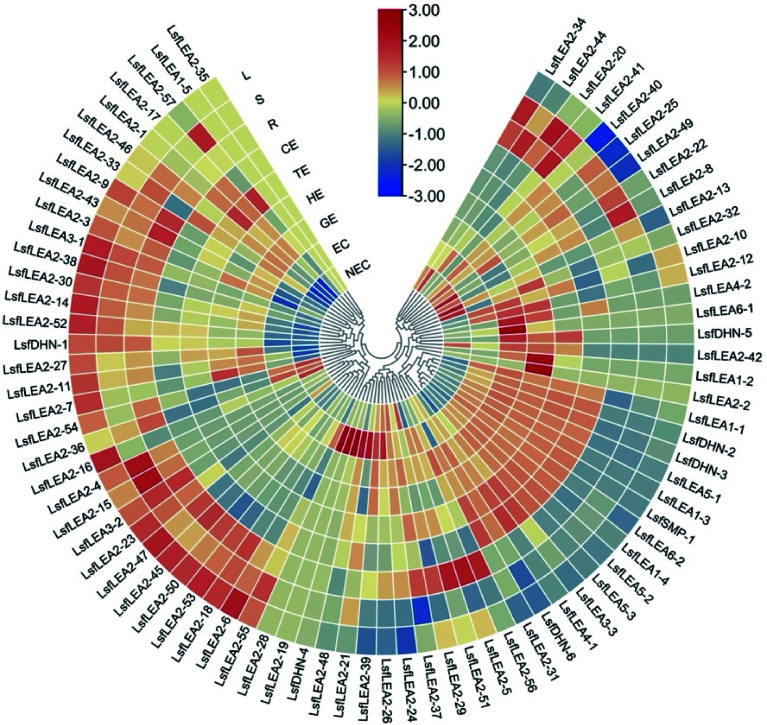
Heatmap of the differentially expressed 79 *LsfLEA* genes during hybrid sweetgum SE and different tissues. The heatmap was clustered by TBtools software. The heatmap indicates the gene expression level by Log2[FPKM] with a color scale, each row represents a single gene, the IDs are indicated to the outside of fan, and each circle represents a sample. NEC: non-embryogenic callus; EC: friable-embryogenic callus; GE: globular embryo; HE: heart-shaped embryo; TE: torpedo-shaped embryo; CE: cotyledonal embryo; R: root; S: stem; L: leaf.

Non-embryonic callus, embryonic callus and embryos of different forms of hybrid sweetgum were taken as samples. After cotyledon embryos were formed into seedlings, root and leaf samples were taken as samples (Fig. 5). Gene expression levels were measured in a total of nine periods. To investigate the expression pattern of the *LsfLEA* gene in different tissues, RNA-Seq analysis of the *LsfLEA* genes were performed (Fig. 6). The findings are indicative of the fact that the *LsfLEA1* genes are predominantly and primarily expressed in NEC, EC, GE, HE, TE, and CE stages, with conspicuously low or practically negligible expression in the root (R), stem (S), and leaf (L). It is worth noting that *LsfLEA1-5*, on the other hand, is not expressed in any of the tissues. In stark contrast to the tissue specificity of *LsfLEA1-5*, the expression of the *LsfLEA2* genes were not delineated to any particular tissues, yet intriguingly, *LsfLEA2-35* is not expressed in any of the tissues. Moreover, the *LsfLEA3* genes were detected to be expressed in all stages, and the expression of *LsfLEA3-1* in the root (R), stem (S), and leaf (L) was conspicuously and remarkably high. Conversely, the expression of *LsfLEA3-3* was most pronounced in GE, HE, TE, and CE. Notably, the *LsfLEA4* genes were demonstrated to be expressed minimally or not at all in any of the stages. The expression of *LsfLEA5* genes in GE, HE, TE, and CE were manifestly and significantly higher than that observed in other stages. Finally, the expression level of the *LsfDHN* genes were determined to be higher in GE, HE, and TE, with the expression of *LsfDHN-6* being markedly and notably higher than other *LsfLEA* genes in all tissues. Additionally, among the *LsfSMP* subfamily, the expression of *LsfSMP-1* was highly and remarkably pronounced in GE, HE, and TE, while being very low or even completely absent in all other tissues.

### Validation of hybrid sweetgum *LEA* genes by qRT-PCR in different stages of somatic embryogenesis and different tissues

**Figure 7:**
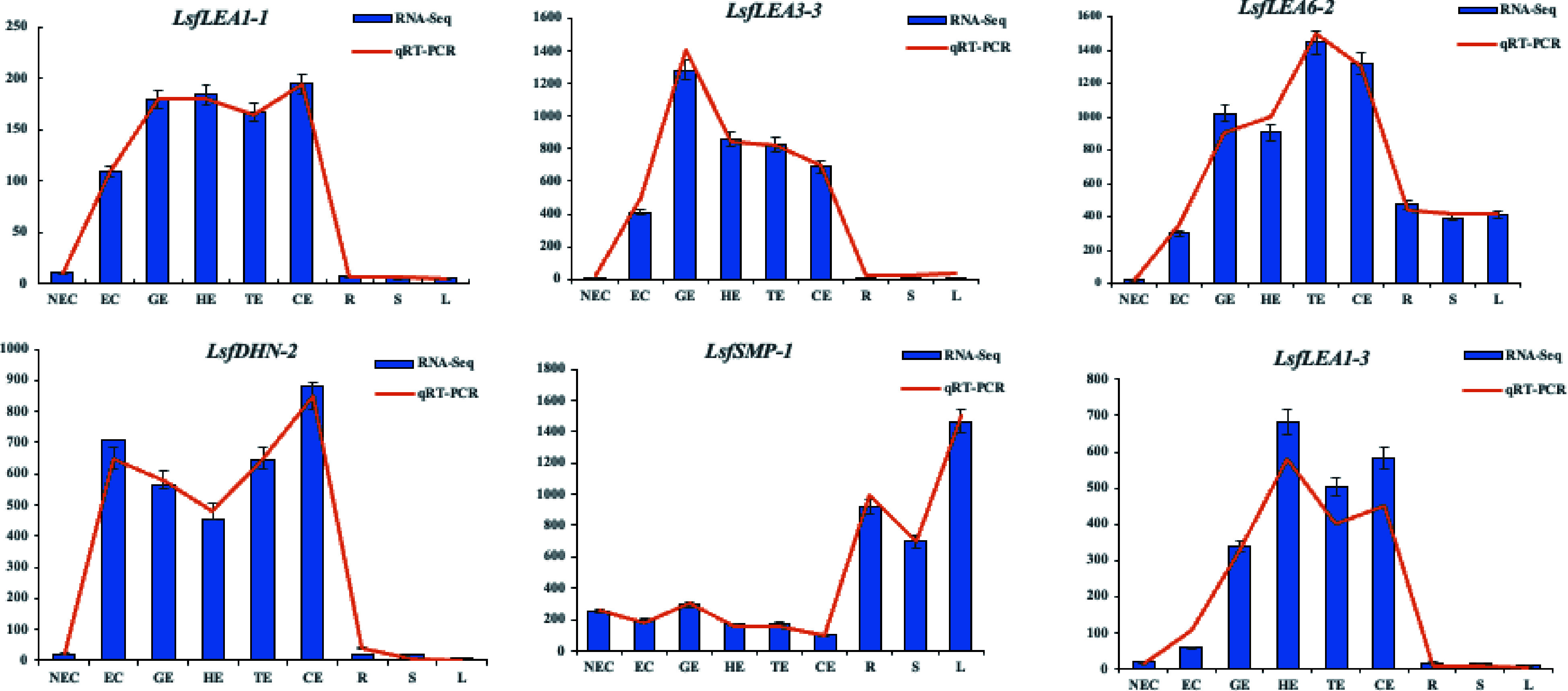
Transcriptome expression (FPKM value) and qRT-PCR value of six *LEA* family genes in hybrid sweetgum at different stages during somatic embryogenesis. Relative expression values were calculated using the 2^−ΔΔCᴛ^ method with apple *EF1-α* as a housekeeping gene. NEC: non-embryogenic callus; EC: friable-embryogenic callus; GE: globular embryo; HE: heart-shaped embryo; TE: torpedo-shaped embryo; CE: cotyledonal embryo; R: root; S: stem; L: leaf.

The results of the quantitative real-time polymerase chain reaction (qRT-PCR) analysis presented in Fig. 7 exhibit a remarkable variability in the expression levels of the hybrid sweetgum *LEA* family genes during somatic embryogenesis and different tissues. Intriguingly, the transcriptional levels of *LsfLEA3-3*, *LsfLEA1-1*, *LsfLEA1-3*, and *LsfDHN-2* displayed negligible or scanty expression at the non-embryogenic callus (NEC) stage, but were conspicuously augmented during somatic embryo development, eventually culminating in the attainment of the highest expression level at this stage, which exhibited a precipitous decline in the nutritional organs (R, L, S). These results suggest that these genes may promote somatic embryo development and confer a pivotal function in the procurement and sustenance of somatic embryogenic competency. Moreover, *LsfLEA6-2* exhibited persistent or accentuated up-regulation throughout somatic embryogenesis, whereas it was concomitantly down-regulated in NEC and nutritional organs (R, L, S), thereby underscoring the pivotal role played by these molecular markers in the context of somatic embryogenesis in hybrid sweetgum. In sharp contrast, the gene expression of *LsfSMP-1* was observed to be comparatively lower during NEC and somatic embryo development (GE, HE, TE, and CE stages), but up-regulated in nutritional organs (R, L, S), pointing towards the active participation of this gene in the overall process of somatic embryogenesis and its subsequent development. Meanwhile, qRT-PCR verification of genes related to somatic embryogenesis also demonstrated the high correlation between RNA-seq and qRT-PCR data.

### Subcellular localization of LsfLEA 1-3 protein

**Figure 8:**
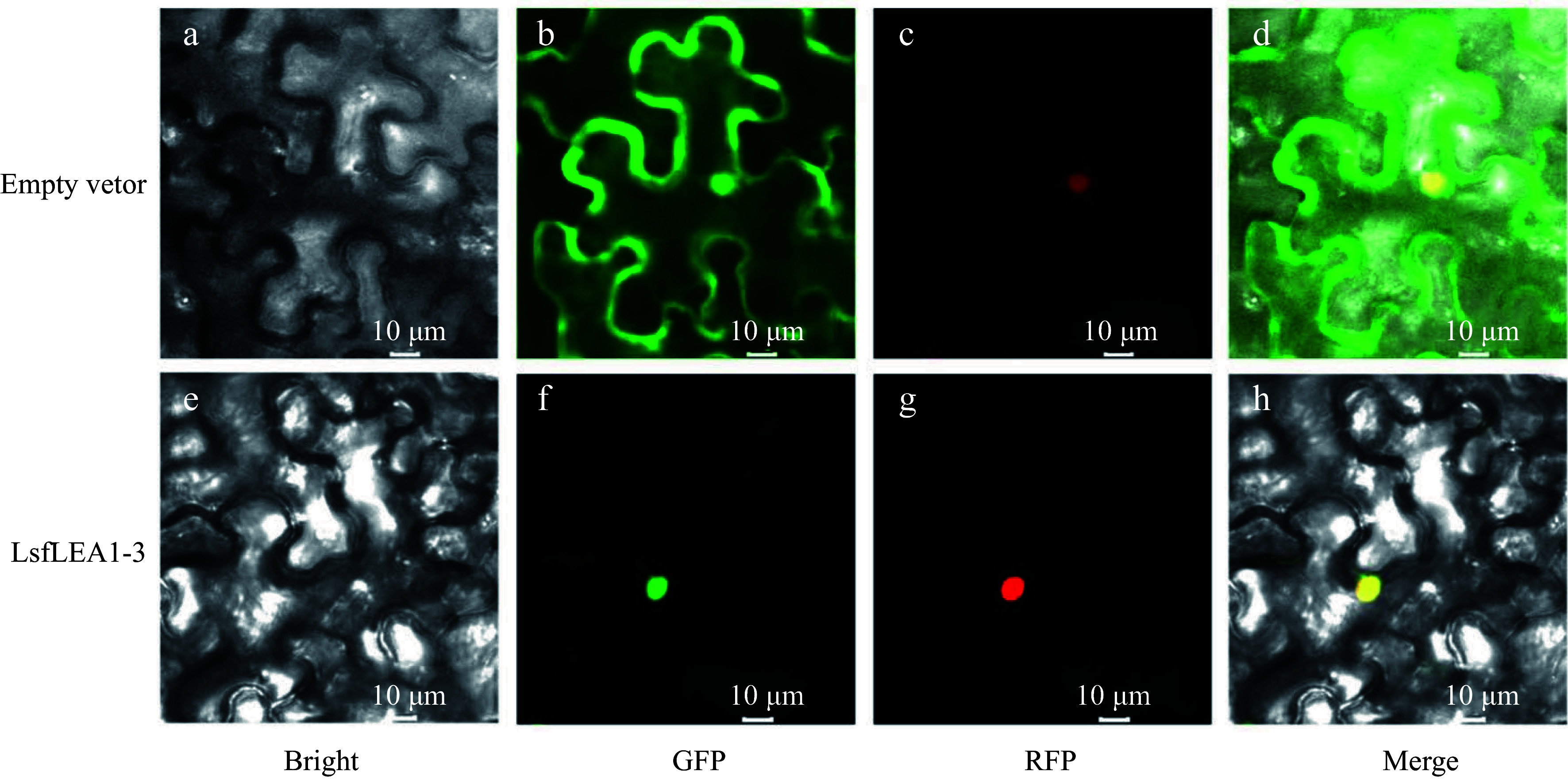
Subcellular localization of LsfLEA 1-3 protein in *N. benthamiana* leaves. (a) GFP empty vector in bright field. (b) Empty vector in GFP green fluorescence. (c) Empty vector in RFP red fluorescence. (d) Empty vector in merge. (e) LsfLEA 1-3 protein in bright field. (f) LsfLEA 1-3 protein in GFP green fluorescence. (g) LsfLEA 1-3 protein in RFP red fluorescence. (h) LsfLEA 1-3 protein in merge.

To verify the prediction results of LsfLEA1-3 protein subcellular localization, to begin with, the successful cloning of the full-length CDS of the *LsfLEA1-3* gene was accomplished, followed by the ligation of said gene into the T-vector for sequencing purposes. Following this step, the *LsfLEA1-3* gene was inserted into the pCAMBIA1300 vector, ensuring that these genes were fused with the GFP protein driven by the 35 S promoter upon expression in *N. benthamiana* leaves. Subsequently, the subcellular localization of the expressed proteins was observed and analyzed in a rigorous manner. The localization results obtained were markedly striking, with the GFP protein devoid of any gene insertion being expressed in various organelles in *N. benthamiana*, whilst the GFP fused with LsfLEA1-3 protein was exclusively expressed within the nucleus. This phenomenon effectively proves the expression and functional operation of LsfLEA1-3 protein within the nucleus (Fig. 8).

## Discussion

### Molecular characteristics of *LsfLEA* family genes

The *LEA* family genes have been the subject of investigation across many different plant species, however, this gene family in the hybrid sweetgum whole genome has been sorely lacking. In this study, 79 *LsfLEA* family genes were identified and separated into eight unique subfamilies. Conserved structural domain analysis revealed that each member of the *LEA* subfamily contains its own unique set of conserved domains, which have been observed in the likes of *Arabidopsis thaliana*^[4]^, *Oryza sativa*^[6]^ and *Triticum aestivum*^[46, 47]^, and these results suggest that LsfLEA proteins may have group-specific functions.

Furthermore, upon closer inspection, the conserved motifs observed in each *LEA* subfamily suggest that their members may have originated from gene amplifications within the subfamily^[48]^. Our results also indicate that the *LsfLEA* genes contain very few introns, which is consistent with other plant species such as *Brassica napus*^[5]^, *Camellia sinensis*^[49]^, and maize^[50]^, the low number of introns facilitates the rapid expression of the *LsfLEA* genes and functional protein production under abiotic stress.

Subcellular localization results of the LsfLEA protein revealed its widespread presence across several subcellular compartments, namely the mitochondria, nucleus, cytoplasm, cell membrane, and chloroplast (Table 3). Interestingly, these observations are corroborated by previous reports on *Arabidopsis thaliana*^[4]^ and *Solanum lycopersicum*^[10]^, lending further support to the notion that LEA proteins from hybrid sweetgum are ubiquitously distributed across cells and their corresponding tissues, serving indispensable roles in the operation of all cellular compartments during ontogeny and amidst stress circumstances^[51]^.

### Expression pattern analysis of *LsfLEA* genes

It is a well-established fact that the *LEA* genes constitute a pivotal component in facilitating the growth, development, and response to stressors in the plant kingdom^[52]^. Previous results have demonstrated that *LEA* genes are involved in the plants' reaction to abiotic adversities, most notably encompassing drought, low-temperature constraints, and high salinity stress^[53]^. Somatic embryogenesis, a developmental process that involves the formation of embryos from somatic cells, has gained recognition as a rapid and efficient means of propagation for woody plant species. Moreover, *LEA* genes were considered to be involved in somatic embryogenesis in cotton^[54]^, white spruce^[55]^ and sweet orange^[56]^. We identified the cis-elements of *LsfLEA* genes, which have been implicated in plant growth and development and stress response, including ABREs, AuxREs, GAREs, MeJAREs, SAREs, DREs, MBS, and MYC (Fig. 4). Similar cis-elements have been detected in the *LEA* gene promoter in *Arabidopsis thaliana*^[4]^and *Prunus mume*^[13]^. It has been widely established through previous investigations that the LEA protein exhibits a remarkable capability of being reconstituted by the CBF/DREB transcription factors, which has been found to play a pivotal role in the plants' intricate response mechanisms to the daunting challenges presented by the insidious cold and drought stressors that often threaten their survival and vitality^[57]^. The up-regulation of *LEA* gene expression has been found to be intricately linked with the overexpression of *LBDREB*. This phenomenon highlights the significant role played by *LBDREB* in regulating the expression of the *LEA* gene. It is important to note that the overexpression of *LBDREB* has been observed to elicit a robust response in the *LEA* gene expression, leading to its up-regulation^[58]^. In the intricate and multifaceted world of cellular biology, the regulation of proteins plays a crucial role in maintaining proper function and balance within the system. One such protein, LEA, has been found to be subject to the controlling influence of various transcription factors, including BHLH, MYB, and BZIP. These intricate regulators, with their multifarious modes of operation, serve to modulate the expression and activity of LEA protein, thereby contributing to the dynamic equilibrium of the cellular milieu^[59, 60]^. *AtABI5* regulates LEA protein accumulation during dark-induced leaf senescence^[60]^. The transcription factor *TABHLH49* emerging as a key player in the regulation of the wheat dehydration protein WZY2. The modulation of WZY2 levels by *TABHLH49* has been shown to confer significant tolerance to the plant under conditions of water scarcity^[59]^. The transcription factor *LcMYB2*, which has been shown to play a crucial role in the activation of gene expression by directly binding to the promoter of *AtLEAA14.* These results suggest that the *LsfLEA* genes may be extensively involved in the response to abiotic stress in hybrid sweetgum through regulation of transcription factors. The *LsfLEA* genes have been found to encompass the majority of *LsfLEA1*, *LsfLEA4*, *LsfSMP*, as well as members of the *LsfLEA3*, *LsfLEA5*, and *LsfDHN* subfamilies. This discovery has revealed the intricate and multifaceted nature of the *LsfLEA* gene family, with its diverse and nuanced subfamilies contributing to a complex network of genetic regulation and expression. The presence of these various subfamilies highlights the importance of a holistic approach to the study of the *LsfLEA* genes, as well as the need for a deep understanding of the intricate molecular mechanisms underlying their function. In our quest for understanding the intricate workings of somatic embryogenesis, we endeavored to unravel the complex expression pattern of the *LsfLEA* genes across diverse stages of development. Our findings revealed a relative upsurge in the expression levels of *LsfLEA1*, *LsfLEA5*, and *LsfSMP* genes in the roots. Additionally, our investigation unraveled a relatively high expression level of *LsfLEA1* and *LsfSMP* genes in the GE, HE, and TE stages of development. From these results, we infer that *LsfLEA* genes are intricately involved in the elaborate processes of somatic embryogenesis and development. It is noteworthy that these genes may fulfill divergent biological roles at different developmental stages, underscoring the complexity of this phenomenon.

## Conclusions

In this study, the genome of hybrid sweetgum has revealed the presence of 79 *LsfLEA* genes, partitioned among eight subfamilies. The phylogenetic relationship, exon-intron structure, conserved domains and cis-acting elements of *LsfLEA* genes were analyzed. Transcriptome and qRT-PCR results showed that the expression of *LsfLEA* genes had certain specificity, which preliminarily revealed the function of *LsfLEA* genes in somatic embryogenesis. Subcellular localization results confirmed that the *LsfLEA1-3* gene was localized in the nucleus. These results provide a pivotal reference point for future bioinformatic analyses and in-depth investigations into the profound and intricate function of *LsfLEA* genes in somatic embryogenesis.

## SUPPLEMENTARY DATA

Supplementary data to this article can be found online.
