# Supplementary material for: Genome-wide identification and expression analysis of late embryogenesis abundant (LEA) genes reveal their potential roles in somatic embryogenesis in hybrid sweetgum (Liquidambar styraciflua × Liquidambar formosana)
Source: For Res (Fayettev). 2023 May 29;3:12. doi: 10.48130/FR-2023-0012 (PMC11533890; doi:10.48130/FR-2023-0012)
Supplement: Supplementary file 1 — Supplementary data to this article can be found online. [file FR-2023-0012-S1.zip › 10.48130_FR-2023-0012-Suppl-TableS2.pdf]

S2:The Nucleotide sequence sequence of LsfLEA genes

>LsfLEA2-12
ATGGCGGATTCTGCAATACGCAAAACCGAGGACAGCCCGCCATCATCAAAGCCTTCCT
CTAATCCCACATCAAAGCCCGTTCGCCACGTTGTCTTCTCAGAAATCCCTCGTAGACCT
CACAAGCCCGACAAGGAACGGCGAAGAGGCCGCTGCTGTTCGGGTTGCTTCGTTGTC
TGTTGTGCATGGGGCTGCATGATTGGCTTCGCTCTCGTTCTCGTCCTCCTCGTCGTTGC
GGCCATTTTCGTCGCCTTCCTCCATTCTGAAATGCCAGAAGTCCATGTAGCGAGGGTGT
CCATCCCTTCGTTAGATGTGGTGACGGCCTCATCGCAACAGGCGCTCTTGTCTGCTGAT
GTGGATATTGTGGTCGAGGTCTCCAACAAAAATGATAGAGCTAAGCTCTCTTACGGCAC
CATGACGGTGGACGTTTCGTCGGAAAAGATCAGCTTGGGTCAGACTAAAGTTGTTGCG
TTTTCTCAGAAGCCTAAAAATTCGACGGAATTGAAAATACACACCAAGGTTACCCGGT
TGGCAGTGGACAAAGAGGATGGGGCATTCTTGAAATCAAATGTCAAAGACGGGGAGA
TGGTTTTTGATGTTGTTTTAAGCGGAACCATAGGTGTGGGCGTGGGTAGGTTCACCATA
AATGGCTTGCCCGTCACCATGGCATGCCATGGCATCCAGCAATCTCAGGTTGATATCGG
ACAGGAGCCCAAATGCAGTGTTAAAATGTTCTCCTTCAGGTTATATACTTTCCTGTTTAT
ATTAATTATACATTATTTATAG
>LsfLEA5-3
ATGGCATCAAACCAGAAGCAGAGAGAAGAACTTGACGCCAGGGCTAGGCAGGGAGA
GACTGTGGTCCCTGGTGGTACTGGTGGCAAGAGCCTCGAAGCCCAAGAGCATCTTGCT
GAAGGTCCCTCTCCATCCCTATCTTTGTTTTAGTCAGCATATATATGTCAATATTATTGATA
TCAGCACTATGCATTGAAGTTATTCTTTATGCGCAGAAAATCGCACTATCAATACATATT
GACCTGAAAATTATTTTGTACCAGAGTATGTGCATGTTAAGTATGTATAGGCCGGTATTA
TTATTATTTATTTTTTTCTAAGATTTTTTTATGTGAAGCTTTTTTTGAGTTTGGGCATCTT
GTCCTTGGTGATGAATTGAAGGAAGGAGCCGAGGAGGACAGACGAGGAGGGAGCAG
CTGGGGACAGGAGGGTACCAAGAGATGGGCAGCAAAGGAGGACAGACGAGAAAGGA
GCAGCTGGGGACCGAAGGGTACCAGGAGATGGGCCGCAAAGGTGGACTAAGCACCA
CGGACCAGTCGGGCGGGGAACGCGCAGCCGAGCTAGGGGTCGACATTGATGAGTCTA
AGTTCAAGACTTCATCTGGCTAA
>LsfDHN-5
ATGGCAGATCAGCACTTTCAATGCCATGAAAAGGAACCAAGAGATCGTGGGATGTTTG
ATTTCTTGGGGAAAAAAGAGGAGAAACCTCAAACGGAAATGTCCATGACAGACTGCA
ACTTGGCATGCCTGTCAGAACCCCAGGAGAAGAAAGAGGTAGAGAAGCCTACTCTTG
TCGAAGAGCTTCGTCGGTCTCATAGCAGCTCTAGCTCGGTAAGCTTTCTCCAAATTATC
CTATTTCAACACCACATCACCATGACAAAGAAATATTTGTTGAACAACCATCCCATGTG
TGGAAGCTGCCACACTTCTAGACCAAATGAATCAACATAGTGCTCGCCGTATTAGAAA
AAGTCACAGACTCACATTACCATACTTATCTTTAAGACTCATGACTTTTTTCCTAATGGT
TAAAGAAGGCTTGCTTGATATGGTGTGGATGTGTGGTTGTTTCCAGACCAGACCAAATA
TACGCTATATCCAGATTCCAGAGTATTCAATCAAGTTATGAAAATGCAGAAACAAATGA
CAAAATTTTAGCCAATGCTGCAAGAAATATATGACATAAGAGCACAACGTTATAAACAT
AGAATTCATCAATCTTTATTGACAAACTCCTGTTGCTTGAAACCATTCAGTCAAGTGAT
GAAGGAGGAGGAGAGAAGAAAAAGAAGAAGGGACTAAAGGAGAAGATTAAGGAAA
AGATGGCACGCCAAGGAGAAGAGGAAGTTACTGAGATTCCGGTTGACAAGTGCGATA
ACATAGTGGATGCAGAAACAACACACCCAGAGGAGAAAAAAGGTATTCTAGAGAAGA
TCAAAGAAAAACTTCCAGGTCAGCACAAAAAGGGCGAAGAGGGTGCTACCACAATTC

CACCTGAGTGTGCCCCTGTTGGGCACCCTAGCGAGGCAGAGGAGAAGAAAGGAATCT
TGGAAAAGATCAAGGAGAAGCTGCCTGGGTACCACAAGAATGCAGAGGAAGAAAAA
GAGGAGAATTGA
>LsfLEA2-49
GTGAAACGTATGGCAGTGACAATGCATGCGAAGTCGGACTCAGAGGTTACAAGCCTCG
ACGCCTCATCTCCACCGCGGTCGCCTCGCCGGCCGCTCTACTACGTGCAGAGCCCGTC
GCAGCACGATGTCGAGAAAATGTCATACGGGTCGAGCCCAATCGGGTCGCCTCACCAC
TACCACTGCTCGCCGATTCATCACTCCCGCGAGTCGTCCACCTCAAGATTCTCCGCCTC
CCTCAAGAACGCCCACCACCACAAGCACTCCGCCCCGTGGAAGAAGATGCACCGTGT
CGTCGACGTCGACGACGATGACGAAGAAGACGGCGACGGCGATGGTGGAGGTTCCGC
CCACAAAGTGAGGTTGTACTTGTGCTTCGTCGTCGGGTTCGTTGCCCTGTTTACGGTGT
TCTCGCTCATTTTGTGGGGCGCCAGCAAAACCTACAGGCCTAAAATCTTCGTTAAGGTG
CGTTTCGCGTCTGACTCAGTGGTGTTTTGAGTCAACTCAGTGACTCGGTCGTCTGTTTG
GGGATTATACACAACCTTAGCTGTATATAGTATAAACGTCACACACTATATGACTCTTAC
TTTTGGTAGTGCGAGACTGCGAGGAGAGGATTGAATAGATTAATATTTGTCATCTGTCG
TGAATCTTTGTCCCCGAGTCAACTCAGTGAGTCAGTGCATTGGCTTTTATTAATAGTCTC
TGTTTGGTTGTGTGATTTGGCAGAACATTGTGTTCCGAAACTTCAATATTCAGGCCGGT
ATGGACAGTACAGGGGTGCCAACGGATATGTTGTCGTTGAATTCGACGGTCAGGATTT
CATACAGGAACCCAGCCACATTCTTTGGGGTCCACGTGTCCTCCACCCCGCTAGAACT
TTACTATTACCAGCTCAAGGTTGCCTCTGGACACGTAAGTAAATTTCCATTATCCAATTG
TTTTCCCTTAAGCAGTGATTTAAATATGTAAATGTTTTTTCTTTGAATGTCTATTTTATCC
ATGTTTTAAATATTAGTTGTTAGTTAATTCATGGGTAGCATGGTCATTTTGATTAATTGCT
TAAACTTGCATAAAATTATTCCGAATGGTTCCAAAATTGTCACGATTAGTTTGAGCCTG
AGGAGTCAAACTTGGTTGGTCTTGTTTTCTTGGGTGGTGGGGACACAGAGAGAGAGA
GAGAGAGAGGAGAACTTAAATGTGGCCAAATTGTCCCATTGATTGCGGTTCGTGCAAA
AGGGTGCAAGACTTTCAGCTACCATACCTGTCGTCCTGCAGGGATAATTATTGGAAATA
GTTTTCAATAGAGACTCGATACCATAGCGATGAAGGTCACCAACATGTGGGGTTTAGAT
GTTTCTGTTGGATGACCGTTATTGCAAAAGTAAAATAACAGAGACTATCGTAAGAGCAT
TTTCCCAATATCAATACTTAATGATCCATAAAATTTGATTGATAAGATGACTCGTATGCTA
ATAAAACATGGTTCCTCTATCTCGGGTAGAGCTCACACTCTATTAGTGTATGGATCTTTT
TGTGGGTCAATTTTTCCCCTTTTTGGATCATTAACTATTTTCCTTTTCCTAATTATTCTACA
TTATCTCGTCGGATGATGGTTTTGGTGAGGTAAAACATTATCGATATGAGAAAATAAAG
AAAATAAATATTACGCCCATTTGTATTGACTAGAAAAATGATTTGGCAAACCGTCTCTTT
GATTCTTTTATATGTACTGCTCTTCACATCGCGTGATCCACGATCGCGTAAACGCACGAA
CATGGGTCCCATCTCATGTGAGAGAGACGGCACATGGAGGCGGTCAAAGTGGCAGTC
CGTCTAGCATTGTCTTCTTGGATAAAGTCATGCTAGTATGACAAACATAAAAAATCACA
GTTATGATAGCCTATTTATTCACCAGAATTCAGAGAGAATATGATATCTCTGTTAAGTGG
AGTCCACGCAGGTCATTAAGTTCCTCAAATTTATGGTGGACAAGTGGCTGTCATGGTTG
TCATTTCCTTTTGTATGTTACACTAGGATTTTCTTAGTGAAGTTGTAAGTGTTCTTTCTCG
CGTTTTGAACAATACAAATAAGAAGGAAAAGGAAGGACAAACTAAGGAAGAAATAGG
CTCTGTATACCTCGTGTGTACTGCTTTCAGCTCATTTCTTATGCTGTAGTATAACTTTCTT
CACCTTTATAAGGAAAACAAGAAACCAATAAAATTTTAAAAAGTAGTACAGTATGAATT
TATCCTTGCCACACCCTTGTCCAAATGTAGGAGATTGGCGATATAGCATTCATATTTAGG
GAGAAATTATGTTATGGGGTCAAAATGCCAACATGGTGGGAAACTTTATCTAATATGTG

ACACATGAGTATTGTGACAATGTCAATATCACATTATCAAGAAATAAACTTAATTTATCA
CTAGTTTACAAATTACAACATTTCCACGATTCAAGAGTTGAAAGGGTATTTTGACCAGA
TTTTGAAATTTAAATTTGAAATCTGTCTTGCACGCATACACACACATTGGCCTTCCACA
ATTCTGATTGGTTTTTGCCCTTTGCTATCTTCACTATGTATGGTCTACGATGATTTTATCG
ACACAATTTTGTTTACCATATATTGTCAATAGTTGAGCTTTCTACAAGTGCCATTAACCT
TGTAAATGTTTGAAATTGGGTAGAACACCTTTGTTATATGGAAACAAACATTATAATATC
GTTAATCTTAGACTTAATATTGAAATTTGGTAACTTTGTTGATGATTTAATGCACAACTA
AAACAACAACAATTAAATTGGACATGCTGTCTGGATTCAAATTATGAATTTTGGTAAAA
AATTTTGCTAAGCTCTTGAATTGTGGAAACATAGTGAAGTTTAATTGAGTTTATTTCTTG
ACAATGTGGCACTAACAGCATTACTATGCTTAGGTGACACATAGTAAATGGATCTTCCC
ACCACGTTGCGTGTGCTAACTTGGTGAGAAGCTCCCAAAGGATAATTTCTCTACAGTG
AGGAAGCTGATATGACTTGAATTAACAAGGTTACAAACCTTGTTAATGGCACTCATTTA
AGCTGCATGCAGTATGCACTTGGTTAGGTTAGGACTTAGGACTATGGTGTTGTAGTTCC
CCCTTTCTTAAACTGCTTATCATTTCAAAAACATGGTGTCAATAATTAAAATTTTAATATA
GTACAACACTACAACTCATAAACATAGTTGAACTTTATTCTAACTTTTCCAGTTGGACA
CCTTACATTTCAACCTAAGAGAATCAATTCTTTGGTTCATAGATATTGGCTTTACAAAAG
GTGTGTTGCTTGTCAAGATATTGTGTTTTTTATTTTTATTGATTTGGGTCTTTAGTGAAAA
TTTAACTGTAGACCCACCCACCCTTTCAAATGTCTTTACCACTGTGCTATGAGCTCAGA
TAGAAGTCATCTTCCCATGGTGTTAGAGGTAAAGGCATCTGGAGGGTTGGGTGGATCTT
AGTAAAATTCTCATTGTTGATTGAAGAAAATTGCATTTTTCTAAGAAAACTCTTTTTATA
GCCGCTATGTATTTTAGTTTTAAGTAAGCTCTATGTTTCGGTATTTTTCAAGATGCTTGCA
TAATCAAAGATTCGTTTGAATCCAACTTTTGTGATAATAGAGGTAAAATGATTTTCATTG
ACCTACTTAAAATTGGAAGACAAGGTGAAAAACAGCACCTCATTTTACCTTTACTATAT
GTAATACAGGTTGAGTAAAATAAATCTGAATTATGTGAGGGGTGTTTCAAATTTACAAG
AATTAATGGGTTTTTCTAAAAATACCTTAAAACACAAGGGTTATGTGAAAAGGTCCCTT
ACTAATATGAGTTCCTTCTTGTTAACCATTTTTTTATTTTTGTATAGATTTTATACAATTGG
CCAAAAACCAGGTGGTCTTTTGCCACAGGTGGTCCATGGTTAGGATCAATGTATGAAG
ACAAAACTATTGAAAGATTTGAATTTGTTAAGACACATGCCTTGATCCTGACAATGGAC
CACCATGGTCCATTGAACACTTGAGTTTTTCCTTTACTCTTTGATGGAAAACAAAAATC
CTAATATTATAGACCAATTGGTATCACAAGCATCACAACCCATTCATTACCATGAATTTTA
ACAAGGCATGATGTCATAAATGGGACCCACATCACAACAAATGACATCATGCCTTGTCA
AAATTCATGGTGAGGGTTGGGTTGTGTTGTTTGAGATGTCTTTTTAGTATGATAATAGGA
TAACTCAAAGACTAATACTTCATGTCTGCGTTCTATTTTTGGTATCTAGAACATTCATAA
CATTTTGAGTTGATGAGTTTTAAACATTTTACTTTAACATTATTTTTCAATCAAATTTTAT
TTAGGTTTTAATTCTTCACATTTGATTATAGTTATATGCTGAAATCATCAAATTGAAATGA
TATTTAGACCTGGCTCATAAGTGGACTTAACTGATAATGATTCCAAATAACTTCTCACTA
TAGCTGGAAGCTTAAAGTATAGATAGTGAACGTGATCATGTGATCCAGACCATAGAATT
TTTTTCCCATAGAAAACAGTACACTATGTAAGTTCTCCATGACCACACTCCTCTTTGGTA
ATTATAGTCATCAAATCTTGCCATGTTTGTATCTATAAAAGAAAAATAAAATAAAATAAA
GACCCATCAAATCTTGTTGGTGTTACTGACCCAAATCTTGCCTGATTTTTATGTATTGCT
GATCAAACAAGCGGAACCCTTCAAAGGTTTGTCTTCTTATTTTTCTGATAGTGATTGCA
AGATTCGAGGGACTATGAAAATTATGGTTGACAATAAGAGAGTAAGCTTAGCCCTACTG
TGATTTGACAGAGAAAACCAAGTATGGCAAGCAGGTGACCTAGGGCTACCAGTAAGTA
AATTTATACAATCTGATTGGCCAAATGCACTTGGCAGGCTTTGTCAAATCCATTCTTCTA

TGAGCTTCTTTTACAGAAAGCAATCTCTCAATAATCACACCCCCTCCCAACATTTTTTG
TTAATATTTCAGCAATTCCTTGGGGGGTTGGCATTGTCTTTCTCTGATCATCTTTATAAAA
AATGTATTAAATAGATGAAGGAGTTCTATCAATCAAGGAAGAGCAATCGGAATGTTGTA
ACAATGGTGCTAGGATATCAAGTGCCACTCTATGGTGGGGTATCAGTCCTCGGAGGTGC
TAGGAATCACTACGAGAATGTCGCAGTGCCACTGAACTTGACATTTGTGATGAGATCC
AGAGCCTACATTTTGGGAAAGTTGGTGAAGTCCAAGTTTTACAAGAGAATCAGATGCT
CTGTCACTTTAAGAGGCAATCAGCTTGGCAAGCTCCAAAATTTGACGGATTCTTGTATT
TACCAGTGAATGGTCCATTCCTTTTGGTGCAGTTATAAATAGTTTGGATGGCTTAAGACA
CGTGGTTGTTAGCTTTATTGCCTTTGAATATTTTCCGCAGCCATCTTACGAACTTGAAAT
TTCATATACTTGAAAGTGGTAATTAGGATTCAGCCATTTTTAGCGCTTAATAGTCAGGTA
GAAATGTTAGCAATTTATGATTCATAGTTGCTAACTATATATCATTTACAATGTAAATTTG
ATTAGTTGTATATAAAACTTCTCTTAGAAAGCATAAGAATTAGTTGCCTTTCAGCCATTT
CTGCTGATGCATTTGGCATC
>LsfLEA2-35
ATGGCTAGTTGTAATCCTTCCCGCCGGGGTCTTAAAATCTGCTGTGGTGTAACGGCGAT
TTTCTTAATTATTGTTGTAATTATTTCTGTAACCTTGTCACTTACCATATTCAAGCCAAAA
CAACCCCAAGTCACTGCCCACCCTACAAGTCTAGAAAACATTCAATTCACTGTGGTCC
CAGTCCCAAGCCTAAATATCACATTAGGCATGATGGTTTCTATCGAAAATCAAAATTATG
GGAGCTTCAAGTACAAGAACACTACAGCTTATGTTAATTATCATGGGGCATTGGTGGCT
GAAGTTCCAATTGAGCATGACAAGGTTCCTGCACGCGGCAAGGTTAACATTACCACTT
CGGTTGAGATCATGGGAGATAAGTTGATATTGAGTCCCTATTTTTGGGATGATGTTGCCG
CTGGGTGCTTGAATTTGACATCAATGGCTACCTTGCATGGGAAAGTGAGTGTGCTTAAG
ATCTTGAAGATTCGTGCCACGGCTTTTAGTACTTGTGAGATCTCTGTTTTTGTTCAGTCT
CAGAATATTAAATCCAATTGCCAGTCCAAAATCAAGCTGTGA
>LsfLEA2-56
ATGGCAGATAATCAGAGAATCCATCCAGTGGTCCATGACCCCGAGGCGCCACACACAC
CGACGGCACCTCTAGTGCCGCGCGGCTCGTCAAAATCAGAGAAGGGGGCTCCGGCTG
ATCAATATCATCAAGTACCATTGCGCCGTACCATCCCGGTCATGCACTCGAAACCCCCC
AAGAAGAGAAGCTGCTTTTGCAAGTGCTTGTGTTGGACGGTTTGCCTCCTGCTGCTCC
AAGTCATCATCGTTGGAATCGTCGGTGGAATCCTATTCCTCGTCTTCCGGCCAAAGCTC
CCAAAATACTCCATCGACAGCTTGAGGATTACGCAGTTCAACTTAGGCAACGACAACA
GCTTGAACGCTACCTTGGACGTGGCCATCACTGCCAGAAACCCAAACAAGAAAATCG
GAATATATTACGAAAGCGGCAGCCAAATAACGGTGTGGTACACGGGCACACAACTATG
CCAAGGGTCTCTGCCGAAATTCTACCAGGGTCACCGGAACACCACCATTCTGAACGTG
CCTTTGACAGGACAGACGCAGAACGCGACCGGCCTTTTGTCGACCCTGCAGGCGCAG
CAACAGACGGGTAGCATCCCGCTGAATCTCAGAGTTAACGTCCCGGTGAGGATCAAGC
TTGGGAAGTTGAAGCTCATGAAGGTGAAGTTCAGGGTGAGGTGCAGGCTGGTGGTAG
ACAGCTTAGCTGCTGATAATGCCATTAGTATTAAAAGTAGTAGCTGTAAGTTCAGATTG
AGGCTCTAA
>LsfDHN-6
ATCATTCAAAACTACAAGGATAGCCATATCCCAGAAAAGAATCACTTATTTGTCTCTAC
GATCAGTTTGATTTGAGTTTAGCATTATTTTCAGTTCTGGTTTTTTTGTTGAATCTCTTTG
GAAAATGTCGTACCAAGACCAATATGGAAACCCAGCTCGCCAAACAGACGAGTTTGG
AAACCCAATTCGCCAAACAGACCAATATGGAAACCCAATTCATCACACTGGCACCACG

GGTGAATACGGAGCTACCCACCCCTCCGGCACCACGGGTGGTGTGCTGGGCTCCGGCT
TAGGCGGGGGTCAGCATCAGCAACATCACCAGCAGGTGCAGGAGCACCAGGGCCTCT
CTGGCACACTTCACCGCTCTGGCAGCTCCAGCTCTGTAAGTATTTTTTTTACCTCATCC
ATCATGTCATTTTCATAATTAAAGAAATGTATTTGCCAAATTGCAGCACTCTAGTTTTAAT
TAGCTTTCTTTTCTGTTTTTTTTATTATTTAATATATATATATATATATATATATAAATAGATTT
TAAGACATTTTTGTATAACGGTTGCAGTCTGAGGATGATGGGCAAGGCGGGAGAAGGA
AGAAGAAAGGGCTGAAAGAGAAGATAAAGGGGAGGACTCCAGGTGGTGGGCACAAA
GATGAGTACTCGTCGGCCACCACCAACACCACCACTCCGGGCGTTTACGGTGCAGGGG
AGCAACATGAGAAGAAAGGAGTGATGGAAAAGATCAAGGAGAAGCTTCCTGGACAC
CGTTAGGTGCCTTTTAATTGACACAGTCTCTATATATAATATGATGCTTGTTCGTAGGCGA
AACGCATAGTCTAGGGGTCACAGATCATGTATAAGCGTTTTGTTACGGACAAGTTTCTA
TACTTTATAGTTGTGTGTAATAATAATGTATGCGTGGTGGCATATATAGGGCGCGCATCTT
TGTATGTACTTTCGATTATGTTTCTGCTTCGTGTGTTGTATTGTGACTTTCTATACCATATA
TATACTATGGTTTATTACTATGATTTGTTGAAGTT
>LsfLEA6-2
ATGGAAAAGAGTGGAGAGCAAATGGGAAGGAAGGGAGAGAGTGAGCAGAAGGGAA
AGTTAGATGGGCTTCCATTAGAGACCAGTCCCTATACCCAGAATTCTGATTTGGAGGAC
TACAAGCGTCAAGGCTATGGCACTGAAGGCCATCTCCAACCAAAGACCGGCCGTGGT
GCTGGCTTCACCGACGCCCCCACTCTTTCCGGTGGTGCCGTCTCTTCTGATGTCAAGGC
TTCCGCCGTTGATGCCATCAACCGCAAAGGGGTCCCCTAA
>LsfLEA1-3
AGAGATGAAAGTAGGCATAACCATAAAGCTTTTGTACTTTCATCTCAAACCATTTTTTG
CTGTATCTGTGTTTGCGTTTGTGAGAAAGAGAGAGAAAGAATGCATTCTGCAAAGGAG
AAGTTGAGCGACATGGCAAGTGCAGCCAAGGAGCATGTCGACATCTACAAGGCTAAA
GCCGAAGAGAAGGTAGAGAAGGCGGCGGCGAGGACAGAGGAAGAGAAAAAGATAGT
CCACGAGGGAAGAAAAGCGAAGGAAGCTCAAGCAAAGATGGAGCTGCACGAGGCCA
AAGCGAGGCACGCGGCGGAGAAATTGAACGCTAAGCAATCCCATCTTTATGGCCACCA
TGTCCATGAGCCACCGCTGGTGGGCGCACATCATAGGCACAATGAACCAGTGGTGGGT
GTCCATGGACACCATGGGGCGCAGCCTGTTGGAACTGTGGCCCCCTTTACCTTTACCGG
TGGTGGGCACACAGCATGGTCACAATGTTAATGAGCCGGTGGTGGGTGTCCAAGGACA
CCAGCCTGTTGGAACTGCCGCCCCTTTAGGTGGGGGCACAGTTCCGACTTACCCTCTC
GGAGGCCACCCTCCTCGACGTGAGTATATGTAGAGTTGGTGGTGGTGGTGGTGTTAGG
ACTAGCTTGTGGTTCGCATAGGGATGTTTTGCATCATAATGGGTTGGTTGCTTGTTGTGA
GATGTATACTGTAGCATGTTGTAAAAGAAAAATGCTAAAGCAAAACCTGCTCTATCCGC
T
>LsfLEA2-13
ATGTCTAAAGCACATAGACCGCCTTCAGGCCGTACGAACCTCGCTTCGTGCATAGTGGC
CACAATTTTCCTAATCTTCGTCGTCATCATCATCCTCATCGTCTTCTTCACCGTCTTCAA
GCCCAAAGACCCACAGATCTCCGTCAACGCCGTCCAATTGCCGGCATTCTCCATCAGC
AACAACACCGCCAATTTCACCTTCTCCCAGTACGTCTCTGTCAAGAACCCCAACCGCG
CCGTCTTCTCCCACTACTCCAGCACTCTCCAGCTCCTCTACTCCGGCAGCCAGGTCGGG
TTCATGTTCATTCCCGCCGGCAAGATCGAATCCGGCCGGACCGAGTACATGGCGGCGA
CTTTCACCGTCCTGTCGTTCCCTCTGTCGGTGGCGCCATCGGCGGCGGAGGCGGGGCT
CGGACCGCCGCTCTCCGACGTCGGTAATGGGTTTCGGTTGGGACCCACTTTGGAGATT

GAATCGAGGATGGATATGGCGGGAAGGGTTAGGATTTTGCACTTCTTCACACATCATGT
GGAGGCGAAAGCTGGGTGTAGGGTTGCCATTTCCGTGGCTGATGGATCTGTGTTAGGG
TTTCATTGCTAA
>LsfLEA1-5
ATGCATTCTGCAAAGGAGAAGTTGAGCGACATGGCAAGTGCAGCCAAGGAGCATGTC
GACATCTACAAGGCTAAAGCCAAAGAGAAGGTAGAGAAGGCGGCGGCGAGGACAGA
GGAAGAGAAAAGATAGCCCACGAGGGAAGAAAAGCGAAGGAAGCTCAAGCAAAGAT
GGAGATGCATGAGGCCAAAGCGAGGCACGCAGAGGAGAAATTGAACGCTAAGCAATC
CCATCTTTATGGCCACCAAGTCCATGAGCCACCGCTGGTGGCGCACATCATAGGCACA
ATGAACCAGTGGTGGGTGTCCATGGACACCATGGGGCGCAGCCTGTTGGAACTGCGG
CCCCTTTACCTTTACCGGTAG
>LsfLEA1-2
ATGCATTCTGCAAAGGAGAAGTTGAGCGACATGGCAAGTGCAGCCAAGGAGCATGTC
GACATCTACAAGGCTAAAGCCAAAGAGAAGGTAGAGAAGGCGGCGGCGAGGACAGA
GGAAGAGAAAAAGATAGCCCACGAGGGAAGAAAAGCGAAGGAAGCTCAAGCAAAG
ATGGAGATGCATGAGGCCAAAGCGAGGCACGCAGAGGAGAAATTGAACGCTAAGCAA
TCCCATCTTTATGGCCACCAAGTCCATGAGCCACCGCTGGTGGCGCACATCATAGGCAC
AATGAACCAGTGGTGGGTGTCCATGGACACCATGGGGCGCAGCCTGTTGGAACTGCG
GCCCCTTTACCTTTACCGGTAG
>LsfLEA2-57
ATGGGACGTATCAAATTTTCTCGCAGAGGCCTGAAAATCTGTTGTGGCGTCACTGCAAT
TCTCTTTATCATCATTCTAGTGGTTGTGATTGTCTTGTCCCTCACCATCTTTAAGCCTAAA
CAACCCAAAATCACCACACAGTCTGTTACCCTCGAGCACGCTAAATTAGTAATCTTTCC
AGTATTTAAGCTAAATGTGACACTGGGGATAGCATTGACGATTGATAATCGCAATTATGG
AGGGTTCAAGTACGAGAACAGCACATCTTATGTTAGTTATCGTGGGACAGTTGTGGCT
GAAGCTCCAATCGAACACGACAGGATTCCTGCTCGTGGAAAGCTTAATGTAAGCACAA
GTGTGGAAATTGAGGGAGATAAGTTGACGCTAGATCCTCAATTTAAGCAAGACTGTGA
TAGTGGGATCGTGAATTTTACTTCATCGACCATTTTGCATGGCAAAGTGATCTTACTCAA
TTTTTGGAAGAAGAAAGCCACCACGTATAGTGCCTGTAACATCTCTGTCTATGTCATTG
CTCAAGCCGCTGATTCAGTCTGCCATTCTAGAGTCAAGTTTTGA
>LsfLEA2-4
ATGTGTGATTCCAAAAACTTCTATGTATGGCTTCTCCAAGTTTTTGCCCTCTTGGGCCTC
CTTGCCTTCCTTCTATGGCTAACCTTGCGCCCCAAAAACCCCACCTACACCATTGTGGA
CTTCTCTGTCCCAACCTCAAATCAGAGCAATAACACAAGCAGTGAGAATGGCGCCATT
TCTTACGTCCTCGAAATCCAAAACCCAAACAAGGACTCCGGTATTTACTACAATGACAT
CCTCCTAACGTTTTATTATGGGCAGGACAGTGTCGGAGAGAAAACTATACCTTCATTCT
ATCAAGGAAAGCACAAGACTCGTCAACTAAGTGACCATTTTGATGCCAATGCGCGGGT
GTGGAAAGCTCTTCGCAGCACAATATTGAATGCAACAGCTGAATTGAAGGTAGGATTA
GTAACTAAGATTCGATATAAAATGTGGGGTCGAAAGAGTAAGCATCATGGGATGAACTT
GCAAGGTCAAATACGGATTGGTTCCGATGGGAAGATTTCGGGTAAGAATAAGAAGATT
AAGCTAAAGCATACTTCCAAGAGATGGAGATCAAGGTATGCTTGA
>LsfLEA2-20
ATGCATACCGGCGACGGATTACCGGTTCAAACCCCACCCAAAACCCGACCCATAAAGC
GACACCACACAACCCGCTATTACGTGCACCGGGTGCACGAAAGCTTAACCACCCGGGT

CTCCAAGATCTTATGTTCCATCTTCCTGGGCCTTCTTCTCGTCATTGGCGTCGTTGCATT
CATCGTCTGGCTTAGTTTACGCCCGCACCGACCCAGATTCCACATCCGTGATTTTTCAG
TACCCGGGTTGAACCAAGTGAACGGCATCGACAATGCCGTGATAACGTTCAACGTGAC
AGCGCGTAACGCAAACCAAAATATCGGGATATATTATGACTCCATGGTAGGGTCGGTTT
ACTTTGAGGATCAACGGATTGGATCAACTCCATTGCTGTTTCCTTTCTATCAGGAGCCG
AAGAACACGACGGTGGTTTATGGTACTTTCAGTGGGGCCACGTTGACGGTTGATAGCC
AACGTTGGACGGATTTTAAGAATGCTCTGGCGAAAGGGTCGGTTATTTTTCGGCTTGA
ATTGACTTCGACGATCAGATTTAAGATTTCTCGATGGAAGAGTAAGCGGCATCATGCTC
ATGCCGATTGCGCAATTGGAGTGGGCCAAGATGGGTTGATTTTGGCAGTTTCGAAAGA
TAAGAGATGCCCCACTTATTTCACCTGA
>LsfLEA2-39
ATGGCAGATCACCAGAAAATACATCCGGTGGTGGACGTGGAAGCACCGCCAACGACT
CCTTTAGTGCCTCACGGCTCCTTCAGATCAGAAAACGGCGATCCGGTTCAGCAGAATC
CTCCTAAACATGCTACTGTCATTCCAGTGATCCACTCAATACCACCAAAGACGGAGAG
AAGCCGTTGTTGTTGCAAGTGCATATGTTGGACAATTAGCCTCCTTGTCATTATTCTGAT
TATCATCGGAGCCACAGCAGGCATTCTTTACGTCATCTTCCGACCAAAACTTCCTCAAT
ACTCAATCAATAATCTGAAGATCAGCGATTTCAGACTCAATTATGACATGACACTATATG
CTAAGTTCGACGTGAAGATCACAGCCCAGAACCCAAACAAGAAAATTGGAATTTACTA
TGAGAAAGGGGGCCGATTGAGCGTGTGGTACACAGGCACAAGGCTTTGTGAAGGGTC
ACTTCCAAAATTCTACCAAGGTCACCACAACACAACAGTACTCGACGTGTCCTTAACC
GGCCAGACGCAGTATGGAAGCACGTTGATGACGGCATTGCAGCAGCAACAACAGACG
GGACGCATCCCTTTGGATCTTAAGGTTGATGTGCCTGTGGCTATCAAACTTGGCAACCT
GAAGCTCAAGAAAGTAAGGTTCTTGGTGGGATGCAAGTTGGCCGTCGATAGCTTAACA
GCCAATAATAAGATTAGCATTACAACCAGTGATTGCCGCTTTAGATTGAAGCTTTAA
>LsfLEA2-55
ATGGCGTCATCTGATAACCCAGATATAGTTGAAAGGGGTGATAAGGAGAAAGATCACA
AGGAAGATGATAAAGATGAAGGAAAGGGTGGGTTTATTGAAAAGGTCAAGGATTTCAT
TCAAGACGTAGGTGAAAAGATTGAGGAAGCAATTGGATTTGGGAAGCCAACTGCAGA
TGTTGCTGCCATTCACATTCCTTCAATTAATCTTGAAAAGGCGGAGATTGTCGTTGATGT
TCTTGTAAAGAACCCAAATCCCATTCCGATTCCTCTAATTGACATAAACTACTTAATTGA
GAGTGATGGGAGGAAATTAGTTTCTGGGTTGATCCCTGACACTGGAACTATCCATGCAC
ATGGTTCAGAGACCGTCAAGATACCTCTTACTGTAATATATGATGACATTAAAAGCACAT
ATGATGATATAAAGCCTGGAAGCATTATTCCTTACAAGATCAAGGTTGAGCTCATTGTAG
ATGTGCCTGTTTTTGGAAGGTTAACTCTACCACTTGAGAAAACTGGCGAGATCCCCATA
CCATACAAACCTGATATTGATATTGAGAAGATACAGTTCAAGAGATTCTCTTTTGAAGA
AACTGTTGCAGTTCTTCATTTGAAACTGGAAAATAAGAATGATTTCGACTTGGGCATTA
ATCGCTGGACTATGAGGTTTGGCTAGCTGATGTGAGCATTGGAGCTGCAGAACTCTCTG
AATCTACCAAACTTGATAAAAATGGAATCGGTCACATTAATATTCCCATTACCTTCAAGC
CTAAGGACTTTGGCTCTGCACTTTGGGATATGATTAGAGGCAAAGGTACTGGTTACACC
ATGAAAGGAAACATAGATGTGGACACACCCTTTGGAGCAATGAAGTTACCCATCAGCA
AGGAGGGTGGTACTACCCGCCTCAAGAAGAACAAAGGAGAGGGAGATGACGATGAC
AACGTTGACGTCGATGACGATGAGGTATATTTACTGTGCTGTTTTCTTACTCACTTGTAA
TCTTTCATTCTTTTCATCTAGTGTTTTGTTTTATGCTCATGTCCTTGTTTTTGTTGTATTTG
GTAAATGCTCATCTTGTGCTGTTTTCTTACTAATTACTAAATTGTAATTAGTGGTAGTCCA

CTACTAGGTTTAATCCGTTTTGTTGTATTTGGTAAATGATCACTGAGGTATAGAATTCCA
ATTATCCTGATTTACTTTTACTACTAGAACTATGAAAGGGCACTAACATAGATAAATCCT
CCTTTGCTTGCTGCCTTGCTGGGTACAGTTCAACTGCTGTGTATTTAACTTTGCCTTTTC
TATACACTAGTTGATGCACACATGCGTTGCACGTGGATATTTTAAAGTTATATATATATAC
ACCCTTACTAAAATAAATACACCCTTAGTAGGATAGGATCTCAAATCACATATACCCTAC
CAGCGCAAAAGTTTTTTTTTTGTCTCGAGTCCTAACCACACTGAACAGGCTGATGTAG
TATCTCTAAGTTGTTAAGAATCTAATCTCAATATGAACATAAAAGTTGGACATTGTTGAA
ATAGATGAGAAATTATAAACTTCTCAAAAAAAATTATCAATATATGTTGATATTGGTGTA
AAATTTTTGTCAAAACTAATCCAAATCTTTGTTATTAAGTGAATTGAATTTATCATTAAA
GCAATTAGTAGTTCAAAAAAAGAATAGTTACAATGACTACGTTAAGGTCACAAGAAGT
CAGGTATATATATTTGTAAATCATCAAGATAGTAAATCAAAAGGTTTACAATCAATCTGA
TTTTTCCAAAAGCATGCATTAGGTGCTGTAGAGTAG
>LsfLEA2-14
ATGGCCGAGAAGAACCAGCAGGTGTACCCTCTAGCACTGTCAACCGGCGGACAACCC
AGAAGTGCCGACGAAGAATCAGCAACTTTGCATTCCAAGGAGTTCCGCCGCAAGCGA
CAGATAAAAATCGCCGCATATGTTGCTGCCTTCGTCGTGTTCCAAACCATAATCATTGCC
ATTTTTGCGCTTACTGTAATGAAGTATAGAACTCCAAAATTCAGGGTGGGCAAGGTCAC
GATGGACACCCTTGTTTCCACCGCCGCGCCTTCGCCTTCCTTTAACACGAGGTTCGTTG
CCCAAGTTACTGTGAAGAATACCAATTTTGGCCCATACAAGTTCGACAACACCACGGC
CGTATTCACCTATGGGGGCATGACAGTAGGCCAGGTAGCTATTCCCAAAGGTAAGGCTG
GGCTTCGTTCGACCAAAAAAATGGAGGTGACAGTGGAGCTGAATTCTGATAGCTTGAC
GAGCATGTCGAGCCTTGGAAACGATATCAGTTCAGGGGTATTGAAACTGAGCAGTCAG
GCAAAGTTGAATGGGAAAGTGGAATTGATGTTTGTGATGAAGAAGAAGAAATCTGCCG
AGATGAACTGCACCTTGACATTCGACTTGTCAGCAAAGGCACTCCAAGCCTTGGATTG
CAATTGA
>LsfLEA2-27
ATGGCCGAGAAGAACCAGCAGGTGTACCCTCTAGCACTGTCAACCGGCGGACAACCC
AGAAGTGCCGACGAAGAATCAGCAACTTTGCATTCCAAGGAGTTCCGCCGCAAGCGA
CAGATAAAAATCGCCGCATATGTTGCTGCCTTCGTCGTGTTCCAAACCATAATCATTGCC
ATTTTTGCGCTTACTGTAATGAAGTATAGAACTCCAAAATTCAGGGTGGGCAAGGTCAC
GGTGGACACCCTTGTTTCCACCGCCGCGCCTTCGCCTTCCTTTAACACGAGGTTCGTTG
CCCAAGTTACTGTGAAGAATACCAATTTTGGCCCATACAGGTTCGACAACACCACGGC
CGTATTCACCTATGGGGGCATGACAGTAGGCCAGGTAGTTATTCCTAAAGGTAAGGCTG
GGCTTCGTTCGACCAAAAAAATGGATGTGACAGTGGAGCTGAATTCTGATAGCTTGAC
GAGCATGTCGAGCCTTGGAAACGATATCAGTTCAGGGGTATTGAAACTGAGCAGTCAG
GCAAAGTTGAATGGGAAAGTGGAATTGATGTTTGTGATGAAGAAGAAGAAATCTGCCG
AGATGAACTGCACCTTGACATTCGACTTGTCAGCAAAGGCACTCCAAGCCTTGGAGTG
CAATTGA
>LsfSMP-1
ATATCAATTTTGACCCAACCATTCCGAAAATCCGACCCACAAAACCACTTTCACTCGAC
ATTTGACAAGAATGAGCCAAGAACAGCCACAAAGGCCTCGAGCCGACCGGGAACCGG
TGAAATATGGCGACGTTTTTCCCCTCTCCGGCGATCTTGCCTCCAAACCCGTAGCACCA
CAGAACGCAGCTATGATGCAGACAGCTGAAACTATTGTTTGGGGACAGACTCAGAAG
GGTGGACCAGCTGCCGCTATGCAATCTGCAGCAACCCTCAACGAGAGGGCTGGTCTTG

TTGATCATCGTGATCTGACCGGCATCACTGGAGACCAAGGCGTGACTGTTGCGGAGAC
CAATATCCCGGGAAGCCGTATAGTAACTGAATCAATCGCAGGACAGGTTCTCCTTCTAT
ATAATGGAAAATATTAAAGAGTAAAGAGTAATCTTTTTCGTCGAATATGCTTGCCCAATT
CATGCATGTGGTGGTGGTTGATGTGGCATAATGTGCCATAGACATGGTTTGTCACATCA
AACACGTATAAATTTTTTTCCGACTATATTTGCTCAAGTAATGTTCTTCTATAGTCTAAG
GTCAAATTAGATGATACAGACGTTAAGATGTCGGATTCATAGAATCCTCTGATATTTTTA
ATTTTTGATTTTTTTCTAAAGAAATACAAACATGCATACTTGCATGTTTTATTTGTTTGAT
AATCTTGTAAATGCATAGGTTATGCATGCATTGTCTGCTTGATAACCTTCTACTTTCATAG
ATTATGCATAGAAGCTTTATTTTTCTGATAATTAGTCTTCTATTTCCACCTATAGGTCGTT
GGACAGTATGTTGAACCCTCGCCAATGGTACAAACGACTCCAGGGCAAGTGTTACTAC
AGAGTGCGATAACAATAGGTGAAGCACTCGAAGCAACAGCACGAACAGTCGGCGACA
AGCCAGTGGACCAAAGTGATGCGGCCGCAATTCAAGCAGCAGAGGTGAGAGCAACTG
GTTCTACCGTCATAAACCCAGGTGGGTTGGCAGCTTCAGCGCAGTCGGCTGCGTCTTT
CAATGCCGCAGTGGATCAGGACGAGGACAGGATTAAGCTGAAAGATATTTTGGCGGTA
ATGCCATCATCTATATACATGTTTGTTCCAAGTGTTTTAACAATTAATATATACTCCATGTT
ACTCCCATGTCCTGTATTGATTCTTCTGATTTCTATTATGGGCTGTTCTAAAATTAATTTT
CAATAAAAAGGTTAATGAGTTCGTCTTAACTGATTTTTCAAAATACTCTCACGCATAATT
TTTTTTTTTTTTTTTTTTTAGTGTACAAATAGAGCAAAAACTTAATGGGTAACTCAATTG
GTGAGAGATCTTGTCTCAGGAAGTGGAGGAAGTGGAGTTTGGCTCTTGCTATTCTCCT
CTTGGAGTCACCAAAACAAAGTTACCATAGATCTCAAAGGCTTTGGACCTTTGGTCTTA
CCTTGTCCATGTGGATTGTAAAGTTTTACATGGATTCGGGGCAATCGAAAGGTTTCATC
ATCATAAAAAATTAAAAAATAAAAAAATAAAATTGAGGGCTAAATGTACGAATAGAGA
GTGCAAAATTAGAATATTAAAATATTACATATTTATGAGTGTAAAAGCATTTAAAATAGTT
TTTTCAAAAGAGATTCTCGATATGGAACTGAGGGTTTACTTGATTGTTTCAAGGATTGG
GAGGTGTGATATATGGGTTGTTATGCAGGATGCGACGGGGAAGTTGCCGGCGGATAAG
GCGGCGACGCGGGAAGATGCTGAAGGGGTGATGAGTGCAGAGATGCGTCATAATCCG
AACTTGGCAACGCACCCGGGTGGAGTAGCGGCGTCTGTGGCTGCAGCTGCTAGGCTC
AACGAGCAACTTGACATATGATAGAACTGAAGTAGTGCCCTGAGTGATGTGTGTAGGA
GTAGCTTTTGTGTAGTCAAATGCAAATGATCGATCCTAAAACTATTGTGTGTAGTACACT
CCTAGTAGCTGTGTGTAACATAGTATTGTGTAGTCTCAAGCTTTCTTTTGGTATTTTAGC
AATTATATAATCAGGTAATATTTTAGTTTCAAGCTTT
>LsfLEA2-50
ATGGCCGAGAAGAACCAGCAGGTGTATCCTCTAGCACCCGCAGCCAGACAACCCAGA
AGTGACGATGAAGAAACAGCAACTTTGCAATCCAAGGAGTTCCGCCGCAAGCGACAG
ATAAAAATCGCCGCATATGTTGCTGCCTTTGTTGTGTTCCAAACCATAATCATTGCGATT
TTCGCAGTCACTGTAATGAAGTATAGAACTCCAAAATTCAGGGTGGGCAAGGTCACAG
TGGACACTCTTGCCTCCAGTGCCGCACCTTCGCCTTCCTTTAACACGAGGTTCGTGGCC
CAAGTTACTGTGAAGAATACCAATTTTGGCCCATACAAATTCGACAACACCACGGCCG
TATTCACCTATGGGGGCATGACAGTAGGCCAGGTAGTTATTCCAAAAGGTAAGGCTGG
GCTTCGTTCGACCAAAAAAATGGATGTGACAGTGGAGCTGAATTCTGATGGGCTGTCG
AGCACATCGGCCCTTGGCAGCGATATCAGTTCAGGGGTATTGAAGTTGAGCAGCCAGG
CCAAGCTGAATGGGAAAGTGGAATTGATGTTTGTGATGAAGAAGAAATCTGCTGA
AATGAACTGCACCTTGACATTCGACTTGTCAGCAAAGGCGCTTCAGGCCTTGGAGTGC
AACTGA

>LsfLEA2-30
ATGGCCGAGAAGAACCAGCAGGTGTACCCTCTAGCACTGTCAACCGGCGGACAACCC
AGAAGTGCTGACGAAGAATCAGCAACTTTGCAATCCAAGGAGTTCCGCCGCAAGCGA
CAGATAAAAATCGCCGCATATGTTGCTGCCTTTGTTGTGTTCCAAACCATAATCATTGCG
ATTTTCGCAGTCACTGTAATGAAGTATAGAACTCCAAAATTCAGGGTGGGCAAGGTCA
CAGTGGACACTCTTGTCTCCACCGCCGCGCCTTCGCCTTCCTTTAACACGAGGTTCATT
GCCCAAGTTACTGTGAAGAATACCAATTTTGGCCCATACAAATTCGACAACACCACGG
CCGTATTCACCTATGGGGGCATGACAGTAGGCCAGGTAGTTATTCCTAAAGGTAAGGCT
GGGCTTCGTTCGACCAAAAAAATGGATGTGACAGTGGAGCTGAATTCTGATGGGCTGA
TGGGCACATCGGCCCTTGGCAGCGATATCAGTTCAGGGGTATTGAAGTTGAGCAGCCA
GGCCAAGCTGAATGGGAAAGTGGAATTGATGTTTGTGATGAAGAAGAAGAAATCTGC
CGAGATGAACTGCACCTTGACATTCGACTTGTCAGCAAAGGCACTCCAAGCCTTGGAG
TGCAATTGA
>LsfLEA3-1
ATAAAATTATACCATCTTCTAAGAGAGGGAGCCAGAGAGAAGAAGTAACTTTCGTTGC
TGTAGAATTTCTCTTTTCGAGAATTCATTCGAAGAAGAAGAGCTTTCTGCAGTAGCGAA
TTATATCTCTTTGTTTTTTGTCTTTGAGAATTCACAATCAGATGGCTCGCTCTCTGTCTAA
CGCTAAGTTTCTCGCTGCTTCCGTCGTAGATAGCCTTTCCCTCGCTATATGCCGGTGAGT
GTTTATGAATGATTTAATATGCTTTTTAGATTTGTATGTACTCCGTATAAGCTGTGTTTTCT
GGGGCAACCGTGTCATGGGACAGAGCCTTGGCTGGATGAGCTAGACTCCTATTGAGTG
CAGTTTAGTTGGGTTGTTTGTGTTTCATCTAACATCCGCCGGGTGTGTGTCTCTGTGTG
ACTAAATCAGAGGGTAAATGGTGAGGGCTTCCCATGGCATGATATTGAGTCATGACCCT
TCAATCTTAGCCGCTGATTGTATTTGCCCTTATCTACTTTTTCTCCTTTGGTTAATTTATAT
TGGGTAAATTATAAGAAGCCCTGAAAAAATGGTTGGAATGTTTTAACCCGTTTAACAAT
TAGTGTCACCATTAATTATTGATTATGGATTTAAACAAAAAAAGTCATGAGATCACTTTT
AATTCCATCCTTAATGTAATCTTAGAGAATGACACTATATTAATTTTTTTCAATACAAAAG
GATGATTAAAATTAATAACAAACAACCTACGACAGTATCCTAAGCTTATTTTTACCACAA
GTTAAAGATTAGATTAGTTTCACATGATGCACGAAAATCTGAAACCCTAACCTTCGCTA
GGCTTATTTCATCACAGCTTTGACTATAGATTAGATTAGTTTAATATGATGCATCTTCACT
TCACCAAAAGTTTCAACAATATAATCGAATTAACGCTTACGGCGCACGATAACGTGACG
CTTCAAAAGTCTAGATAATGATTATTATTAAGCACTATCTTAAATTCTTAATGATGTTTAT
TATTTATCTATGTGGTGAACAGGCGAGGTTATGCGGCGGCATCACAGGGCATGGTGTCG
GCTAGCGTTTCTAAGGGAGGGTCCAGGACTGGTATCGTGGGGAGAGTGGAAGAAAGG
GCCGTGATTAAAGAAGATTCTTCATGGGGCCCAGATCCAGTTACGGGATACTACAGGCC
CGTGAATCGTGGGGCTGAGATTGATGTGGCTGAGCTTCGAGACACGTTGTTGAACAAC
AAGGTGAGGAGGGCACATTAGAACAACGGTGGGGAAGAGGTGTGTGGGATTGAAGG
GGAGTTAAATTTGGAAGGATATGGGAAGAAGGGCCACGTGGTTAGCCTTCGTGCGCGC
ATTATTGTGGAATCTTATGCAGTAGAGGTGGTTGACCTATTTTGTTTTGTATCAATTTCTA
ATGGCTGTTTGTTGGCTTGTAATGACCAGCCTGGCAACATTTAATATAATAGAAAGAGT
AATTAATTCATAATTATAT
>LsfDHN-2
ATGGCAGATTTACGAGATGAATATGGCAGCCCAATTCAACTGACCGACGAACGTGGTA
ATCCGGTTCAGCTGACCGACGAATATGGCAATCCGATGCATCTTTCCGGCGTTGCAACC
ACGCACGGCACAACGGCGACCACTGGAATGCACGGTGGTGCACCGGAGATTCCAATT

GGGTTGGAAACCGTCAAGCATGGTGGTGCCCCGGAGGCTGGGGCCGGTCATGGAGGT
GGGCAGCGGGAGCTGCAGCAGGTTCAGCGCTCCAGCAGTTCAAGCTCTAGCTCGGTG
AGATAAAGGATACACTGATATAGTTATGACTTATGAGCAACGCCTCCTTTTTCTGCCTTT
TTCTTTTTTTTAAATTTATTATTATTATTGTTATTTTTTTGGGTTAAGTATTTTTTTTAAAAA
TAAAAAATTTAAAGGATTGAACGGGTGATCAGAGTGATTTTCCTATCTATCTGGACCAA
GATAGCCCATCGGCGTGACTAAAATAGCATATTTTTTATTCACCCACCCACTAATAGCCG
TGGGTTCTACCGTTCTTCTTTTAGGAAAATGCTACTTCATATTTCAAATTCACAAATAAT
AAAATCCCTACGATGTGACATAAGTCTATATGCTATCATAAATTAATTTCTTAAACGAGA
AATTGTGGGACTCATCCAAATTAATGCACGGGTGAATAAATTAGGTGAATAGTATGGAT
TAAATAGCATTGTCCAAAATTTAATTTTTGATAACATATAGACTTTACGGCACATCAAAT
GGGATCTGATTTTCGGTAAATAATATGGCTTAAACACTTTTTTTTAAAAAATTTTAAATA
CTAATATATTAAGCTTATTTACTCTACAATTACTGTTATTTTCATGCAATTCGCTGTATTAA
ATATAATATAATTTTTTCATTTGTTTAGTCTGAGGATGATGGGCAAGGTGGGAGGAGGAA
GAAGAAGAAGGGACTGAAGGAGAAAATAAAGGAGAAGTTGACTGGTGGAAAGAGCA
AGGATGAGCGTGAGCAGACCACGGGTTTCACCGCTAGCACCACCACCACCACCACTA
GCACCACCACTCCAGGGCTGCACCACCAGGAACATGAGAAGAAAAGCATTGTGGAGA
AGATAAAGGAGAAATTACCAGGCCACCACAAACATTGA
>LsfDHN-3
ATGGCAAATTTACGAGATGAATATGGCAACCCAATTCAACTGACCGACGAACGCGGTA
ATCCGGTTCAGCTGACCGACGAATATGGCAATCCGATGCATCTTTCCGGCGTTGCAACC
ACGCACGGCACAACGGCGACCACGGGAATGCACGGTGGTGCACGGGAGACTCGTACT
GGGTTGGAAACCGTAAAGCATGGTGGTGCACCGGAGGCTGGGGCCGGTCATGGAGGT
GGGCAGCGGGAGCAGCAGCGGGTTCAGCGCTCCAGCAGTTCGAGCTCTAGCTCGGTG
AGATAAAAGATACATTGATATAGTTATGACTTATGAGCAACGCCTCCTTTTTTTTCTTTTT
TCTTTTTTATTATTATTATTTTTTGGGTTAAGAAGGTTTTTTTTTTTTTTTAAAATAAAAA
ATTTAAAGGATTGGACGGGTGATCTGAATGATTTTCCTATCTATCTGGACTAAGATAGCC
AATCGGCGTGACTAAAATAGCATCCTTTTTTATTCACCCTATTGCGATGGAACACAGAA
GGAGAGAAAGGAAAAAGAGGGTTAAAACCCTTGCTATTGATTGTGTTTATTTCATGTAC
ATGAGGGACCTATATATAGAGTTTAGGCTAGGAATAATCCTAGGTATAGCTATACAATAC
AGGGGCCTACGCTATACTTTATACAGTTACAATACACATACACGGTAACACCCCCCCTC
AAACTCAAGTAGGGTTTGTAGAGACCAACTTGAGTTTGGAAACTAAATCGAGATGGCG
TCCTGGAGGATGAGATTTGGTGAAGATGTCTCGCAGTCGATCTGTGGAAGAGACGGAG
AGGAGACGACAAGGTACACTGGTAATGTGTCAATTTAATGAAATGACAATCAATTTCA
ATATGTTTAGTGCGTTCGTGAAATACATCATTATGAGCAATCAAGAAGAGCACTACGATT
GTCACAATAAAGAACGAAGAGCTGTCGGAGAGGAACTCCCATAGTAGTAAGAAGCCA
ACGAAGCCAAAGAAGTTCTTGGGTAGTATCGGCGAGAGCACGATATTCAGCCTCAGTG
CTAGAGCGGGCGGTGACAGTTTGCTTCTTGCTACGCCAGGAAATAAGGGAGGTGCCAA
GAAAGAAACAGTAGCCAGTGGTAGAACGGCGGTCAGTCGGATCACCGGCCCAATCAG
CGTCGGAGTAGGCACGCAACTCAAGGGAAGAGGCGGCAGAAAAATGAAGACCATGAT
ATAGGGTACCTTTAATATATCGAAGGATCCGGAGAACAGCTGCATAATGAGAAGAGCGA
GGAGCAGACATAAACTGACTGACAAGATGTACTGCATAGGCAAGATCGGGCCGAGTAA
CAGTGAGATAAACAAGGCTGCCAACTAACTGTCGGTACAAGGTAGCATCATGGAGAGG
TGTACCATCTAGAGGAGTGAGGCGAACATTAGGCTCGAGAGGAGTTGAAGCGGTCTTG
CTATCAAGAAGACTAAAGACAAGTGAGAGATCGAAACATACTTGGCCTGAGAAAGATA

GTATCCCCGAGAATCAGAAGAAATCTCAAGACCCAAAAAATAGCTAAGGGAACCAAG
ATCCTTCATCTCAAAGTGTTGGCTAAGAAACTGTTTGAGTAGAAATGCCAAGAGAATC
ATCACCGTGATAATCATATCATCAACATAGAGAAGCAAAAGAATGGTGCCATGATCACC
GACGAACAAAAAGAAATGAGTCATAGGGACTAGATGTAAAGCCGAAATCTCCAATAAC
AGAATCGAATTTGGCAAACCAGCTCGAGGAGCTTGCTTGAGTCCATACAAGCGCGCGA
CGGAGGTGACAGACCTGGTTAGGTAAATGATCATACCAGTGAGGTGGTTGCATGTAGA
CTTCTTCATGAAGATCACCATTTAGAAATGCATTCTTAACATCCATCCAAATAATCCCCT
ATGGCGGACAAATGCCACAGCCTAGGAGACTCCGGACGTAAGTAAGACGGCGACAGG
AGCAAAGGTCTCTTCATAGTCAATTCCATACTCGAGTAAAGCCTTTGGCAACGAGGCG
AGCCTTATAGCGCTCAATAGCTCCATCGAGCGAGTCTTGATCTTGTAAACCCATTTACAT
CCAATGGCATGTTTGCCAGGGAAGATCAACCAAGTCCCAAGTATGAGTTTTGTGTAAA
GCCCGTAATTCAAGATCCATTGCTTCTTCGCCAAAGAGGGTTAGTACAAGCCTCGCGAT
AAGATTGAGGCTCATGTTGAGAAAGAATAGTGGAGAAACAATGAAAGTCACGAAGAT
GGACTGGCAATTCTCTTACCCGAGTAGAACGGCGAAGAGCTGGAGGAGGAGTGGGAA
CGAGATTGTCGAGGATCGCCAAAGGGACCGCACCGCAGAGGGGCCGTAGTTGGTGTA
TCTCGAACTCGTGTCAACATCATCGTGAAATAAAGAAATAGAGGGTTAGTGAATAAAG
AAAGGGTAGATGAGGAAGGGAGATGAAATTTGGAGAGAGAGGAGAACAGTTTGTGTT
CCCAAAAGACCACATGACGGGAAATACGAAGACGTTTGGAAATAGGATCCCAACACC
TATAGCCTTTGTGTTCTATCCCATAACCCAAGAAGCAGCAGAGACGGGCACGAGGTTG
AAGCTTGGTACGTTCATGTGGTTGAAGTAACACAAAACAGGCACAGCCAAAAACACG
GAGAGTACTGTAGTGAGGAGGTGTACCATAAAGCCGTTCATGGGGAGATTGATTATTAA
TGACAGGAGAGGGAATGTGGTTGATGGTGAAAACGGCGGTGAGAGCAGCTTCTCCCC
AAAAATGCTCAGGACAGGAGGAAGAGAGAAGAAGAGAACGAACAAAGGTCCAAAAT
GTGACGATGTTTTGCGTTCGGGCGACCATTTTGCCGAGAAGTGCCCGGGCAAGAACGA
TGAGGAAGGGTGCCTTGTTGTTGAAGAAATTGAGGAAGTCGATGTCGCGGTACTCCAT
AGCATTATCTGCACTAAAGATTTTAATAGGACGCGAAAACCGAGTGGTAATCATGCGCA
AAGTTATAGTAAATCTGCTTTGATTCAGCGATGTCTTGCATAAGATAAATCCAAGTAAAG
CGAGAATAATCATCAATGAAGATAACAAAATACTTGGATCCACCCATGGTGGTGGTAGG
AGAAGGACCCCAAATATCGAATGAACTAAGTCAAATGGGGCATTAGAACAGAATCACT
ATTTGAAAAAGGTAAAGGTGTTGTTTATGAAATGACAAGATAAACAATCATCATGTTTA
TTTATTACAGGCCCAAAATACCACTAGAAGCCAATTTTTGTAAACGATCAAGGAAACAT
GTCCTAAGCGAGCATGCCAAAGAGTTAAAGAGGAGGAGTTGAGGCAAAGAATGAGTG
AGCGGGAGAGGTAAGGTGGGATGGAGTGTGGAAGTTGATCAATTCAAATAAACGTCC
AACTCTACGGCCGGTCCCAAGCGTCGTCTCCGTCTTTGGATCCTGCATAATACACCGAA
GAGAAGAGAAGGTAACATTAAGACCAAGTTCACAAAGTTGTCCCACGAAATAAGATTT
AAAGTGAGTTGAGGAATGAAATAAGTATCGTGGAGAGACACTTTGGAGTTAGAGATTT
GTCCAATGTGATTAATATGCATGTGAGAGCCATCAAGATATGAATAGGAGGAAGATGAG
AAGTATGAACTTGAGTAGTGAAAAAGAGTGATCGATGTCATATGATTACAACAAGCCG
AAGTCAAAATACCAAGAGAAGTACAGTGTGACTGGAGAGAAGCGAGGGAGGAGGGT
TACAAGTAGAGAGGACCTGTTTGAGAAGAGCGATGAGATCGGCATTAGAGAGAGATG
AAGTGGGAGAGGTAGGTGGAGAGATGTCCTCGATGTGGCAGCAGCAGAGTAGCAAAG
AGAGTGGAACGATGGTGAGGCTTGAACTGTTAGACCTAGATGAATTTCTCTGGAATGA
TCATGTCGAGGTGTGGGTTTGATTGCGTGGACGATCATTTAAGAAATGACTGGACCGC
GCTTGTAATAAAGGCTTGCAGACTCGAATAGGACAAATGAAGCAGTATGGTTCGGCGC

ATGACAAAACTTGCAACCCTGATAGAGATGGACGCCTGAGAAGGAGGAGTAGCCATA
GCAAGTGTGGGCAGTCTTGAGAAGGGAGTTGCAAGAGCAGATTCAAGAGAAGGAGG
AAGGCGAGTACAAATGTCGAGTCTCATATGCAACAAGTTCGTAAACAAAGACTTTCCA
AGGTCGGTAATGGAGTGCGATGAAGCAAAATGGCACGTGTAGGTTCGAATACATCATC
CAAGGCCATCAAAAATGGATTAGACGCCGATTTCAGAATCGAGAAATTTCGCGCAGAT
CCCGAGAACAGTCCAAGAGGTTCTGGCGCCAATTGATCCCAAAAGCGCCTCGTGATCC
CGGAGTGGAAAATGTCAATTGCTTCTCCGGTGTTCTTGACGATGCGGTGAAGTTGATCT
AAAAGTTGGTATCGATGAGAGAGACCAAAGATGGTGTAGCGGTCGGAAAAATCCTAA
AGACTTTGGCGGTTTCAAAACGGCCAAACCGCATACCAATGGATGGAATAGAGTCAAA
AGCGAGATGATCTTTTGATTCTTACTGTCCCACTCTTCAAGACGATCCACAAATTTTTCT
TCAAGTTCTTCTGTCCCTTTCTTTGGTTCAGTGACATCACCAGTGACAATTCGCCAGAG
TTTGTGACCACGAAGGTAGCCAGACATGGCTTGAGCCCAGAGAACATAATTCTGGCCA
TTTAAGACCAGAGGGATGTTGATAGCAAGCTCTGGGTTACTCATAACTGCACACTCGG
AGTAGAATTAGCCAAAAAGGAGCTCAAAAATGAGATCAGAGGGCTGAAAATACCTAA
GAATGACTGTTTGTTTGCTGAAAATGGTCCAGAAATGGAGAAAATGAAGAAAAATCAG
CAAACAGATGCAGTATGGCTGACGTGGCAAAGAGATGGCGATGTGGCGATGGAAGTT
GCCGACGCGGCGCCGACGTGGCGATGGTGTGCCGACGTGGCGATGGCAGCGCGGCGT
GACGTGGCGCCGGCGATGGTGCAGTCTTGGCGTGCCGGCGTGGTGGTGGCCGATGGC
GATGGTCTGGCCGACGTGGCGTTGACGCCGCTGCTAGCGACGGGCCGATGACGTGGC
GATGGACGTGGCGCCGACGTGGCGATGGTGGTGCCGACGCGTGGCCGACGTGGCAGA
TGGTGCGGCGGCGTGGCGATGACGTGGCCGAGGTGGCGCGACGTGGCGATGGTGGTT
GACGGCGTGGGACGATGGCAATGGGTGCCGGGGTGGCGCGGCGATGACGATGGCGTG
GGCTGGCGGCCGCTGACGCGATGGCGAATCTGGCGACGTGGGTGATGGCGTGGGCGT
GGTGGTCGCCGACGTGTCCGCCGACGTGGCGATGGTGTGCCGACGCGGCGATGGTGC
GCTGCGACGCGTGGTGCGTGACGTGGCGGCGACGTGGCGCTTAACGTGGCGATTGTG
GTTGCCGACGCGATGGCGATGACGTGGCGCCGACGTGGCGGCGACGTGGGCTGGTGG
TTGGCCGACGTGTCGATGACGTGGCGATTACGTGGCGGCCGACGTGGGAGTTAGGTGG
CGCCGACGTGGCGCCGATTAGACGCTCCGACGTAAGCGCTGGAGGCGTTGTGAAGAT
GGCGCGTGGGTGGCGTGGCGAGCTTCGTCTTGGCGTGGATCCGACGCTACCAAAAAG
TGGCGCGCGCAGGCGCGTGGAGAGCTTTCGGTGAGTGGTTGACGAACTCTTTGTAGA
GCAGTGAGGCGAGGCAAGTGGATGTGGTGCAAAGGTTGGGTGATGTGCGAACGCACC
AAAAATGCGAAAAATAGCTTCGACTTGTCTTGGCGTGGATCCGACGCTACCAAGAATT
GGTGGCGATGTGGGGCGCGTGGGGAGCTTTTGGTGAGCTGGTTGGCGGCGTTTTGTAG
AGCAGAGAGAGAGGCGGTGGGTGTGGTGCAAAGGTTGGGTGATGTGCGAACGTACCT
GAAAAATGTGAAAAGGCTTCGGCTTGCTCGTCACCGGAAATGGGAGAGAAAAAAAAA
CTTTCAGAGGACCAAACCCCGCTCTGATACCATATTGCGATGGAACACAGAAGGAGAG
AAAGGAAAAAGAGGGTTAAAACCCTTGCTATTGATTGTGTTTATTTCATGTACATGAGG
GACCTATATATAGAGTTTAGGCTAGGAATAATCCTAGGTATAGCTATACAATACAGGGGC
CTACGCTATACTTTATACAGTTACAATACACATACACGGTAACACACCCACCCACTAATA
GCCGTGGGTTCTACCGTTTTTCTTTTAAAAAATTAGGAAAATGCTACTTCATATTTCAAA
TTCACAAATAATAAAATCCCTATGATAGTCTATATACTATCATAAATTAATTTCTTAAACG
AGAAATTGTGGGACTCGTCCAAATTAATGCACGAGTGAATAAATTAGGTGAATAATATG
GATTAAATAGCATTATCCAAAAATTAATTTTTGATAACATATAGACTTTGCGCCACATCA
AATGGGATCTGATTTTCGGTAAATAATGTGGCTCAAACACCATTTTTTTTAATACTAAT

ATATTAAGCTTATTTACTCTACAATTACTGTTATTTTCATGCAATTCGCTGTACTAAATATA
ATATAATCTTTTCATTTGTTTAGTCTGAGGATGATGGGCAAGGTGGGAGGAGGAAGAAG
AAGAAGGGACTGAAGGAGAAAATAAAGGAGAAGTTGTCTGGTGGAAAGAGCAAGGA
TGAGCGTGAGCAGACCACGGGTGTCACCGCTAGCACCACCACCACCACCACTAGCAC
CACCACTCCAGGGCTGCACCACCAGGAACATGAGAAGAAAAGCATTGTGGAGAAGAC
AAAGGAGAAATTGCCAGGCCGCCACAACCATTGA

>LsfLEA2-22

CTAAGAACATGACTCCTTTTTTATCTGTCAAGCAAAGGTAAAGAAAGACACGCACACA
CTCTCTCTCTCTCTCTCTCTCTTCGTCTCGCGGCAAGAAATAGAAGGGACCAGAGAGG
GGAGGGGGGTAGAAGAGGAGGAAAGAAAGGGAGCATTGTCCGACGCCGGAGCACAA
TAGCAGAAAATGACATCGAGAGCTAACGCGAACGGGGAGCACACAGCACGCCCACCC
ACGAACAGCCACCACCAGCACTACTATCCGTCGTCATCTTCTTCATCGTCAGCCTCCTT
CAAAGGCTGTTGCTGTTGCCTCTTCCTCCTCTTCTCCTTCCTCGCCCTCCTCGTCCTCGC
CGTGGTCCTCGTCATTGTCCTCGCCGTTAAGCCTAAGAAGCCTCAGTTCGATCTCCAAC
AAGTGGGTGTACAGTACGTCGGCATCACGGCAAATCCTTCCTCCACTTCCACCACACC
CACGACCGCATCTCTGTCATTGAATATACGGATGATGTTTACGGCGGCTAATCCGAACA
AGGTGGGGATCAAGTACGGCGAGTCCAAGTTCACGGTGATGTATAGGGGGATTCCTCT
AGGGAGGGCTTCGGTGCCGGGCTTTTACCAGCCGGCGCACAGTGTGAGACAGGTGGA
GACAACTGTGGCCGTTGATCGGGTTAACTTGCTTCAGGCTGATGCTGCTGATTTGATCA
AGGATGCTTCGCTTAATGATCGGGTCGAGCTTAGGGTTTTGGGTGAAGTTGGTGCTAAG
ATCAGAGTTCTTGACTTCACTTCCCCTGGGGTTCAGGTTAGCCGCTATTTCCCCTCTTTC
TCTTACTCGATTTTTTTTTTTTTTTTTTTTTTGGGGTTAAAATCTCTCTAGACTTTGAGGTT
AAGTTTGCTCGAGAATTTCTGGCTTAGTTATCTCTCGATCTCTCCCAGTCAGAAGGTGA
GCGTGCAGTGCTGGTCGGCTCTGTAACTGGAAAACCCCACTACCTTCTGTCGCCTTTG
CTTTTGGCTAACGCGCGCCGCTGATAATTTATTTCTTCTGCTAACGCGCGCCGCTGTGCT
GGACATTTTGCAAAGGGCGTTCTTACTCATTTTGTCGTATTTGCATTTGTCTCGTTTGTG
TTTTTCGTGATTGATCCCTCAGAACTTTTTGAATTCTTTGGATACCCTTGACCTTATGGT
TCTTATTTATCCTCCCCTTTCCCATCTGGTGTTTTTCGTGATTGATCCCTCAGAACTTTCT
AAATTCATTGGATGCCCTTATCCTTTTATTCTTGCTAATCCCTCCCTAGTCCCTGTTCCAG
CTTCAAAATCAAAAAGCATAAAACAAGAGTCACAAAACCCACTCTCTGCTTAAAGGTG
CTCCTAATTGCTTTCTTATCACGCTCTTCTTCGCACTAATTTCACCATTTCCAGGTCTTCT
ATTTCACTCGCATTAGAGGCGCGTGGGTTAACACTCTTGATAATTAAGTCCATTTGAAAT
TGTCCAACAAAGCTTTCCAGTTGTGCAATCTGCCTCCACCACCGAGGCGCCTCCTTAG
TCTGAATATTGTATGGGTCTATTGATTCTCTGTGTTGTGCTAAAGTGCACCGCCTGGATT
GGGCTCGCCACTTGTGAACTTGGATGGATATTCTCGGATTATTGCGTAGGTGCCCTTCA
GATTCGGCGCAACTCTCTGTTGGGCCCATTAATTTCCCGAATTTCGTATTTGCGCCGTTC
CTTGGCCTTTTCCATGCCTCAAGACGATGGTTCCCCTTTTCGGTGGTAAAGTAAATTAC
ATTGACAACTCTTACAGAAAAACAAAAAAAATAAATAAATAAAAATTCTTTGGGTTGG
TCTGGTGCCTGCACCGACCATGTCAGATATGACCCAGGACCCACCCCATTAACCGTAAT
TGGGGCGTCGGTTTTAGTCGCTTAGTGAGCGCGTGGCTGCTTCAGACTTTTTGTAATTT
TTGGCCTTTTGAATTCTAACATGCTCTGCACCAGCACAAATTAGTGAATTGCATTCCCA
CCGTCCCATGCTTTTATTCATTTCTATTATTATTATACTCCTATCTATTATTTTTTTTTTTTTT
CAGTTGAGTAGTAGTCTTGTGCCCTTTTACTCTCACTTTTTTTTTTTTTTTTGGGGGAGG
GGTTAAATCGAAAGAAAAAAAATTATATATATATATATATATATAAAATATCATAAAACATT

CTAACATTCTCACGAAGCATCTATTTTACACTCACCATATTTATAGCATTTTTTCCCCCTC
CCATACGGGGTCTAATGTGTGGATCACTTTTGACACTGTGTAGCAGTGAAAGATGAGC
GATTGAAATAACATTTTTCATCTAATAATGAATGAATATTTGTGGGGCCTGGGGCAGGTA
TCGGTGGATTGTGCGGTTGTGATCAGCCCAAGGAAGCAATCTCTTTCTTACAAGCAGT
GCGGGTTTGATGGGTTAAGCGTTTGATCTGTAACAACTTTTTCCCCCTCTCTCATTCTCC
TCTTTTCCTCTCTCTACAAATATATATATATATAAATGGAGAGAGAGAGAGAGAGAGAGA
GAGAGATGAAAAACCAAAAGCAGCCAAGCAGGATATGATGGAAAGGCAGGGGAAAC
CCCCTCAAGTCACATGTGGAGGTGTAGCCAAAAAGGCATATGAGGTTCTAATATTGGA
AATGTCATTTTCCACGATTTTGCAAATTTTTATTTTAATACTCGTGTATTGGTTTCTTCAC
TTTGGAGGGAAATTCCTAACTTGCCTGTAAAGTGTAGGCACTGATGTATGCTATTGTAC
GCCCATTGGCTTATACTGTCCCACTCCCCCTTTTCTGTGTTTTCTGTTAGCACTCTCTCT
ACTCGTTCTACACGTTGCAAAATTGTTGGATCGTCTAATGGTCAGACAGGGTGCCTGCA
TTGAACTCGGGTTGGTTTAACTTCTGTTCAGTCTTAATAAATCGGCGTTATGT
>LsfDHN-4
ATGGCAGATCAGCACTTTCAATGCCATGAAAAGGAACCAAGAGATCGTGGGATGTTTG
ATTTCTTGGGGAAGAAAGAGGAGAAACCTCAAACGGAAATGTCCATGACAGACTGCA
ACATGGCATGCCTGTCAGAACCCCAGGAGAAGAAAGAGGTAGAGAAGCCTACTCTTG
TGGAAGAGCTTCGCCGGTCTCATAGCAGCTCTAGCTCGGTAAGCTTTCTCCAAATTATT
CTATTTCGACACCACATCACCATAACAAAGAAATATTTGTTGAACAACCATACCATGAG
TGGAAGCTGCCACACTTCTAAACCAAATGAATCAACAAAGTGCTCGCCGTATTAGAAA
AAGTCACTGACTCACACTTGACCATACTTCTCTTTAAGACTCATGACTTTTTACCTAATG
GTAAAGAAGGCTTGCTTGATATGGTGTGGATGTGTGGTTGTTTCCAGACCAAAGATACG
CTATATCTAGATTCCAGAGTATTCAATCAAGTTATGAAAATGCAGAAACAAATGACAAA
ATTTTAGCCAACGCTGCAAGAAATATATGACATAAGAGCACAACGTTATAAATATAGAAT
TCATCAATCTTTATTGACAAACTCCTGTTCGCTTGCAACTATTCAGTCAAGTGATGAAG
AAGGAGGAGAGAAGAAAAAGAAGAAGAAGGGACTAAAGGAGAAGATTAAGGAAAA
GATGGCATGCCAGGGGGAAGAGGAAGTTACTGAGATTTCGGTTGACAAATGCGATAAC
ATAGTGGATGCAGAAACAACACACCCAGAGGGGAAAAAAGGCATTCTAGAGAAGATC
AAAGAAAAACTTCCAGGTCAGCACAAAAAGGGCGAAGAGGGTGCTACCACAATTCCA
CCCAAGTGTGCCCCGGATGGGCATTCTACTGAAGGCGAGGCGAAGGAGAAGAAAGGA
ATCTTGGAAAAGATCAAGGAGAAGCTGCCTGGGTACCACAAGAATGCGGAGGAAGAA
AAAGAGGAGGAGAATTGA
>LsfLEA2-25
ATGCATGCGAAGTCGGACTCAGAGGTTACAAGCCTCGACGCCTCATCTCCACCGCGGT
CGCCCCGCCGGCCGCTCTACTACGTGCAGAGCCCATCGCAGCACGATGTCGAGAAAAT
GTCATACGGGTCGAGCCCAATCGGGTCGCCCCACCACTACCACTGCTCGCCGATTCAC
CACTCCCGCGAGTCGTCCACCTCAAGATTCTCCGCCTCCCTCAAGAACGCCCACCACC
ACAAGCACTCCGCCCCGTGGAAGAAGATGCACCGTGTCGTCGACGTCGACGACGACG
ACGACGACGAAGAAGACGGAGACGGCGACGGTGGAGGTTCCGCCCACAAAGTGAGG
TTGTACTTGTGCTTCGTTGTCGGCTTCGTTGCGCTGTTTACGGTGTTCTCGCTCATTTTG
TGGGGCGCCAGCAAAACCTACAGGCCTAAAATCTTCGTTAAGGTGCGTTTCGCGTCTG
ACTCAGTGGTGTTTTGAGTCAACTCAGTGACTCGGTCGTCTGTTTGGGGATTATACACA
ACCTTAGCTGTATAAACGACACACACTATATGACTCTTACTTGTGGTAGTGCGAGACTG
CGAGGAGAGGATTGAATAGATTAATATTTGTCATCTGTCGTGAATCTTTGTCCCCGAGTC

AACTCAGTGAGTCAATGCATTGGCTTTTATTAATAGTCTCTGTTTGGTTGTGTGATTTGG
CAGAACATTGTGTTCAAAAACTTCAATATTCAGGCCGGTATGGACAGTACAGGGGTGC
CAACGGATATGTTGTCGTTGAATTCGACGGTCAGGATTTCATACAGGAACCCAGCCAC
ATTCTTTGGGGTCCACGTGTCCTCCACCCCACTAGAACTTTACTATTACCAGCTCAAGG
TTGCCTCTGGACACGTAAGTAAATTTCCATTATCCAGTTGTTTTCCCTTAAGCAGTGATT
TAAATATGTAAATGTTTTTTCTTTGAATGTCTATTTTATCCATGTTTTAAATATTAGTTGTT
AGTTAATTCATGGGTAGCATGGTCATTTTGATTAATTGCTTAATCTTGCATAAAGTTATTC
CGAATGGTTCCAAAATTGTCACGATTAGTTTGAGCCTGAGGAGTCAAACTTGGTTGGT
CGTGTTTTCTTGGGTGGTGGGGACACAGAGAGAGAGAGAGAGAGAGAGAGGGGAGA
ACTTAAATGTGGCCAAATTGTCCCATTGATTGCGGTTCGTGCAAAAGGGTGCAAGACT
TTCAGCTACCATACCTGTCTTCCTGCAGGGATAATTGTTGGAAATAGTTTTCAATAGAGA
CTCGATACCATAGCAATGAAGGTCACCAACATGTGGGGTTTAGATGTTTCCGTTGGATG
ACCGATAATGCAAAAGTAAAATAACAGAGACTATCGTAAGAGCATTTTCCTAATATCAA
TACTTAATGACCCATAAAAATTGATTGATAAGATGACTCGTATGCTAATAAAACGTGGGT
CCTTCATCTCGGGTAGAGCCCACACTCTATTAGTGTATGGATCATCTTGTGGGTCAATTT
TTCCCCTTTTCGGATCATTAACTATTTTCCTTTTCCTAATTATTCTACATTAACTTGTGGG
ATGATGGTTTTGGAGAGGTAAAACATTATCGATATGAGAAAATAAAGAAAATAAATATT
ACGCCCATTTGTGTTGACTAGAAAAATGATTTGGCGAACCGTCTCTTTGATTGCCTATAT
GTACTGCCTCTCTCACATTGGGTGGGATCCACGTAGGCCCCACATGAACATGGGTCCCA
TCTCATGTGAGAGAGACGGCACAGGAAGCCAGTAAAGTGGCGGTCCGTCTAGCATTGT
CCTCTTGGATAAAGTCATGCTAGTATGACAAACATAAAAAATCACAGTTATGATAGCCT
ATTTATTCACCATGAATTCAGAGAGAATATGATATCTCTGTTAAGTGGAGTCCACGCAG
GTCATTAAGTTCCTCAAATTTATGGTGGACAAGTGGCTGCCATGGTTGTCATTTCCTTTT
GTATGTTACACTAGGATTTTCTTAGTGAAGTTGTAAGCGTTCTTTCTAGCGTTTTGAACA
ATACAAATAAGAAGGAAAAGGAAGGAAAAACTAAGGAAGAAATAGGCTCTGTATACC
TCGTGTGTACTGCTTTCAGCTCATTTCTTATGCTGTAGTACAACTTTCTTTACCTTTATAA
GGAAAACAAGAAACCAATAAAATTATAAAAAGTAGTACAATATGAATTTATCCTTGCCA
CACCCTTGTCCATATCTAGGAGATTGGCGATATAACATTCATATTTAGGGAGAAATTATG
TTATCGGGTCAAAATGCCAACATGGTGGGAAACTTTATCTAATATGTGACATGAGTATTG
TGACAATGTCAATATCACATTGTCAAGAAATAAACTTAATTTATCGCTAGTTTACATATTA
CAACATTTCCACGATTCAAGAGTTGAAAGGGTATTTTGACCAGATTTTGAAATTTAAAT
TTGAAATCTCTGTCTTGCACGCATACACACACATTGGCCTTCCACAATTCTGATTGGTCT
TTGGCCCTTTGCTATCTTCACCATGCATGGTCTACGATGATTTTATTGACACAACTTTGT
GGACCATATATTGTCAATAGTTGAGCTTTCTACAAGTGCCATTAACCTTGTAAACGTTTG
AAATTAGGTAGAACACCTTTGTTATATTGAAACAAACATTATAATATTGTTAATCTTAGA
CTTAATGCATATTGAAGGAGAACTGCAATATTGAAATATGGTAACTTTGTTGATGATTTA
ATGCACAACTAAAACAACACCAATTAAATTGGACATGCTGTCTGGATTCAAATTATGAA
TTTTGGTAAAAAATTTCGCTAAGCTCTTGAATTGTGGAAACATAGTGAAGTTTAATTGA
GTTTATTTCTTGACAATGTGGCACTAACATCGTTACTATGCTTAGGTGACACATAGTAAA
TGGATCTTCCCACCACGTTGCATGTGCTAACTTGGTGAGAAGCTCCCAAAGGATAATTT
CTCTACAGTGAGGAAGCTGATATGACTTGAATTAACAAGGTTACAAACCTTGTTAATGG
CACTCATTTAAGCTGCATGCAGTATGCACTTGGTTAGGTTAGGACTTAGGACTATGATGT
TGTAGTTCCCCATTTCTTAAACTGCATATCATTTCAAAAACATGGTGTCAATAATTAAAA
TTTTAATATAGTACAACACTACAACTCATAAACATACTTGAACTTTATTCTAACTTTTCCA

GTTTAACCTCAGAGAATCAATTCTTTGGTCCATAGATATTGGCTTTACAAAAGGTGTGTT
GCTTGTCAAGATATTGTGTTTTTTATTTTTATTGATGTGGGTCTTTAGTGAAAATTTAACT
GTAGACCCACCCACCCTTTCAAATGTCTTTACCACTGTGCTATGAGCTCAGGTAGAAGT
CATCTTCCCATGGTGTTAGAGGTAAAGGCATCTGGAGGGTTGGGTGGATCTTAGTAAAA
TTCTCATTGTTGATTGAAGAAAATTGCATTTTTCTAAGAAAACTCTTTTGATAGCCGCTA
TGTTTTTTAGTTTTAAGTAAGCTCTATGTTTTCGGTATTTTTCAAAATGCTTGCATAATCA
AAGATTCGTTTGAATCCAACCTTTGTGATAATAGAGGTAAAATGATTTTCATTGACCTAT
TTAAAATTGGAAGACAAGGTGAAAAACAGCAAGTCATTTTACCTTTACTATATGTAATA
CGGGTTGAGTAAAATAAATCTAAATCATGTGAGGGGTGTTTCAAATTTACAAGAATTAA
TGGGTTTTTCTAAAAATACCTTAAAACGCAAGGGTTATGTGGAAAGGTCCCTTACTAAT
ATGAGTTCTTTCTTGTTAACCTTCTTTTTTTGGGATAGATTTTATACAATTGGCCAAAAA
CCAGGTGGTCTTTTGCCACAGGTGGTCCATGGTTAGGATCAATGTATGAAGACAAAAC
TATTGAAAGATTTGAATCTGTTAAGACACATGCCTTGATCCTGACAATGGACCACCATG
GTCCATTGAACACTTGAGTTTTTCCTTTACTCTTTGATGGAAAACAAAAATCCTAATATT
ACAGACCAATTGGTATCACAAGCATCACAACCCATTCATCACCATGAATTTTAACAAGG
CATGATGTCATATATGGGACCCACATCACAACAAATGACATCATGCCTTGTCAAAATTC
ATGGTGAGGGTTGGGTTGTGCTGTTTGAGATGTCTTTTTAGTATGATAATAGGATAACTC
AAAGACTAATACTTCATGTCTGCGTTCTATTTTTGGTATCTAGAACATTCATAACATTTTG
AGTTGATGAGTTTTAAACATTTTACTTTAGCATTATTTTCAATTAAATTTTATTTAGGTTTT
AATTCTTCACATTTGATTATAGTTATATGCTGAAATCATCAAATTGAGATGATATTTAGAC
CTGGCTCATAAGTGGACTTAACTGATAATGATTCCAAATAACTTCTCACTATAGCTGGAA
GCTTAAAGTATAGATAGTGAAAGTGATCATGTGATCCAGACCATAGAATTTTTTTCCCAT
AGAAAACAGTACACTACGTAAGTTCTCCATGACCACACTCCTCTTTGGTAATTATAGTC
ATCAAATCTTTTGTATCTATAAAAGATAAATAAAATAAAATAAAGACCCATCAAATCTTG
TTGGTGTTACCGACCCAAATCTTGCCTCATTTTTATGTATTGCTGATCAAACAAGTGAAA
CCCTTCAAAGGTTTGTCTTCTTATTTTTCTGATAGTGATTGCAAGATTCAAGGGACTACG
AAAATTATGGTTAACAATAAGAGAGTAAGCTTAGCCCTACTGTGATTTGACAGAGAAA
AGCAAGTATGGCAAGCAGGTGACCTAGGGCTACCAGGAAGTAAATTTATACAATCTGA
TTGGCCAAATGCACTTGGCAGGCTTTGTGAAATCTGTTCTTCTATGAGCTTCTTTTACA
AAAAGCAATCTCTCAATAATCACACCCCCTCGCAACATTTTTTTGTTAATATTTCAGCAA
TTCCTTGTGGGGTTGGCATTGTCTTTCTCTGATCATCTTTATAAAAATGTATTAAATAGAT
GAGGGAGTTCTATCAATCAAGGAAGAGCAATCAGAATGTTGTAACAATGGTGCTAGGA
TATCAAGTGCCACTCTATGGTGGGGTATCAGTCCTCGGAGGTGCTAGGAATCACTACGA
GAATGTCGCAGTGCCACTGAACTTGACATTTGTGATGAGATCCAGGGCCTACATTTTGG
GAAAGTTGGTGAAGTCCAAGTTTTACAAGAGAATCAGATGCTCTGTCACTTTAAGAGG
CAATCAGCTTGGCAAGCTCCAAAATTTGACGGATTCTTGTATTTACCAGTGA
>LsfLEA2-6
ATGGCTGAGAAGAACCAGAATAATGCTCTAGCACCCGAACGGCCCAGGAGTGACGAA
GAATCGGCAGCTTTGCAATCCAAGGAGTTGCGCCGCAAGAAAAGGATCAAAATCGCTT
TATATATTGTTGCCTTTGCTGTGTTTCAATCTATAATCATCGCCATCTTTGCTGTTACTGTG
ATGAAGTATAGAACTCCTAAATTCAGGCTGGGCAAGATCACTGTGGAAACTCTCACCA
CCACCAACACGTCTTCGCCTTCGTTTAGCACGAGGTTCGTTGCCCAAGCTACCATAAA
GAATACCAATTTTGGACCCTACAAATTCGACAACACCACGGCTGTATTCACATATGGAG
GCGTGAAAATAGGCCAGGTCACTATTCCTAAAAGTAAGGCTGGGTTTCGTTCGACCAA

AAAAATTGACGTAACGGTGGACCTGAATTCTAGTGGGTTGACGAGCACAGAAGGTCTT
GCCAGCGATATCAATAATGGGATATTAAAGGTGAACAGCCATGCCAAGATTAGTGGGAA
AGTGGAATTGATGTTTGTGATGAAGAAGAGGAAATCTGCTGAAATGAATTGCACCTTG
ACATTTGACTTGTCTGCACAGACACTACAGGCCTTGGAGTGCAATTGA
>LsfLEA2-38
ATGGCTGAGAAGAACCAGAAGTATCCTCTATCACCGGCAGCAGCCGGACACCCTAGGA
GTGACGAAGAATCAGCAACTTTGCAATCCAAGGAGTTGCGCCGCAAGAAAAGAATCA
AAATCGCTGCATATATTGTTGCCTTTGCTGTGTTCCAATCTATAATCATCGCCATCTTTGC
TGTTACTGTGATGAAGTATAGAACTCCTAAATTCAGGCTGGGCAAGATCACTATGGAAA
CTCTCACCTCCACCCCCACGCCTTCGCCTTCGTTTAGCTCGAGGTTCGTTGCCCAAGCT
ACCATAAAGAATACCAATTTTGGACCCTACAAATTCGACAATACCACGGCCGTTTTCAC
ATATGGGGGCATGACAATAGGCCAGGTCGTCATTCCTAAAGGTAAGGCTGGGTTTCGTT
CGACCAAAAAAATTGATGTAACGGTGGACCTGAATTCTAGTGGGTTGACGAGCAAAG
ATGGTCTTGGCAATGATATCAGTTCTGGGATATTGAAGGTGAACAGCCAGGCAAAGATT
AGTGGGAAAGTGGAATTGATGTTTGTGATGAAGAAGAGGAAATCTGCTGAAATGAATT
GCACCTTGACATTTGACTTGTCTGCACAGACACTACAGGCCTTGGAGTGCAATTGA
>LsfLEA2-45
ATGGCTGAGGAGAACCAGAAGTATCCTCTATCACCGGCAGCAGCCGGACACCCTAGGA
GTGACGAAGAATCAGCAACTTTGCAATCCAAGGAGTTGCGCCGCAAGAAAAGAATCA
AAATCGCTGCATATATTGTTGCCTTTGCTGTGTTCCAATCTATAATCATCGCCATCTTTGC
TGTTACTGTGATGAAGTATAGAACTCCTAAATTCAGGCTGGGCAAGGTCACTATGGAAA
CTCTCACCTCCACCCCCACGCCTTCGCCTTCGTTTAGCTCGAGGTTTGTTGCCCAAGCT
ACCATAAAGAATACCAATTTTGGACCCTACAAATTCGACAACACCACGGCTGTTTTCAC
ATATGGGGGCATGACAATAGGCCAGGTCGTCATTCCTAAAGGTAAGGCTGGGTTTCGTT
CGACCAAAAAAATTGATGTAACGGTGGACCTGAATTCTAGTGGGTTGACGAGCATAGA
TGGTCTTGGCAGCGATATCAGTTCTGGGATATTGAAGGTGAACAGCCAGGCCAAGATT
AGTGGGAAAGTGGAATTGATGTTTGTGATGAAGAAGAGGAAATCTGCTGAAATGAATT
GCACCTTGACATTTGACTTGTCTGCACAGACACTCCAGGCCTTGGAGTGCAATTGAAG
ACAATAGTTTAGCTGTTTTGAGTTGTTGTTTTGTATCTTTGTATTCTTTTATTTTGTTATTA
TTGTTATTATTTTTTGCTGGGATTGTATTCTTTTATTTACTGTTTTTGTTTTTTTCAATGAA
AATATACTGAGGCATTATTATTAACTATGGTGCTTAATTTCTGGATTAGATTAATGCTTCA
AGAATCAATTTCATCAGAAATTGTGTCTAAACTTTATAGATCAATCCTAATTTCTAAACA
AGGATTGGGTGACTAATTACTATGGCTGGTAAAAGCTTTCAGGGCAGTTTTTAACAGTT
GATGTGTGTATATATATATATATATATATATTATAAAAGATTCCAGCAGACCTGTTCGGCCA
CGTATTCTGATAATTTTTAGCAAAAAGGAAATAAGAAATATGTTTGAAAAATGTTGTCC
GAAACTGTTTTCTGAAACAGTCCATTTTCAACTTAATTTCTGCTTTCTGAGTAGGAATA
AAACCATTGTAACAGTCATGATCTCAAAATCTCTCACCCGAACAGAAATATATGGGTAT
TCTGTTTTTGCAGCTTCCGCCTAGCAGGAAGAGGCCAAAGCCATGGTAACTGCTCTTTT
TTGCGCAAAACCCAAATGTCTAAATTTTCGTTGAACTTGGTTTTTGTGGCAATTTCCCT
ATGCAAGCAGCCAAGGCATGCTACTAACGAATTGATGCAGGTTAATTTCATCAATCAAC
TTTGAACTAGTTCTTGATCGATGCTAATGATTCAATAATGGAACTTTGGCATGTGTGGCG
ATACGGCTAAGTTCATGCAAGCTGTATTGGCTTTTACAAGCAGATTCACATCGGAAACC
ATGTATGTGCTAGAACTGATGGGGTCAGGGGCCAACCATCGGTTAGGGATCTGGAACA
GGGTCTGGTTAGGAAAATTAAAACATGTAACATATGATAAGAGTACAGAAATGTTAGAA

GAGAAGTAGAGGGATAGGCACATTGCAATTAG
>LsfLEA2-5
ATGGTAGAGAAGGACCAGCAGGCGTATCCTCTATCACCGGTGGCAGCCGGACACCCTA
GGAGTGACGAAGAATCAGCAACTTTCCAATCCAAGGAGTTGCGCCGCAAGAAAAGGA
TCAAAATCGCTGCATATGTTGTTGCCTTTGCTGTGTTCCAATCTATAATCATCGCCATCTT
TGCTGTTACAGTGATGAAGTATAGAACTCCTAAATTCAGGCTGGGCAAGATCACTATGG
AAACTCTCACCTCCAACCCCACGCCTTCGCCTTCGTTTAGTTCGAGGTTCGTTGCCCAA
GCTACCATAAAGAATACCAATTTTGGACCCTACAAATTCGACAACACCACGGCCGTCTT
CACATATGGGGGCATGACAATAGGCCGGGTCGTCATTCCTAAAGGTAAGGTTGGGTTTC
GTTCGACGAAAAAAATTGATGTAACAGTGGACCTGAATTCGAGTGGGTTGACGAGCAC
AGATGGTCTTGGCAGCGATATCAGTTCTGGGATATTGAAGGTGAACAGCCAGGCTAAG
ATTAGTGGGAAAGTGGAATTGATGTTTGTAATGAAAAAGAGGAAATCTGCTGAAATGA
ATTGCACCTTGACATTTGATTTGTCTGCACAGACACTCCAGGCCCTGGAGTGCAATTGA
>LsfLEA2-2
ATGGCCGATCAAACTAGACACAACAAAATCATCAAATGTTCAATCATTGTAGCTACAAT
AGTAGTCCTCATCGCTGTTTACTTGATCACAGTGAAAACCATTCAGCCGCGTCCACCAA
GATTTCGAGTAACCTCGGTCCTCGTTTCTCCCTTAAACCAGTTAAACGTCACGACCTTA
AACAACAACTCTACCTCTCAGCAGATTACTACCGCCTGGCGCATCGACTTCCTTATTAC
AAACCCTCACAAGTGGGAGAGCATATCTTACGAACAAGTAGAGGCTTCAGTCTACTAC
AAGAACAACTTTCTATCATCAACAATCATACCGCCCTTTAATCAGGATAGAAAGGAAGA
GTTGGGGCGGCGAGTGACTGTGGCTGCCTCGTCTCTAGAGGTCAACAATGGAGTCATG
AATGCCATCGCGGCCGATATGTCGCATGGAGCTGTGGGTTTTAATGTGATGGTGGTTGC
TAGAGTTAGGGTTGATAGGAGGTACACATGGATACAAAAAATGCGTGGGATGAGGGTT
ACTTGTGAGGATGTCAAGGTGGGACTGTGGTCTCATACAACGGCTGGGACTATGCTTG
GTGGGTCAACGCCATGCAACGTTCATCTCTAA
>LsfLEA2-44
AACTTCCACACCTCTCAAAACTCTCTCCACCCCACTCCTTCCTCCACTACCACCACCCC
TTTCACCACCGTCAACCTCCCCAGTAGTACCCACCATGTCCTCCAAAGACTGCGGCCA
CCACCACCACAAATGGCGCAAACGCCTGAGGCGTCTCTGCATCGGCCTCCTCATCTTC
AACTTCATCCTCCTAGTCGTCATCCTCCTCGTCTGGGCCATCCTCCAACCCACCAAACC
CCGCTTCATCCTCCAAGACGCCACCGTCTTCGCCTTCAACGTCTCCGCCCCCAACACC
CTCACCTCCAACATCCAAGTCACCATCTCCTCCCGCAACCCCAACGACCACATCGGAA
TCTACTACGACCGACTCGACGTCTACGCCGTGTATCGGGACCAGCAGATTACGCTGCGC
ACCAGCATCCCTCCCATCTATCAGGGCCACAAGGACGTCAACGTCTGGTCGCCGTACG
TGCTGGGCAACTCCGCTCCGATCGCTCCGTACAACGGGCTTGCTCTGAGCCAACGATC
AGGCGAGTGGGAACGTGCTGGTCTTGATCAAGATGGATGGACGGGTGAGATGGAAAG
TTGGGACATTCATATCGGGGAAGTATCACCTGTACGTCAAGTGTCCGGCGTATCTCACC
TTTGGGAATCCTAATACCGGTATGGTGGTTGGTAACGCCGTTAAGTACCAGCTTGTGCA
GCGGTGCAGCGTCAGTGTTTGAGCCAGTTAACGGCCGTTAAGTATCACTCATCTCTCTT
TCACGCCGTTATTATCTTCTTTTTTTTTTTTTTTTAATAGTTTAATTAATCTGTGTTTTAAT
CTCTTTCTCTCTCTATAGCTTTCTCCTTCTAGAGGTATACGTGGGATGGACAAAAGTCTT
TGAATTTGTACATTTTGCTATAGAATAAATTGGAGAAGAAAAAAAAGAAAGTGTGAGA
GAAAATAGCAAAATTACAACGTTGGGACAGAGTTGTATAATTGTCATTAGTACAAATTG
TCCAATAATTATTTTGAATTTCCAATGGATAATAATTGAGTGTCGAGTGGAGGTTGTAGT

GTCCTTTTCTTTCTTTTCTATTGAATCGAAATTAATATCCTATTGTCGCTTATT
>LsfLEA2-46
ATGGGCAGAGGAAAATGCTTAGCATATGTGGCAGCCTTTGTGGTATTTCAGACCATAAT
CATCCTAGTCTTTGCACTAACCGTGATGCGTATCAAGAATCCGAAAGTAAGGCTAGGCG
TGGTCGCCGTCGAGGATTTCAGCACCACCAACTCCACCTCTATCAACATGAGATTGGTG
GCCCAAGTGACAGTGAAGAACACAAACTTTGGTCACTTCAAGTACGAAAACAGCACA
GCCACTATCTCATACGGTGGCATAGCAGTTGGTGAGGCCTTCATTCCAAAGGGGCGCG
CGAGGGCTCGGCAGACTAAGAAATTCAATGTCACCATCGACGTCACTACTGACCGGCT
GTCGTCGAGCAGTTCGAACCTAGCGAACGATATTAATTCAGGGGTTTTGGCGATGAGT
GGCGAGGCCAAGTTGAGTGGGAAGGTGCATTTGATGAAGGTGATCAAGAAGAAGAAA
TCTGGTGAAATGAATTGCACCATGGCAGTCAATTTGGCCACGCGTGCTGTTCAGGATTT
GAAGTGCGACTGA
>LsfLEA2-36
GCATCACCTGAGTTTTTTTCCTTTTTTATCTTTTTTGGTGGGTAAATTCGTATTATTCTGA
TTTACTAAGTTGTAAATGGAAGTAGCAGCAGCATCATCATCATCATCAAAGGATGGAAA
AGCAGTCAAACGCGTTCGCGCCCGGAGACGCAGAACCATCTGTATAGCGGTGATCGCC
GCTATTCTCGGTGTCGTTCTGCTGATCGTCATATTAGGGCTTACCGTCTTCAAGCCCAAA
CGTGCCGTTACCACCGTCAACTCCTTCAAGCTGGATCATATGGACGTCTCTATGGACGT
TGCTAGATTGAGAGTGAACTTGAACATCACTCTCAACATCGATCTCTCCGTGAAAAAC
CCTAACAAAGTTGGGTTCAAGTACACCAACAGCTCCGCCCATTTGAATTACAGAGGCC
AGGTTGTGGGAGAAGTGCCCATCCCCGCAGGGAAGATATCCGCCGACTCCACAATGCC
CATGTCTGTCACCCTCACCCTATTGGCCGATCGATTCCTTTCCAATTCCGATATTTATTCC
GATGTCCTCTCCGGTACGTTGCCCCTTAGCACCTATACCAAAATTTCTGGCAAAGTTTA
CATTCTCAAAGTCTTCAAATTTCATGTCGTTACTGAGAGCACATGTGATGTGGTTGTAA
ATGTCTTAAACAGAACTACTCCCACCCCGGAGTGCAAGTACAAGACAAAATTATAATTA
TATTACATTTCTGTCAGCCTTGGATTTTGGACGACTCTACAATAAAACTTGTTTCATTCA
AGA
>LsfLEA2-24
ATGGCTTGGACGGCGACAACATCAACAACCGAAGCCCAAGACAAGACAGTCACCGGA
TACCCAGCTCAGCCGAGCCAAATCGCCGCCGGCTACCCACCACCAACCACCTTCCCCG
TCACCACCGCCTACCCTTACGCCGTCCCACCACCCGCCGTCTTCTCCCAGACCTACCAC
CCCCCTTCCCCTCCCCGATGGCGCTACTACCGCGAGGGCGGTCGCTCCACCCTCCTCTG
CCGTCTGGTCTCCGCCGTATTCGCCGTCCTCTTCGCCATCGCCATCTTCTTCTTCATCAT
CTGGCTCATCCTCAAACCCCGCCTCCCCGAGTTCCAAGTCGACTCGGCCTCGGTCGCC
CCACTCAACGTCACCAAAACCGAGTTATCTGCCACCTGGGACATCACCTTCCGAGTCA
GAAACCCTAACGAGAAACTCAGCGTCTACTACCACCGAATCGAGGCTTGGGTCGTGTA
CGGAGAGGAGGACCTTCTCTACGGTACGGATCTGCCTCCGTTCTATCAGGAGAAGAGG
AATGAAACGACGGTTGAGGCTCAGCTCGCCGTGGTGTCGGACACGGCTGACGGATAC
GTTCTGAGGGCAATTTCGGAGGAAAAGGCTCGTGGGTTGGTGAGTTTCGATGTGAGGG
TTTTGGCATTGATCAATTTCAGAAAGGGGGTGTGGCAAACGAGGCGGCATTTGTTGAG
GGCTTCTTGCGAAGGGGTGATTTTTGGATTTTCGGCGCCGAATAATGGGACTGGGACCT
TGATGGGTGGTCCTAGGGCATGCAAAGTTGGTCTGTAA
>LsfLEA2-48
ATGACACTAGAGAACCACCACCAAGTGTCCTCCGAGAGGAAACAGAGGCAAAGCACA

CCGAAACGCCGATGTCTTATTGCAGTTGGGGCGGTTCTCCTAATGTTGCTCTTGCTGCT
ATTTGTCATTGCCTTAATCCTAGCCTTGACGGTGTTCAAGGCAAGAGAACCCAGAGCC
CAAGTGGTTTCGGCTGCAGTGGAGGGCATCGCACCTCGAGTCTCATTGCCGCTTATCC
GAATACAACTCAACATCACCTTGGACCTCAAATTGCTGGTTGAAAACCCCAACCACGC
CAGCTTCAAGCATGGCACGGGCAAGAGTGTCCTCCTGTACAACGGCCACCAGGTGGG
TGAGGCTGACATATTCCCCGGAAATATTCCGGCCAGGGGCTCCACCACGCTTGCGTGC
CGTCTAACACTGCAAGTAGATGAGTTGGCATCGGACGTGACCCCTCTCATCCGTGATGT
ATTGGCCGGGGAACTGCTCATGGAAACACACACTGGGATTCCAGGCAGGGTGTCCTTC
TTGGGGATTCTCCGCAAACACGCTGTCGCCATCTCTGATTGCCAAATAGCCATTGGAAT
TCCAGACATGAAGATCCGAAGGCAAGATTGCAAGCAAAGGGCCAAGTTGTGA
>LsfLEA4-1
ATGGCCGAGCTAAGCGATATCAAGAGAGAGGATGAAGAAGCAATAAAATGGAGTCTG
AAAGCAAAGAACAACAAGCAGGAGAGGTAACGGTGGTGTTCTTGGAAGCATCCTCAA
GGCAGCTGACGAAAGTACCACGACCAAGCCAAAGAATCTACCGTCGGTAAAACTCAT
GATGCTTGCGAGAAAGCGAGAGAAGATGTTGACGTGGCTTCCGACAAGGCCAGGGAG
GCTAAAGAAGGGGCAGAGGAAAGATCTCAGGAGGAGAAGGGGAAGGTGGGAGAGTA
CAAGGATTATACAGCGGAGAAGGCGAAAGAGACGAAGGATTCGGCATTGGAGAAGGC
CAGGGAATACAAGGATAGTGCGGCGGAGAAGACCAGAGAGACGAAGGATTATACCAT
GGGGAAGGTGGGAGAGTACAAAGATAGTGCGTCGGAGAAGGCGAAAGAGATGAAGG
ATTCGGCATTGGAGAAGGCGAGGGAGTACAAGGATAGTGCGGCAGAGAAGGCCAGAG
AGACGAAAGATTCTACCATGGGGAAGGTGGGAGAGTACAAGGATAGTGCGGCGGAGA
AGGCCAGAGAGACGAAGGATTCTACTGTGGGGAAGGTGGGAGAGTACAAGGATAGTG
CGGCGGAGAAGGCTAGAGAGATGAAGGACTCAGCGTTGGGGAAGGCAGAGAGTACA
AGGATAGTGCAGAAAGAAAGCGAAGCAAGGAAAGATAGTAATCATGGAGAAGATGGG
AGAGTATAA
>LsfLEA4-2
ATGGCTCCTGGGATGAAGGGTAAACACTAAACAGATCCCTTCCAACCTAGGCAACAAT
TTCAAGAGTTAAAATTGAGAAAATGTAGCAAACATTTCCACATATAAAAATGTCAGGTT
TGTTTGTTTTACATCCGCATTCCTAGCTAGTTGGCACTATTTTGGTTGCAACCATCAAAC
TCTATTAAAGAGTGAACTAGAGGAAACATTTCCAAAACAGAAAACCCACGGATCTCCG
GGGTTGATAGCATTCATTCATACTTAAATTTGCAGACAGATTCAATAAAATCTTCATTTC
GTCATCCTAGAAAAATTGAAGCCACATCTAAATTCGATTCATGAAGTAAAGCCAGAAA
ATATATGAAGTAGTTCCAAGTTACAAGTTCTCGCACAAATTCAAAACCCCAACGGCAA
GACAGAAAAAATAAAAATAAAAAATGATAGGATTAACTCACCACTGGATCGCTGCTTG
GCGAAAGCTTAGAGAAGAAGCAAAGACCGCTTGGATTGGTTGCTCACCACCGCCGCT
TCGATTTCGCTTCGGAAGGAAGTTGCACAATTCAAAACCCCAATCAAAAAAAAAAAA
ATAAATAAATAAATTGATACGATTAACTCACCACTGGGATGCATCCATCTCGGCTTCAAA
ACCCCAACCCAAGCAAAAAAAAAAAAAAAAAAAAAAAAAATTGAAAACTCTTACTCA
CCATCGGGGCTTTGGGAATGACCACGGGATTGTTAGAGAAGAGAAGGAGATTGCATGG
GGAAGCTTCACCACGCTGGTCGGGATGGGGGCATTCCACTATTTTCGCTTGAGAGAG
GAGCTGAGAGGAGGAGCTCCTCACCACAAAATCGGGATGGGGGCCTTCCACTATTTT
CGCTTGAGAGGAGAAGTGGAGAGAGGGAAGAGAGAGAGAGAGAGAGAGAGAGAGA
AGCCAAAATCAAAAACCAGATCTGAAAAGTCCTCCCTTTTTCGCTTTGGTGTGATTTTT
GCAAGTGAAAAACGGTGCCGTTTTTGGAACCCAACTAATGGCGTTAAAACCGCCTAAA

CCCAAGCATTTCCATCCCAACCATTGGATATGTGTCTTGTCCACATGGAGCAATCTGAA
GCTGACTCATGGAGAGTCCAAAGAAAAGGCCCTTCTTCGAGCAAATCCAAATCCAACC
AGATCTCAAGATTTTATAAAAATAACACTTTTAGCATGAATGATAACCAAATCATAACTA
AAAAAAAAAAAATATATATATATATATATATATATATATATATATATATATATATATATATAT
ATATGAAGGTGTGAAGAAAATCCATTTTTATTATTTAAGTGGTCATGATCTAGTTATAAA
AGCAATAATAAACCTATCATATTAGATTTTTTCTTATGAAAAATATGAAAAATCTATTGTT
TAAATTTAGCTTTTGGCCATTTAAGTCTCTTTAAATGGCATATTTTGAAAAAACTAGAAA
GTTGTTTGAAGTCATATTTTCTAGTGGACAATGAGTCCAATTTTCATGAAAATATTTAAA
ATTTTTATAAAACTTTGACCCTATATTTGGTTCATGGATTCTACCAAGGAAAAAAAATGG
AAGGGAAAAAAAATGAGGAGGAAAGAAAATGAAAGGAAAAGATTAAATCATATCAAG
CCAAAATTATTCCAATTATTCTTTTTCCTCTAATTTTCTATTGCAACCAAATAAAGAAAA
GTACAATTCTTCATATTTTATTTTTTATCCTTGATAAAATCCTCAAACCAAATAAAGCCTA
AGAATTTTCAAATAAGTGAGGAATGTCAAGAGAATTGAAAGGTAACAATAGCGGGCCT
TATTTTTAAAGATGCTATCATGCCCAATGGGCCAATGGGGGCGTAATAGGGAATGGCTAT
TGCAATCCCCCATTCTTATCACTCCACCCCTCATCCTTTTAAGTTTTTAAGGTTTTATGCA
AAATATAAAAGGTTTTTATGAAAATTGGTCGTATTGTCTATTAGAAAACATCATTTTGAA
CAACTTTGTAGAGAAAGTTTTTCCTAAATCTATCGTTTAAATATACTTTTTAGCCATTTAT
AGCCTTTATTTCATTTTTTGTCAAAAAGTCTACCTAAACGATAGATTTTGGAAAAACTTT
CTTCTGCAAAATTATTCAAAATGATGTTTTCTAGTGGACTATACGACTAGTTTTTGCAAA
AACCTTCTATATTTTTCATAAAATTTTAAAAACCAAAAAAAAAGAAGTGGAGTTATAAG
AATGGGGGGTTGCAAGAGTCATTCTCGACGTAATATTACGTTCATAAAAGGGTTGGGTT
GGGTTTAGAAAAATAATGGTTGGGTTGGGTTTAGAAAAATAATGACCAACTCTAAGCA
ATTTCATCCTAGGAAATTGGCCTCCCCCGAGTGTTTATATCAAAAATTGGATTGAAAATG
TGACGATTTTTGGGAGTGATAATAGCAGAGAAATTTGATTGGTCGAAAAATATAATGTT
GCTTATTCTGTGCATAGAATTTTTATCCTCACACATCGTGGGAATTATATATACTCTGAAA
ACATGTAAATGCCACACACACGGTGAACGGCAGGGTTGAAAAAGAAATTAAATTAAA
ATTTCTCGAATGATGTGATGAGCCAGACAAGCTGGCCACCAACCAGGCAAAGAAGTTT
GACCCGATATGCCACGTGGTCGGACACGCGGGAGGTGACCGTCATCGATCAAAGCGAC
AAGAGCCATGAAAATCCGGCTACAGACAAAATCATAGAGTGGCATGCAAGGCCTTGTG
TCCTTTCTCGACTTTGCATGCAAGACAACGTGTCACTCTCATCGTGACCCTCCTCTACG
ACGTGTCGATCATTCACTACACACACGCTACTGCCAATGCACTCCTCCACGCGTCATCT
CCACATGCAACGCCGAGAAATTATGTATATATCTATATTTTTGTGACATTATATATAGAGA
ACACAAAGACCGTGTTTTTAATATATTCTATTACATAGCGTTTTGTGTGAAATTTTGCAA
GAAGGCATGGCGTCGAGGCAAGCTAGGAGAACGAAGTGAGGCGATTCTTGAGCAAGC
AAGCGGCGTCGAGCTAAGCGATATCAAGAGAGAAGGAGTATGAAGAAGCAATGAACA
TAGAGTCTCGAAAGCAACAAAGAAGCAGAGGAGTAGTAATCTTGGTGGTGTTCTTGG
AAGCATCCTAAAAAGCGATGTGGCGTACCACGACCAAGCCAAAGAATCTATCGTCGGT
AAAACTCATGATGCCTTGAGAAGGCGAGAGAAGGTGTTGACGTGGCTTCCGACAAGG
CCGAGAGAGGCTAAAGAAGGGGCAGAGGAAAGATCTTAGGAGGGAAGGGAAGGTGG
GAGAGTACAAGGATTATGCAGCGGAGAAGGCGAAAGAGACGAAGGATTCGGCATTGG
AGAAGGCGAGGGAGTACAAGGATAGTGCGGCGGAGAAGACCAGAGAGACGAAGGAT
TCTACTATGGAGAAGGTGGGAGTGTACAAGGATAGTGCAGTGGAGAAGGCGAAAGAG
ACGAAGGATTCGGCATTGGAGAAGGCGAGGGAGTACAAGGATAGTACGGCGGAGAAG
GCCAGAGAGACGAAGGATTCTACTATGGGGAAGGTGGGAGAGTACAAGGATAGTGCG

GAGAAGGCCGAGAGACGAAGGACTCAAAGGCTGGGAAGGCAGGAGAGTACAAGGAT
AGTGCCGCAGAGAAAGCGGGGGAAAGATAGTATCATGCGGAGAAGATGGGAGAGTAT
AAGGATTATAGTCCGAGAAAGCGAAAGAGGGAAGGAAGCTACTCTTGGCAAGTTTAG
TGAGCTCAAGGAAATCTGCGGTGATGTCGCTCGAAGAGCCATGGAGTTCTTGTCTCGC
AAGAAAGAAGAGCAAAGGAGAAGACGCCGAGATCGGGGAGGCGACCAAGGTCAATC
ACATTAACATGGTAGCTCATAAATTCCTTCAGATGGGAGTTATTCAAATACTCAAGCTAG
TAATGATAGCTAGTAGTGTTACTCTTTTAAACTCGGAAAATTGTAGTATAAGAAATTAAT
GATGAAGTGGGATTTTGTAGGAGAAGCTGAGCGAGACGGAAGAGGAAGCCAGACGG
AAGATGGAAGAGATGAAGAGATGAAGCTGGAAGGACAGGAGTACAAGGATGAAGCT
GGTCAGAAGGCCCAGGAACCAGAGATGACACTGAAGCGGAAAGGTAAGGTCAATTTA
ATTATGCATGCTTTATTATTGTTACCATTATTTAATTATGCATCTTTCTCAACATCGCAAAT
AATGACATGATAGTCTTTTACTTTGAATTTTGAAGTTGAAGTGTGAGTCAAAAATAGAT
CCAACACCAATAGGTCACTCTGATCAGTTGGGATCTTGAATTCGGGAAAAAATTTAAG
ACATAGCGGGCCGTATAGGATTGCTCTTGCTTAAGTATGCATGTGTATATGTCTATATATA
GATGGCTTTGATTTTGAAATTGATTGATTTGTTGTTGCTTGTGGGATTGGGTAGGGTTCT
GCAACTAAGAAGCAACATATTCACTCACTTTCAGCGCGTGTGAAGCCATTAAGGAAAG
CTAACACATCCAACCGACGTCGTTGAGGAGACGCGCGCGCGACGTGAGCATGGTGGT
ACAGGAGGAAAGGTGGAGAGAAGGTGATGTGGATGTTCAAGAGACTCCTGAACTGGG
CGTGGCGCCGACTCTGAAGGCGTCGGATCAGATGACCGATCAAACTTTCAACGACGTT
GGACGGTTGGGCGATGAGGGGGTTGCACGTGATGACCGCCAGAGCAAGATGTGA
>LsfLEA1-4
ACTACTGAGCTTTGTTGCGAGTGTTTCCAAAGCAAAACCAAAACAAAGAAAACAAAA
ATGCAGGCTGGAAAGAATGCAGCGGCATCTGCCAAAGAAACTGCTGCCAACGCAGCG
GCCTCCGCTAAATCTGGCATGGACAAGACCAAGGCCACCATGCAAGAGAAGGTCGGG
TCTACTTGCCTTCATGTGATGATACAGTGTTTGCTTTAATTTTGTTATTGATCTCATGTTC
ATGTGACATTTTATTTATTGTTTTTTTAGAGGGGTTTTTTCATATACTTTTTCCGTTCTTAA
ATAAGTGTCCTATTTTTTATGTTTCAAAATAATTGTTCTATTTCAAAAGTCAATGGTCATT
TTTTATTCCTTTTTTTAAAATACCCTTAAAAAAGTATTCTTATTTTTTCTAATGTTTTACAC
ATTTGAGGGCAAAATTGGAATTTCAATACATATGTATTAATATGAAAGCATTTAATAGTG
TTTTCTCAAAAAACGTGCCAGATTGATGCATGTGCAAAAGATTCTCTGTTCAACGCATC
TAAACTTCTCTATTCATCTTTAAAACTTTAAAAAATCTTGTGTGTCGTCCTTCCTTTTATA
AATGAGAAAAAATTTTCTACCAAAATATAAAAAGGGAAATTTGTTTGAAGGAATAGAA
GTAAATCATCTAAAAGCTTGGATCTGAGGATGTGTGTAGTTGAGATGCTTTTAGGGTTT
GTAAGATAGAGGGGTATTATGAGAGAGGATGAGATAAAGGAATTTTATGTTGGGTGGG
AAATGGGTGTAGATAGATATGTGTTTTTATATATTTGTATTACTGATATATTCTAAGACAGT
CGATTACACAAAGTAAAATTGTAATACGTACACAATAACAATGATAGCTCACTAAATTA
AATCATCCATAGAGTTTTTCATAAATAAATACTCATCCACATAAAAATTTGGTTGATACC
AAATAATTAATATTAATATTACTACTATTATTTTGGGTAAAAACACTTTCGTAAACTATAA
ATTTTGGGGTTTTTCAATTCAATACCCAAAGATATATATATATATATATATATATATATATA
TATATATATATATATATATATATGTATATATATATATATATATATATATATATTTATTATAAAAAA
ATTACTATCTAAATAATAATTAAGTGTAGTCCAACCGTAGATGGTCATATAGGAGAAACG
GAAAATGGACATGACATCATGTGCATGCATTGCTAGAGCTTACACGATGCCGTGTAGGC
TTTGGCAGGACACGCACATGAAGTCACGTTCATTTCCCGTTTAAATCCTAGTGTAGGCA
CACACATGTAGTTTATGGTGGATTGTATTAATTAATTGTACTTGCAACACCTTTGTATTTT

TTGGTATCTTTGTACTCGTATTTGTTATATCTTGTCCTCATTGTTTTATAGTGTTAAGCATA
CCTATAGAGGAAGAAACTTTTTTACTTCTAAGGGCGCAAATGGAATTGAATTTTGTTGC
TCTTTGGACTTATTGAAGCAACTTATTGACTTATGCTCCATTGTATTTTATGATAATCTTT
AGTTTGATATTTTTGCAAATGTAATGCTTATCTTTTGGTTTAATAAACTTGGTAAGGGTCT
ACCGTAACCCCACTTTCTTAACATGGACTGTTTTATTAATATATATATATATATATATATATA
TATATATATATAAAAACCCAACTATAATTCATGTATTAAATTAGAAAAAAATTTCTTTAAA
TATTAGATTGAAAAATGTCACAAAATGTATGGTACTAAAGTGATTTTTACCCTTTTACTT
TTATTTTGAATTAGCTTGATATCTAAAATTATTCAATGAAAGCTCCTTACATAGAATGTCA
AAAAAAAATACATACAATACGTGTTTGTTACTTTTATTTTGAATTAGCTTGGTATCTAAA
ATTATTCAATGAAGCCTCCTTACATAGAATGTAAAAAAAAAAATACATACAATACGTGTTT
GTTTGAGAGAAAAATGATATATATACTGGTAACACATACCGATTTCACACACCAATTAAT
GTGCAACAGATGTTATATTAATTATTGAAAGAGGCAACTAGGTAAGCCCTTTCTATTAAA
AGAAAACACATGCTACGGTGAACCAATGGATGAATCCGATTGATTCATCCGATATGTAT
AAACATCTCTCAAGAACAATAATAGTTTTTATAGTTGATAAAAGCAATGTGCATCTGGA
AGGAAAAAAAAAAAAAGCGATGTGCATCTCAATAAAAAAGATATTTATTTTTTCAAAAA
AAAAGGTTAATGTGCATGCATGCAAAGAAAACTGCAATCGTCAACAGGCACGACACTC
GGACCAAAAAACGTGACACGACACGTATTGCAACTTCGATTATCGTGTCACCCTAGAG
TTTATATAAGGCCGCCACGTGATCAAATGTCTCATGACTCCGTGGCATCCCTCTTAAATC
GGACCACATGACACGTCCCTAAAACGTGTTTGAACCAAAATCTACGTACCTCTAAATAA
CTTCCACGTAACTCTCTTCAAATTACACGTGTCGCCAATCCACTGCCCCTCGTACGTGA
ATTTAAACAGGGATGTGGGAACTTCACAGACCCACTACTGAGCTTTGTTGCGAGTGTT
TCCAAAACCAAAACAAAGAAAACAAAAATGCAGGCTGGAAAGAATGCAGCGGCATCT
GCCAAAGAAACTGCTGCCAACGCAGCGGCCTCCGCTAAATCTGGCATGGACAAGACC
AAGGCCACCGTGCAAGAGAAGGTCGGGTCTACTTGCCTTCGTGTGATGATACAGTGTT
TGCTTTAATTTGTTATTGATCTCATGTTCATGTGACATTTTATTTATTGTTCTTTTAGAGGG
TTTTTTCGTATACTCTTTTCGTTTCTAAATAAGTGTCCTACTTTTGATATTTTAAAATAATT
GTCCTATTTCAAAAATCAATGATAATTTTTGATTTATTTCTTAAAATACCCTTAAAAAAGT
ATTCTTATTTTTCTAATGTTTTACATATTTGAAGGCAAAATTGGAATTTCAATACATATGT
ATTAATATGAAAGCATTTAATAGGGTTTTCTAAAAAAAAACGTGTTTTTTCAAAGTGGA
CACTTAATTTGGAATGGAAGGAGTATAAAAAAAAATTATAATTGATTGATCTAGTGGCTA
TAACATCAATTTGAGCAACTGGTTAAGGTGTTTAGTATTCGGATTATAATATTTGGGTTC
AAATCTCTACAGTCATTTGGTGGGACTTGGAATTCGTGGTGAGACTTAAATTTTGGTCT
GAGTTATCAAAATATAATATTTCTTGACGGATATGTGAAATTACGTATTTATCTACATACC
CTTTTGTAGACCAAAAATAAAAATAAAAAAATAACATCAATTGAACCAGTAGGTTTAGA
TTAAAAAAAAGTTACAACGTAAATATTAATTTTCCCTAGCTAGCCAAAATAAAATTTAA
GAGACTTGTCATGAGACTCTTTTATCAGTTTTATAGTACATATTAATTTGGGTGAAAAAC
AACCATCTGATCTGTTGTGGTTGCCCAGGCGGAGAGGATGACAGCACGTGATCCAATG
CAGAAGGAAATGGCCACAGAAAAGAAAGAAACGAAGAAAACCGAAGCTGAACTCAA
CAAGCAGGAGGCGCATGAGCACAACGCGGCCGCAAGACAAATGGCGTCAACCGGTG
GAACTGCCGGCACTTACACCCACTCGGCCACCGGAGCCACCGGTCACCCAACCGGTG
GCCACCAGATGTCAGCCATGCCAGGTCATGGAACTGGACAGACAACGGAAGGTTATGT
GGCTGAGGGTGCGGCGGGATCTAAGCCTACTGGAATCAATACTGGGACGGGTCAGACC
ACAGCCCATAACACCCGTGCTGGTGGTGGCACCAATACTGGTGGGTTTGGTACTGGTG
ATACCTACACTTGAACCGGGTCTTACCCACTGTCAGTTTCTGGGTATGGGTCATGGGTC

TGTTTGGGGTCTGTTTTTAATGTTTAATGTAATAAATGTAGAAGTTGTACGGTTGGTGT
TTTTCTATCATCAGTTGAATAAAAAGAGCAAAATATT
>LsfLEA5-2
GGATACCACACTGCATTGCCATTATCCAGTGTTTCACAGAGCTAGAGAGTGCGATAGAA
CAAGAGATTAGAGAGCGAGAAATGGCATCAAACCAGAAGCAGAGAGAAGAACTTGA
CGCCAGGGCTAGGCAGGGAGAGACTGTGGTCCCTGGTGGTACTGGTGGCAAGAGCCT
CGAAGCCCAAGAGCATCTTGCTGAAGGTCCCTCTCCATCCCTATCTCTGTTTTAGTCAG
CATATGTCAATATTATTGATATCAGTACTATGCATTGAAGTTATATTTTATACGCAGAAAAT
CGCATGATCAATACATACTGACCTGAAAATTATTTTGTACCAGAGTATGTGCATGTTATAT
ATAGGCCGGTATTATTATTATTTATTTTTTCCTAAGATTCTTTTTATGTGAAGCTTTTTTTG
AGTTTGGGCATCTTGTCCTTGGTGATGAATTGAAGGAAGGAGCCGAGGAGGACAGAC
GAGGAGGGAGCAGCTGGGGACAGAAGGGTACCAAGAGATGGGCAGCAAAGGAGGAC
AGACGAGAAAGGAGCAGCTGGGGACCGAAGGGTACCAGGAGATGGGCCGCAAAGGT
GGACTAAGCACCACGGACCAGTCCGGTGGGGAACGCGCAGCCGAGCTAGGGGTCGAC
ATTGATGAGTCTAAGTTCAAGACTTCATCTGGCTAATGATGAGTTCAGAAACATCCAAC
GAGCTAGGCTTTGTGTCTGTATATATGTTGCGTTTTTCGGTAGTCGTCTTAAGTTGTGTT
TGGAGTACATCTTAGTAGGCAGTACTGCAATAGTAAGTTAGTAACAACTCGTAGCTGTT
GTCCAGAAAATCAGAAATCTACGTGTAGTTTGGTGCGAGGAGGGGCTTGCTCTTTGTG
TTTTGGTTTTTTGCTCTTTTTTGGGTCACGGTGCCTATTTTGTATGCAGTGAATGTCACT
GAATGGCAGATGAACCTCTAATGAATGACGACTCGATCCGGTTAACTTTCGGATTTGC
>LsfLEA2-53
ATATCATTATTCCACACACTTCTCTCTCTCTTCTTTCTCTCTCGTCATGGCAGATGCTAAA
CAATCCCACTTGAACGGTGCCTATTATGGCCCCTCAATTCCACCCACCAAATCCTACCA
CCGCCACGGCCGAGGTGGTGGCTGCGGCTGCGGCTGTTGCCTCCTCAGCTTCCTCTTC
AAACTCATCTTCTCCGCCATCATCATCGTCGGCCTAGCCGTCTTCGTCTTCTGGCTCATA
TTCCGTCCCACCCCCGTCAAGTTCCACGTCGTGGACGCCTCCCTCACCGAATTCAACC
TTACCAACAGCAGCACCCTCAACTACAACTTCGCCCTCAACATGACCATCCGAAATCC
CAACAAGTACATCGGCATATACTACGACCGCATCGAGGCCAACGCGTACTACGGGGGA
CAGAGGTTCAACACTGTTCCATTGACGCCGTTTATCAGGGTCACAAGAATACGAGTT
CGTTGAATCCCGTGTTTAATGGGCAGAGTTTGGTGGTGCTTGGGGCGTCAGAGCTTTC
CGCCTTTGATGCGGAGAAGAGTGCTGGGGTTTATAATATTGATGTGAAGCTGTATCTGA
GGATCAGGTTCAAGTTGCGTAAGATCAAGACTGGTCGGTTTAAGCCCAAGATTAAGTG
CGATTTGAAGGTTCCTTTGAGTTCCAATGGCGCTTCGGCTGGTACCTTTGAAGCTACCA
AGTGCGAGATCGATTTCTGATCGCCAGACCACCTTCTTCCTGTTTCATTTTGAGCCTGT
CTATCACATTTTTGTGATTCGTTCTTCTTCTTTTTTTCTTATTATTCTTTTGTTTATTAGCA
GTTATACTGATACATGATTATTAGCTTTATATTCATAGATCATATGATTTTTTTTTTTTTTAT
GGGATTTTATGTGTTACATTCGTGTATTTAAATAATGAATGAGCAGCTTGCTCCTTTCTTG
TTCT
>LsfLEA1-1
AGTGTAGAGTGTTTTCGAAAAGTTTGTTGTCACTGTTTTCTAAAAGCAAGTCCAAAAA
CACAAAAGCGAAGAAAGCGAAGATGCAATCCGCAAAGGAAGCTGTTTCCAACATGAC
GGCCTCGGCCAAAGCTGGCATGGATAAGACCAAGGCCACCGTGCAGCAGAAGGTTTT
ATACTTTTATTCTTCCTACTTAGTCTCTATATATTTGTCTCCTTCCTTAATTTGTATCAGAG
TATTTCTATTTTTCCACGTTTTCCTAAATTTAATTTTTCTTTTCAGAAAAGAAATTGCTT

TAATTTGTAAACTGTTTCTAAAATTTTCATCAAAACGGACAATTTTAAAAATTTCTTTAA
AAATCTTCGTATCTATTAATTTCGAAATTGAAAACAATATTTAATTAAATTTTAAGTAATG
TTATATTGTCCAAATTATTGGGGTGATGACGGTCCAATTGATCTGTGTTGGTTGCACAGG
TGGAGAAGATGACCGCACATGATCCAATCGAGAAAGATATGGCCACGCAGAGGAAGG
AAGCGAAAATAACTGAGGCAGAACTCAACAAGCAGGAGACCCGTGAGCATAACGCAG
CCTCGAAACAAGCGGGCAAGGCTGGGGTGGCCGGTGGAACAACCACCACCCACCACT
CGACCACCGGACTAGGCGGTTTCTCCACCGGAGGACATCCCCCCACCACCCACTCGAC
CACCGGGACAGGCGGTTACACCACCGGAGGGCATCCCACCACCACCCACTCGACCAC
CGAGGCTGCAGGTGGTTACCCTACTGGTGGACATCACATGTCAGCCATGCCCGGGGAG
GGGACGTGGCAGCCCACATGGCAGGAGGCTGAGGAACTGCTGGTGCCTAGAGGCACT
GGTACTGGAACTGGTGGGACCACTGGAAACACCCGTCTCGGTGGTGGTGCCACCACT
GGGAATGGGACTGGTGATGCGTACAGTTGAAATGGTCTTTGGGGGGTTTTTTCTGGTTT
TCTTTCGATTGTCAGTTTGTGGGCAAGGGATGGGTCTGTTTTTATGTAGAAGTTATTGG
ATTATGTTGGTGCATGTAATTTTCTGCAATGCAGTTTGATTGAATAAAAACTAGTACGAT
TATGTATT
>LsfLEA2-11
ACACCTTCAAATCATCTCTGAAAAGGAAAACAGAGCAAAAGAAAAAGGAAAAAAGA
AAAAAGAAAGAAATCTCTTTGATCTTTTTAAGAAAAGAAAATGGCTGAGAAGTACCAT
CATCTCACACCCGCAGCCCCAGGACAGGCCACCGCAAGAGACGAAGAATCAGAAATC
TTGCAATCCAAGGAGCTCCGCCGCAAGCGGAGGATTAAAATCGCTGCCTATGTTGTTG
CCTTTGTTGTGTTCCAATCCATAATCATCGCCATCTTTGCAGTCACTGTAATGAAGTACA
GAACGCCACAGTTCAGGTTGGGAAGGGTCACTGTGGAAAGCCTTACCTCCACCTCCA
CGCCTACCCCTTCATTCAACACGAGGTTCGTAGCACAATTTACTGTCAAGAATACCAAT
TTCGGCCCATACAAATATGACAACACCACCGTGGAGTTCTCATATGGGGGCATGGTGGT
AAGCCAGGTAGTGATTCCTAAAGGTAAGGCTGGCCTTCGTTCCACCAAGAAAAGGGAT
GTAGTGGTGGAATTGAATTCTGCTGCGTTGACGAGTACATCTAGCCTTGGTAGCGATAT
TAATTCTAGGATATTGACGCTGAGCAGCCAGGCTAGGTTGAATGGGAAAGTAGAATTG
ATGTTTGTGATGAAGAAGAAGAAATCTGCGCAGATGAACTGCACCATGACATTTAACT
TGGATGCACAGTCACTCCAGGGTTTGGAGTGCAATTGAGGACATAAATTACAGCTTGA
GTTGTATTTTTATATATGTTTTATTATTTTATTGTTACTTTTTCTTTTTCTTTTACCGTATTTT
TCTTCTCATCATTCTTTGTAAGATGAAAAGAATTTTTTTTTTGAAGAGTTTTTTTTTTAAT
GTGATACTTGGTGTTTTGAAAAACTTTAGTTCCTTAATTTAATTTAATTTAATTCAAAGTT
ACAT
>LsfLEA5-1
AACAGCGTCACAAATTTAGCTTCGACATTTCCACGTTCACATAGATAGCTAGAGAGTTT
GCAAGAAGAAAAGAAGGGGATGGCATCAGGACAGAGGCAGCAAAAGGAGGAACTTG
AGGCAAGGGCAAGGCAGGGAGAGACTGTAGTCCCTGGAGGAACCGGTGGTGGAAGC
CTGGAAGCCCAGGAACACCTTGCTGAAGGTTCTCTCTCACTTCATACTCGTATATATTTA
TATATATATATATAGTAGATAGAGTAATAATAAGGTGTTCAATTCATATTGTGATATTATATT
GATTTATTTTTTGTTGGAAAATGAAGGGAGGAGCAAGGGAGGGCAGACGAGGAAGGA
GCAGCTGGGGTCGGAAGGGTACCAGGAGATGGGTCGAAAGGGTGGGCTCAGCACGA
CGGACCAGTCCGGTGGAGAGCGAGCCGCACAGGAAGGCATCGAGATCGACGAGTCCA
AGTTCCGCAAAAGTGGCTGACTGTGTTCTTTGATGTGTTAATGGTGGTTGTTTGTTATC
TGTAGTATGGTTTTTTGAGTTTTCTAACTTAATACTCCAATAATAGTCTTGTGGAGGGAC

GAGGGCATTTGGCAGTGTTAACTCCATGAGTCTAGGTTTGTAGTGATCACTTCTAATCT
TCTATGATATAATAATTAATTGCATGTACGTTGTTAATTATTCAAAATTTATCTCATCTCG
TGTGTGGAGC
>LsfLEA2-21
ACCCAACTACATAAAAAACCCACCACTCCTAGCCAGCTTCTCCACCATATATACACCTC
CTTTCTCCTCAACCTTCCAAAACCCCTAGCCATGGCGGAGAAGGAGCAGGTCGACCGT
CTAAGCAGCTACGACGCCGATGACAAAGAGGCCCTCCTCCACCTGAAAAAAATCCGTC
GCGAAAAATGCATCAAATGCTGCAGTTGCGTAGCTGCCCTAATGCTAGTCCAAGCCATA
GTCATCATAATCCTAGCATTCACCGTCTTTCGCGTCAAAGACCCCATCATCAAAGTGAA
CGGCGTCACCGTCACTCGGATCGACCTAATCAACGGCACCATTCCCCGGCCCGGCACC
AACATGTCGGTCATCGCGGACGTATCGGTGAAAAACCCTAATGTGGCATCATTCAAAC
ATGGTAACACTACAAGTAGTCTTTACTATCGTGGCACTTTGGTTGGGGAGGCCAGGGGT
CCACCGGGTCAGTCCAAGGCTAGGAGGACTACGAGGATGAATATTACGGTTGATATTAT
CACGGACCGGCTTACGTCGAACCCGAATTTGAAGTCGGATGTGGGTTCTGGGTTGTTG
ACCATGAGCAGTTTTTCGAGGGTTGGTGGGAGGGTGAAGATAATGAAGATTATTAAGA
GAAATGTTATCGTGACCATGAATTGCACCACCACTGTCAACATTTCAACCCAGGCCATT
CTGAAACAGCAGTGTAAGCGGCATGTCAAACTCTAGGGTTATGTTATGTGATTGTAGTT
TTGTATCATGTATAAAGGGATTTTCTCCCTCCCTCTCCCTCTCTCCCACTTTTTTTTTTGC
CGGATCCACACACCTCTCCCTCTCTCCCTTTCCCCCTTCTCTCTTTTTTTTTTTTTTTTTT
TTTTGTTTTTTTCCTTTGTAGTTTCAATTTGAAATGGCATATTTTCACACT
>LsfLEA2-1
ATGGAAAATCCACCTAGACCAGTGACCGGATACCCCGTCACAGGCTACCCTCCGCCGC
AATCCGCCACGAATCCCCGCCCCAATGGTTACCCTTACAACGCCGCACCGCCATACTAC
AACCCTAACCCTTATATTTACGCTAACCCACGCGCCACCTTCCTCCGCCGCTTCATCGCC
ATCATGATCGGCTTATTCATCATCACCGGCACCATCGTCTTCATCGTATGGCTCGTCCTT
CGCCCTCGCATCCCCCAGGTCACCGTCGACTCTGTCTCCGTTACTAATTTCACCACCTC
CTCCTCCCAGGTAACCGGCAACTGGGACGTCCGGTTCTCTGTCAGAAACCCTAACAAG
AAGATGAGCATCTACTACGACAGCATCGTTTCCTCTCTCTTCTACGAAGCGGCATTCCT
CTCGCGGACGACGCTCCCGCCGTTTGATCAGGATACAAGGAATCTGACGACTGTGAGG
GCGGAGTTCGCCGCCGCGGGGGTCTACGTTGACGGGAGGTTGGTGAATGAAATTAATA
GTGAAAGGGCACGTGGGGCTGTGGGTTTTGATGTGACGGTGCAGGCTAGGGTTAGGTT
CAGTGCTGGGGCTTGGAAAGCTAGAAGGCGGTTCCTGAGAGTTTTATGTGAAGATGTG
GCAGTTGGGTTATCATCGAATGGTGCTTCGGGAACGTTGGTGGGTGGACCAAAGGAAT
GCAGGGTTGGTCTGTGA
>LsfLEA2-17
ATGGCGGCATTGCTGGACAAGGCGAAGAACTTCATGGTGGAGAAGGTGGCACACGTA
AAGAAGCCAGAGGCAAGCGTGGAGGACGTGGATCTCAAGGACGTGAGCCGTGAGTG
TATCGACTACAATGCTAAGGTAGCTGTGAAGAACCCCTACGGACATACTCTGGCCATTT
GTGAGATCACTTACACCTTCAAAAGTGCTGGCAGGTTTGAACCTTTCCCATTCTTCTTT
TTCTTTTTTCTTTCTTTTTATTTTTTCTTTTTATTTTTTTTTTTTGGATAAAACAAGGGTTG
GAAATAAAATATATTGAATATAAAAATAAATAATACCACTTTGATATTAAGGACTTTAATC
AATAGTCGGTGGATCTAGTGTATGACATAGTGGGGATGAATCAAAAAAAAAAAAAATC
TTACTTGACCAACCATTATTTTTTTAACCTTTTTTAATGAAGATCGAGAATGACTCATTT
CAATTCATGGAAAAATAAGAATGAAAACCAAGTGGAAGTTTTAGTTCTAATGGATCGA

GGGCACTCAATTATATCGATTTGTCGGCAATCTGAAATTTTCGTTATAATAAATTATTGCC
ATTTGCAGGGTAGTGAAATTTAATATATTAAAATATAAAAAGTGCTTTAATACTTAAAATT
ATATATGCATTTTTATATAAAAAAAATTTGCTAATAATGTGGGTATCGATGTGGCAGACAT
TGAGTTTCTTCTTATGAGGTTAACTTATCTTACATGGAAGACTCACTCGAAATTAAGAAT
TCAAACACGTGGTATGTGGTATCTTTGCAAATTCTTTTTGATTATACAAAACCATATAA
CTTGGAATAAAGAATAATTTCTGAATCTTAAATTTGTGCTTATTTGTAATATATTAATTGG
TGTGATTTGTGTCTGATGTGGTGTGTGGATGTGGGTCAGGGTTATTGCATCAGGAAAAG
TACCAGACCCAGGGTCATTGAAGGCAAACGACATAACGATGCTGGACGTGGGGTTGA
AGGTGCCACACAACGTATTGGTGACCTTGGTGAAGGACATAGGTGCAGATTGGGACAT
AGACTATGAGTTGGAAGTAGGTCTCACTATTGATCTTCCAATCATCGGAAATTTCACCAT
TCCCATTTCTAAGAAGGGCCAGATGAAGCTTCCCACCCTGACTGACATTTTTTAA
>LsfLEA2-42
ATGGAAGATCGTAGGGATACAGTGATAGGCTACCCAGTGGAGTTCTTTCGTGGTCAATC
TAATTATCAAGTCTCCCACTATGCCACAACCAGTGCCGCCGCCGCCTCTACTTCCGCCT
CTGCCACCAACAACAACGTTAACACCGCCCGCGCTCATGTTGTGCAACCACCAACCGT
TAGCCATCATCATCAGCAGAGGCCAGGCAAATCGAAATTATCAAACTCTTGTCTCGCTC
TGATAATAGCGAGTATCAGTGTTGCGGTGATGTTTGTTATCTATGCTATCGTAAGTTTCCT
TAATGCTTTCGAATTCCCTGAGTTCACACTCAACTCGGTCTCCATCTCTCCCTTCAACCT
CTCCGTTCACTCTCACACCACCACCACCAACTGGAACGTCAGTTTCTACATCAGAAAC
CCTAACAGGAGGATGAGTTTCTCTTACGACATGGTCCAGGCTTCGCTTTTCTACAAAGA
TGCGCAATTCTTATCGCCAGGAACCATCCCTCCATTCTCTCAGGGCACCGAGGATTCCA
CGTCGGTGCAGGCGAAACTTGCTGCCTCGTCCGTGTACGTCGACGACGGCTTGAGGA
AAGCCATTCTTGATTGTTGGAGTAGACGCGGGGCAGCGGTGGATTTGAACGTGAAAGT
GCAGGCTCTGATTAGGTTTGACCCTGGGACAAATTCGGAATCAAGGGGTTCAGTGTGG
GTGTTTTGCAAGGACGTGAAGACGGAATTTTTGTCGAATTCGACATTGGGGACAATGG
TTGGGGGATCAATGAAATGCAAGGTTGGTGTAAATACATGACCATCAATATCAATGCAA
TCTTTTACACTACGATTTGGGATTAATCTAAGGGGTTATGTAGGTTTTGCAAAGATTAAT
ACATAATTAGCTAGATAGATTAAATTAACTGGCTATTAATGTGTTAATGATAGTTGTTGCT
GGGTTTTCTTCATGAATGGTTGCAGACTTGTGATGACTTCCAATTAAATTGTCTTAGTAT
TTTTTGTTTTTATTGTTTCATTTATTTTTGTTGAATAACTGTGCTCAATTGTTCTTGATCTA
CATGATGTTTTTGTAATGGACATCCATTGATATAGGAACTTAGTGCAATTGATTTTTGAG
ACCCGCCTCAGTCTCTCCCTTCAACCTTTCCACCAACTCTCAAATAATCACCATCTTGA
ACAAATCAAATACCAACAACTAATTGAAAGTGGCACTTTACGGCCATGTGAGATGCCC
CATGAAAAAAAAGCATTGATATCCATGTTTTGA
>LsfLEA2-31
ATGGCAGAACCTGTAAAACCGGTTCTACAAAAACCACCCGGATACCGAGACCCGAAC
GTTCCAGTTCAGCCCGCCCCGAGACCGCCAGTTCGGAAACCGGCTCTCCCACCTTCCT
TCCAACCAAAGAAGAAACGCAGGAGCTGTTGTCGTATTTTCTGCTGCTCCCTTTGCATT
CTCACATTCATTCTCCTACTCATCCTTGCTGTGTCAATTGGGATTTTCTACATCTGGTTCG
ACCCGAAACTCCCGGTTTTTCACCTCCAGTCATTCAAGGTCGAACGGTTTAACGTCTCC
GTCACATCTGACGGCACATTTTTGGATTCCCGAACGGTCGTTAGGGTCGAGGTCAAGA
ACCCTAACAGCAAGTTGACGGTGTATTACGGTGGGACCCGAGTGAAGGTGACCGTTGG
AGAGGATGACACTGAGTTAGGAACAGCGAGTTTGAAGGGGTTCACTCAGGGGAAGAA
GAACACGACGAGTTTGAAGTTCACGACTCGGGTGAAGAGTGAGTGGGTGGATGACAG

TGTTGGGAAGAAGCTGAAGGCTCGATACCGAAGCAGAGCGCTGGTTGTTTTCGCGGA
GGTTCGGACGAGGGTTGGGTTAGGTGTGGGGAGGTGGAGGATGGGGACGGTGGGGGT
GAATGTTTTGTGTGGGGACATGACTCTGAAGAAGCTTGCTGCCGGTGCCATGCCTAAA
TGTACCATTAATTTGCTCAAATGGTAAGAAGCTTAATTTTATTAACTTTCCTGTGTTTTCC
AATGACCTAATTGATTCTTTATTTTATTTTATTTTATTTTAATTTTTCATAAACCATTAGATT
ATCTTAGGTCTTTAGAATTTTAAGTTTTTAGTTCAAACTAATCTCCTAATGACTCGTGTC
AGACGCTCTATTACATGCAATAATAGTTATAAATAATAGAAATGATGACTTCTGAACGTC
CGCTTGATCAACTCTACTAGGGCTATGTTAAGAGATGCTCACATACGAAAGAGATTATT
GGTTGCCTTATGAGGTTTTGGATTAGGTTAGGGATCATTTTAACACAAATAAAATCATGG
ATATTGTCAAATAAATATGGAATGTGATATGATCTTATGTTAATATCTCGGGGTTTATGTAT
CATGATCTTGACCCACTGATGGGCTGACTATTTTGATTGTCTTCGTTGCTTTAATTTGGC
TGCAGGATCAACATACATTGA
>LsfLEA2-19
ATGGAAGGCCAAAAGCCACCGGTGATCGGCTACCCGCTGCAGTTCTCTCATGGGCAGT
CTCCTAATCCACTCTCTGACAACCCTACGGCGCAGCCTCCCCATTCCACCACTACCACC
ACCACTGCCGCCGCTCATGTTGTGCAGCTACCACCCATCGTCGGTCATCAAGATCAGCC
ACGACCACGGCGACGCCGATCACAAATCTGCAGTGGTACTAATTGCGGCACTCTAGCA
GCAATATTGGCTTTGTTGGCCTTCGCGGCCATCGGGGCGTTCTTGGGTAACAAATATTC
AATTAAATCCCCCGTGTTCCGACTCAACTCGGCCTCCCTCTCTTCCTTCAACCTCTTCGT
CGACTCTCAAATAACCGCCAACTGGAACGTGACCTTCTACGTCAGAAACCCTAACAGA
TGGGCGAGCATCGCTTACGACGTGACTCAGGCTTCGGTTTTCTACGAAGATCAACTCC
TCTCGGCCGGAACTATCAGTCCTTTCCAGCAGGGTAGGAGGGAGCAGACGTTGGTGAC
GACGGCGAGTCTTGATGCCAAATCGGTGCACGTCGACGACTGGGTGAAGACCTTTATT
CTTGATAATTTGACCAATGCAGGTGGAGTGCTGGATTTTAATGTGAAGGTGGAGACTTT
GGTTAGGCTTCACCCAGGAACATTATCGGAGGCAAGGGGTAGGGTGACGGTCTCTTGT
AACGTAAAGATTCAATTCTCGCCGGATACAACGTCGGGGACAATGGTTGATGGACCAA
GGGAGTGCAAGACTCATTTAGATGACCATGGACATGTATTCTGA
>LsfLEA2-32
ATGGCGGATTCTGCAATACGCAAAACCGAGGACAGCCCGCCATCATCAAAGCCTTCCC
CTAATCCGACATCAAAGCCCGTTCGCCACGTTGTCTTCTCAGAAATCCCTTGTAGACCT
CACAAGCCCGACAAGGAACGGCGAAGAGGCCGCTGCTGTTCGGGTTGCTTCGTTGTC
TGTTGTGCATGGGGCTGCATGATTGGCTTCGCTCTCGTTCTCGTCCTCCTCGTCGTTGC
CGCCATTTTCATCGCCTTCCTCCACTCTGAGATGCCAGAAGTCCGCGTAGGGAGGGTGT
CCATCCCTTCGTTAGATGTGGTGACGGCCTCATCGCAACAGGCGCTCTTGTCTGCTGAT
GTGGATATTGTCGTCGAGGTCTCCAACAAAAATGACAGAGCTAAGCTCTCTTACGGCA
CCATGACGGTGGACGTTTCGTCGGAAAAGATCCGCTTGGGTCAGACTAAAGTTGTTGC
GTTTTCTCAGAAGCCTAAAAATTCGACGGAATTGAAAATACACACCAAGGTTACCCGG
TCGGCAGTGGACAAAGAGGATGGGGCATTCTTGAAATCAAATGTCAAAAACGGGGAG
ATGGTTTTTGATGTTGTTTTAAGCGGAACCATAGGTGTGGGCGTGGGTAGGTTCACCAT
AAATGGCTTGCCCGTCACCATGGCATGCCATGGCATCCAGCAATCTCAGGTTGATATCG
GACAGGAGCCCAAATGCAGTGTTAAAATGTTCTCCTTCAGGTTACATACTTTCCTGTTT
ATATTAATTATTAATTATTAA
>LsfLEA2-18
ATCACTATCTCTTTATATATATGTACATCTACACACACATAGTCTATTGATTGCCTTGTCTC

TCCAAGCTGTTGGAGACTCATTCTCACACACTCTATCTTATTGCACCTGCCCAAAGGGA
AAGAACCAAAAGAAGAAACGCACCGGCATTCTTTAGAGGAAAGTTAGATCCTACATCC
ATCTCGATCATCGCGCCATTCTTCTAAAGCAATCACGGTGCTGCAGATTGCCTAGTAAA
AATTCTATCTCTCTTTCATTTCTTTTGCCTTCAAGCGTTCATTCTGTTTAGTTTTATTCTGC
TCCTCTCCTCTCATGCAGTTTGCTTTATTCAGTCGATAGGATGTTCCATTTTTGTTCATTA
CTAAATGATCAATTAACTTGGTAATGATGAATTAACAATCAGGTCTATTGATCAGGGTTG
TTCCTCGTGCATGACAATTGTATGCGGAGTTGCATGTTAACTCTCTCTGTCTAGTTTTCT
TGTGTAATCGTCTTTTATTTATTTTCATTCGGTATATATTTAGAGTGACTAACACTGTTTTG
GCCGGGGGCCGGAGTAATTAGGAATTCATATATATAAAATTTCCAATTCCACTGTTTGGA
ATTAATGGAAAGATTTAGAATGACTTAACACTGTTTTAAAAGATATTTGAGGGGGTTTC
ATTAATTTTTTTACCTTGTAAGTATATATGATATGATTAAACAATTGTTTTGCAATTTCTTC
TTTTATTTATTTATTTTCAGTTTTTCATTGACACCGAAACAATAAAATATTAGCCAATGGC
AAGTGCTACAACGTGCACCCCATATATGGCTCTTCGATTCTTTCATGGATATCCGATACA
CACTTTTGAACTACCCTACCATGCCAATAGGTAAATAATACCCTTCAAAGTGACTAATAT
TTGTGCAAAAACTTATTACTCCCTCCCTCCTATTTTATTTGATCATTTTGATTATCAACAC
CTATAGAGGCAATTGCCATGTCACCTGTCTTATAGTACCTTTCCAAATTTGCCCTCCTATA
GAAGGGAGGTTTAATTTTGTAGGATAAAAAATTAACTAAAATAAAAGGGTCTTTTAGGA
AATAACATCCCTTTTTGGTTTGACTTTTAAAAATTATCAAAATTTTGGGATATTCCAAAA
TAGAAAGATGATCAAATAAAATGAGACTGAGCGAGTAAAATTATTTTCATTTGAGAAAT
GCCAGTTAGCCCTAAACTTTGTCCTACGAATATTGTCTTACTAAAAGAGCAGGGGGTAA
AATTGTGGGACCCATTAATATGGTTAAATCCTAAAAATTGAAATAAAACTAAATTCACAT
GATAAGATAAGATTGTAGGACAAAGTGTAGGGCTAACTAACATTTCTCAGCCTTTATCA
TTTTTGGCAACGAACTTCCCTCTTTCTTTTTCTTGTTTTATTTAATTATTTTCTCTCCTTTT
TGGAAGTAGTAAAATATATATATATAAATATATTGTTGAATGGGTATTTGGTGATTTTGAC
ACTTTAACGCATCCTTTGAAGTTTTCTTCTTTAGGTCATTTAAAAACTTGACTTGTGCCA
ATGAATCCACCAAAAATTTACTAATATCAAACATTGAATATGACTTTCGTTCCTGCAAAA
CCAACCTTTGTATCAGCCCTCTATCAATCAATACAAATAAATTATCACAATCACGATTAG
AGAACACCATTAATATTTTGGACTAACTAATTTGTTAGTCTAATCGAGTGTGAAGCTTGA
TATCATGTGTTGAAGTAAATCATTTGTAAAAGACTAATAGTCATTAGATTTTATACATTTG
ACGATTTAAATGTCACACCAATCATTTTTTCAATGACAACATACACTTAAATACGTGAGC
ACATGTGTACCTAGTATGTAAATGTTAAACATTTATATAAACTTGTGGCTGCATGGCTAC
ATGCATGGTTGAGTGTCTCTCGTTGCTTTTGCATTGTCTTGCAAATGGTTTGGAAATAA
AAATGGGTTTTATCCCCGACTATGGTACATGTTAAGATATATAAACACTAAGTGTATAAG
AAAATATATTTGCACAAGTTTCTATATATCTTTTCATTTTTTTATACTATTTGCGTCATTTC
AAATTTTTCTATACTATTTGCATCATTTCTAATATATATATATATACATCATTTTCAATGTAA
CTTCACTTTTTATTTTTTCATTAAATATATTGTAACATATGCTCTAATGCATGCATGTATTA
GTAGGACGGAATAAAAATTTAACCTATACATAAACGAACAAGTAAATGTAGGAACATG
GGAGTTGACCAGATGATTTCTAATTTACAATTAGGATTTGGTTAATTAGATTTAACGAGT
GAGCTTATCAACACACAGACTGGATCCAAAAGGCCGATGCAATCACGCCTAGACTTTT
AGTGATGCCTCACCAGAAAAATTTGAACATTTCTTATTTCTTCTAAGCATACGCCAACC
TGTTCACGTAACTATAAAAAGTCTATAACCAAGGGTCCCACTCCCACATCTGTTGTCCC
CACATGCTTACATGCTCAGTTCACACAAAAATCAAACCACATGCGGCGCGGGCCCCAC
ACGCTTTCCCCCGATGGCGTGGGTGTGGAAATTTTCCCACTTCCCAGCCGAGAACCGA
GTCCATATTTGCCCGTCAAGCGCGTGTAAATCACACACGCGGTTACAGTTTCATACTAA

AACCAAGCATCGCCATTTTGTGGTTTCTTCACATTCGCTTCTGTATTTTATATTCTCATCT
CAATACAATACAAATTATCTGACATTGAACTAGTACACTCTTTTGTAGTACAGATTTCGT
GACTTGGAGTTTCGCATTCTACCTCAATGGCCGACCGAGTTCACCCCGCCGACTCACC
ATCATCCGACACGGCGTCGTCTCACAACCCCGACGCCCCTTCCAAGCCCACCCCTCCC
CAGCCGGAAAAGCCAGTCCCTCCGATTGGAACGTACGTCATCCAGATTCCCAAGGACC
AAATCTACCGAGTCCCTCCGCCGGAAAACGCCCGCCGCTTTGAGAAGCTCACTCGCCG
AAAGACCCGCCGGAGCCCCTGCTGCTGCTGCCTCTGCTGGCTCCTCTCCTTCATTGCTC
TTCTCGTCGTCCTCATCGCCATATCCGCCGGCATTTTCTACCTCGTCGTCCGCCCGAAAT
CGCCGAAATACTCCGTCGACAACGTCTCCATCACAGGCTTCAACATCTCCTCCTCGTTG
GCGATCTCGCCGGAATTCGACGTCACCGTCAGAGCCGAAAACCCTAACAAGAAGATC
AGTATCTACTACGAGAAAGACAGCTCCGTTAACATCTATTACTCGGACGTCAAGTTGTG
TAGCGGCGTTTTGCCGGTGTTTTATCAGCCGTCAAAGAACGTAACGGTTTTCCAAACG
GCGTTAAGTGGGTCGGGTATAGTGCTGGGGAGTACGGTCCATGAGACGTTGGTGAATG
CGCAGAACCAAGGGCAGATCCCGTTTAAGCTGAAATTGAAAGCCCCGGTGAGGATCA
AGGTGGGTTCCGTTAAGACGTGGACGATTACCGTTAAGGTTTCGTGTGATATAACGGTG
AATAAGTTAACGGCGGACGCGCAGATCCTTTCTAAGGACTGCGACTTTGGCGTGAAAC
TTTGGTAGGACGGCACATGTTTTGATTGGTCGTAGAATAGTTCATATACTTTCATATGGA
TTTGGTTGGGGAGTAGTGTTCGGTACATCTCTTACTACTTCTGTTTGTCATACAGAGTAC
AGAATCATTGATTGTTATACTTCGTATACAGAGTATACATACATTTCAGAACATTATTCAT
ATTTTATTTTGA
>LsfLEA2-34
ATGAGTGTGAAGGACTGCGGCCACCACCACGACGAGAGGCGAAGGCGCAACTGGCG
AATATTGGCTTGCTTGGTCTTCTTGCTCTTTCTCATCCTCTTCACAATCTTCCTCGTATGG
ATCATCCTGCGGCCCGCAAAGCCCCGCTTCATCCTCCAAGACGCGACCGTCTACTCCTT
CAACGTCTCCTCTCCCAACTTCCTCACCTCCAACATTCAGGTCACCGTCTTATCACGAA
ACCCTAACAACCTCATCGGCGTCTACTACGAGAAACTCGACATATACGCCTCGTATCGA
AACCAACAGATTACGCTCCCAACTTCATTGCCTACGACCTACCAGGGTCACAAGGACA
TTGTGGTTTGGTCTCCGTTCGTGTACGGCAATAATATACCTGTGGCTCCCTATCTGGCCG
TTGCTTTGAGTCAAGATCAGATGGCTGGGATGGTGCTGGTCAATATCAAGATCAATGGA
CGGGTCAAATGGAAGGTGGGCACGTGGATCTCGTCGAGGTATCATCTGTATGTCAACT
GCCCTGCTTATATAGCGTTTGGTAACAGGAATACGGGGATTCCGGTTGGACCGGCCATG
AAGTTTCAGCTGGCGCAAGGATGCAGTGTGGATGTTGGCAAGTGA
>LsfLEA2-33
ATGGCAGAAGAAAAGCCCACACAAGAAGAACAAATCCAGCAGCCCCAAACCAACAA
AATGAACCCTGGCTGCGGCTGCGGAGGTAAGACCCACCCATCTCACCACCTACGCCGT
GGCATCTGCACATGCATCGCCATCTTTCTCCTCTTGGCAGGCGTCACAGCCCTCACCGT
ATGGTTAGTGTATCGCCCCCACAAGCCCAAGTTCGCGGTGGTGGGCGCCGCCATCTAC
GATCTGAACACCACGTCCCCACCTCTCATCTCCACCACCATGCAGTTCACCATCGTCAC
AAGAAATCCCAACAAGAGAGTTTCCATCTACTACGACCAGCTGTCGGCGTTCATGTCG
TATCGGAACCAGGCGATCACGCCACAGACGATGCTGCCGCCTCTGTATCACCGGAAGC
ACAGCACGGTGGCGCTGTCGCCGATGCTGGGGGGAGGGATGGTGCCGGTGTCCGTGG
AGGTGACGAATGGGTTGGCGATGGACGAGGCGTATGGGGTGGTGGCGCTGAGACTGG
TGCTGTTGGGGAGGTTGAGATGGAAGGCGGGAGCCATCAGGACAGGGCGGTATGGGG
TGTACGTGAAATGTGATATGTTGGTGGGTGTGAAGAAAGGCGTTACGGGTCAGGTGCC

GTTGCTTGGAGCTCCCGAGTGCAAAGTTGATATCTGA
>LsfLEA2-37
ATATCATTCTCTCATTTTTCTCTGAATTCTCTCTCTCGTAGACACAAGGTTTGTTATGGCG
GAATTGCTGGAGAAGGCGAAGAACTTTATGGCGGAGAAGGTGGCAAACATAAAGAAG
CCAGAGGCAAGCGTGGAGGACGTGGATCTCAAGGACGTGAGCCGTGAGTGTATCACC
TACAATGCTAAGGTCGCTGTGAAGAACCCCTACGGACATGCTCTGCCCATTTGTGAGAT
CTCTTACACCTTCAAAAGTTCTGGCAGGTTTGACCCTTTCTCATTCTTTCTTTCTTTCTT
TTTGTCCTTTAGGTAAAACACGGGTTGGAAATAAAATAAATTGAATATAAAAATAAATA
ATACCTCTTTGATATTAACGACTTTAATCAAGAATCAGTAGATCTAGTGTATGATGAGTG
ATTTTATTTTTCTCTTAATTCTGACAGATTGGATTGAAAACAAAGTTTGAATGAGTTCTT
CTAACACGGGGATGAATCAGAAAAGAAATCTTTACTTGCCCAATCATTTTTTATTTTATT
TTTTATCTTTTAATGGAGAGAATGACTCATTTCAATTCATGGAAAGAATAAGAATGAAA
ACCAAGTGAAAGTTTTAGTTCTAATGGATCGAGGGCACTCAATTATATCGATTTGTGGG
CAATCTGATTCAAATCCAATAATATAAGATCACCAAGTGTTGGAGAGTTCCTCTTGTAG
CATCTTTTTCTTACTAGATTTGGCAATTGGATCTACAGTATATCGTTTAGGACGTTGATAA
TTTTAAGATCTTATTATGGTGGTTGAGGGTACTATGCCTTAATTTGTTTCACCAATTATCT
TTTTCTTACTAGATTTGGCAATATTTTGAATGTTGATTAAATTGATTTCTTTATTTTTTAAC
ATTGATCATGTTTTTCATTGATATTCTCCACCAAGAACTTAAACATCGATATCAATAGATA
TATTATTGAAAATCTCGCTATTGATTTAAAGATATTATAGAAATTATTAATTAAATAGCTTT
TGAAATCTGTCCACAACACTAAAAAAATGTTGGATCCCATATGTAGGAAAATGCTTTTTG
ACATCTCTTAACAATCTCCTAAACGAATCTTCTATAATGTGGTATGAAAATAAATTGATA
CTAATATTATTAGGTTCTACTACTCCATCTCTACTCTTACGTCACATCATAGAAGGCTATT
TTGGTAACATGTTAGGGGTTGAATAGCATTACCCATAAACTTTTCAACATTGGATAAAAT
CTATACATGTCATGGTTGGATGTAACCTAGCTTCTTGCAGTCTTGATACAATTTTATTTTT
TTTTAGGCAAATTATAAGATCTTAAAATTATCAACGTTGACTCTTCCATTTGGTTGCACT
TATTTTAAAATCTCTTTTGCTTTTGAAAGCAAAAAGTTAAAAAGCAAAAATCACTATTT
GGTTAGAGGAACTAAAAAGGACTTTTAAAAAGTAAAAAGCAAAAAAGCAAAATATTT
GATTTTAATCAAGAGTAACGAAGATTTGCTTTTAAAAATTATTTTTATCTTTCGAACGAG
CTTGACCTTAACTAAAAAAATTCGTGATCGAGAGAGGTTGAGCTTATTCTAGACTTCCG
TTTAGGCAACGATGATTCGGTAGAATTTCTCATTACATAAAAAAATTACCAGTGAAATC
AATTTTACCAACGTGTTTTTGTTTTTTTTGCTTCTACTTAAAAACAAAAAGCAAGAAAG
TAAAAAATGACTTTTTCTACTTTAAAAAAAAAAGATATTTTAAAAAGTGCAACCAAATG
GTGCCTGATTCACTTGTTATTTTGAAAATGGCCGGTTCTAATAACTTGTTTATGAGAGAA
ATTAAACCAATCAGAAATTTTCGTTATAATAAATTATTGCCATTTGCAGAGTAGTGAAAT
TTAATATTAAAATATAAAAGTGCTATTGATATATATATATATATATATATATATACATTTTTTA
AAAAAATTTCTTTTTGCTAATAATGTGTGTATTGATGTGGCACACATTTAGGACACATTG
TGTGTCCAAATAGAAATTAACAATTCAACATATCTTACATGGTATTATAGTATTGATTGAA
GACACAAATCCATCGACTTGATGTTGTAGATACAATTGTAGAAATTCATGAAATACTATT
ATTTTTAATTTCTACTCTTATTTGTGATAATGACCTTTAATTTTTTTTATTTTGCATAACTT
GTTAAGTGGATAATCTCAGGTTCAAGCTTATGGAAAAGCATTGATACTCATTTTGTATCA
ATTTATTGATATTGGGTTAGGGATAAGAATAGGTTATATTGATTTATAATTTTTTTTTTTG
AAATGTCCTTAGCGAATGAGTGAGTCTTTCATGTAATTGTGACACGGCCACATGGTGTC
ACGGACCAATGGGAGTGAAGCAAAGTGGAATTGTGGGGCTAAAAAGTACTTTAATTAT
CCCACTTTGTTTCAATCTCATTAATTAGCTTATGATACCACATCAACAGTATCATTGTTAC

ATATAAGTTTTTCTCCTTAGTGAACATGAAATTCATTTGGACGAAAAAAAATTAAGTTA
GTGTTATTCGTACTTTTCAAAAGTCAACCTAAGAACATCTTGAAGAGGACTAGTTATTT
TTTCTCTACTTTAAAGAGTTTAATAAGAACTGTTTCTTATTTAGCTAAGAGAACTAAGAG
TGCTCTCCAATAGGATTAGTTAAAAGAGAGAACTTCCTATTCAAGTGAAGGTTCTAACA
AAGCTACCTATTTTTTTCTCATAGGACTAGTTATTTTTTCTCTATTTTTGCTAAGAGAGTC
AAAAGGGTTAGTAACATTTGGAGAGCCTTAAAGGGCCTAGATATTTTAGAGAGCTGGA
TGCATAGTTATTAAACTCAGATCGGTCATCGACCCAGTCTGGAGATCGGGTCACTGGTT
CAACTGTCGAGTCACCGGTTGAACCGGTCATATAATTAAAATAATACCAAAAAATAAAA
TGTATACATTTACACTTTAAATAATATGTGTTGTGAAGTTTTTCATATAAGCACTAAGTGG
TCTAGTGGTTAAGAACTCCTTTGGTCGTGTACATTCCCGTTTCTACTCCCTATATGCGCA
AAATGTTAGAAATTTTAAGTATTAGTACCAGTCTAACCTGACCGGTTTGCATCCAGATC
ACATAAAAAACTGGTGGTTTAAACCGGTTTAATTCGGTTCGTCCGGTTCATATGCATTTT
CAATCCAATTGAAAAATCGGACCGGTCTGGTGTCCGATTCACTGGTCCGGTCTGGGTTT
AGTAACACTATGTCATTACTCTCTACTTTAGCTCAAAAGTTAAAATAACTAACTTTTTGG
AGATGCTCTAAGATGTTTAATGTGGGAAAGAATTTGGTGTTGGTTTTAATCTAAGCCGT
TGGATAGGGTTTTTGATGGCTCGAATTAAAAGATAAAAAATGTTACCCGTTGAGGTCAT
TTTGATTATTTCAATAGAAATTTTTTGATCTTTTAATTTGAGCCGTCAAAATCCTCATCTA
ATGGCTTCAATTAACCTAGCACGGTGATAAGTTTTTTGAACCCGGCAAATGTTGCCTAT
TCACTCGCACAGTGGAGGTGGGGTGTATGAGGCTTTTTAAGGAGTGCATTTATTATAAA
TACCCTAAAAATTAGGAGTTAGTGGCACTACTCTTCAAATATAGTGTCACTACACCATCC
AAGCGCTATTTGCATACTCTTCACGTGCGCAACAAAAAATTTGATTTGATTTTATTTTATTT
TATGGGAAAAAGTCTCAAATTATTATTATTATTTTTTTTTAAAGGTATCAAATGACAAGTC
ATTCTCCATTTCCATTGTATTTTCTTGTCATGATTTGTCTGTCATACTCGCAAATTCTTTCT
TAATTATACAAAACCATATAATTTGGAATGAAGAATAATTTTTGAATCTTAAATTTGTGCT
TATTTGTAATATATTAATTGGTGTGATTTGTGTCTGATGTGGTGTGTGGATGTGGGTCAG
GGTTATTGCATCAGGAACAATACCAGATCCAGGGTCATTGAAGGCAAACGACGTAACG
ATGCTGGATGTGGGGTTGAAGGTGCCACACAATGTATTGGTGACCTTGGTGAAGGACA
TTGGTGCCGATTGGGACATAGACTATGAATTGGAAGTGGGTCTCACTATTGATCTTCCA
ATCATCGGAAATTTCACCATTCCCCTTTCCAAGAAGGGCGAGATCAAGCTTCCCACCCT
GTCTGACATCTTTTAAGCCCATCTTTTTTTCTCTCTTTAGCTAAGTTTGCTTGGACTACTT
ATATTTTGTGTTTGGATATGAAATACGTTACTTAACTTTTATGAATGTCCCTTTTTTTTCAT
TTATTTCTTTTGACAATTGTTTGGGTTTCTATTTTTTATTGTTAGTTAGTATTGTTACTCTA
TTTTTTATTGACTTGGGTATTCATCCCAACTGTAGACACTCATTTTGTCCTTATATTTGAT
GGTGAGTTCCCGA
>LsfLEA2-28
ATGGAAGACCAAAAGACACTGGCGATCGGCTACCCACTGCAGTTCGGCTACCCTACAA
ATACAAATTCTGCCTCCACCACCACCACCACCACCGTCGCCACGAATAATAACAACAA
CGCCGTTGACGCTCATGTTGTGCAATTACCGCCCGTGGTTCCTTATCCTGAGCCGCCAT
GCCGATCGCGAAACCCCCACCGTTATTGCAATATAGGAGCAACATTGACCATGATGGCT
ATGTTGGCCTTGATCATCACCTTTGAGGTCTATCATAACAAATATTCAACTAATTCCCCC
GTGTTCCGACTCGACTCAGCCTCCGTCTCTCCCTTCAATCTCTCTATCGACTCTCAAATA
AACGGCAACTGGAACATCAGTTTCTACGTCAGAAACCCTAATAGATGGACCAGCATTG
CTTACGACGTGATTTGGGCTTTGGTTTTCTACCAAGATCAGCTTCTCTCGGATGGAACT
ACCAGTCCTTTCCAGCAGGGTCGGAGGGGCCAGACGGTGGTAACGGCGAGTCTTGCG

GCCAAATCGGTGCACGTTGACGACTTGGTGAGGAACTTTATTTTGATGATTTGACAAG
TACAAGTGGAGTGCTGGATTTTGATCTGAAGGTGGAGGCTTTGGTTAGGCGTCACCCT
GGAACACTGTCGGAGGCAAAGGGTGGAGTGAGGGTTTCTTGTAACGATGTAAAAATC
CGATTCTCGTTTAATACAGGGACAATGGTTGGTGGATCAAGGAAGTGCAAGACTTATCT
GGATGACGACGATGAACGTGGAGGCTGA
>LsfLEA2-15
ATGGCCAATCCGGCGGCGCCACCCTCACAAACACCTCTGCCGCCGCGACGCTCAAAAT
TAGTTAGATGCATTGCCCTAATCTTGCTAGCCTTGATTGTCCTCGTGGGTCTAGTTGTGC
TCATCATATGGCTAGTGGTCAAGCCAAAACGCATAGTGTACACCGTCGAAGCTGCCTCA
CTCAGTGGTTTCAACTTGACCCATGACCACCTCAGTGCCAACTTTGATTTTATGTTGAG
GTCTGTCAATCCAAACCATAAGGTGTCTATCTACTATGACTCAATGGAGGTAATGGTGT
CTTACTATGATAATATATTAGCATTTGCTGAGGTCCAACCCTTCTATCAGCCCCGTCAAA
ATGTGACTCAATTGGAAGTGAAGCCCGTGGCCAAATCTGCGGCACTAATGGAGTCTGC
TTCTAGGGACCTTAGGCTTGAAAAATCGTCGGGTGGGGTTGAATTGGACATTAGGGTG
AAGGCAAGGATACGGCTCAAGATGGGGGCGTGGAAGTCGCGACGTCGGACCCTTAAG
GTTTTTTGTCCACATGTGTTGGTTCACTTTTCTTCGGCGAAGAAATTCCAGAGGGCCCG
CTGTGATGTAGATCTTTAA
>LsfLEA2-8
ATGGCATCATCACCCAAGACCCAACCAACCCTCTACTACCACCCCATTCCTCCCCCTCC
AGATCATCACCACCCCCAAAACTATGTCGTTTTACCCCTCTACTATCCCGCCGGCCACC
GTCGCCGTCTCCCGTGCCGCCTCATCTGCACCATCTCTTTCCTCCTCCTCGCCCTCGCC
GTTTACGTCTTCTGGCCGTCCGATCCCGACGTGAAGATCGTACGGCTCCGCCTCGACCG
CGTGCGCATCCACACAATCCCTCGCATCTCTCTCGACATCTCACTAGCCCTCACGGTCA
GGGTTCACAACGTCGACATGTACTCCATGGATTACAAGCACCTACACGTGGCGGTCGG
GTATAGGGGGAGGGAGTTGGGGCACGTGAGGTCGAGCCATGGTCACGTGAGGGCCAG
AGGGTCGTCCGACGTCGACGCTGAACTGGAGTTTAACGGAGTGGAGGTGTTGTATGAT
GTGGTTTACTTGCTTGAGGATTTGGCGAAAGGTACGGTTCCCTTCGATACTGTCACTAA
GGTTCGTGGCCAGCTTGGGCTGTTCTTCATTCGGCTTCCTCTTGAGGTAAATTTCAATTT
GCATACCAATTTCGTTTTTAATTGTTTAATTTGTTCACTCCAAGATGGAAAATGCCATTA
ATCCCTAGATTTTCTCTTAATCTTAATTATTTTTAAGAAAAAAAAAATAGAGATAAATA
ACACTGCTAGTCGAAGATCGGGCCATCAGGAATCTTGACAAAATAAATTAATCATAGGT
TCTGATTCGTTGTCGTGTGGAAATTTAGTGGCTCCATTCAATTTGATGAAGTTTTGAAG
ATACAAAACCGTAATCTTGAATGTGGGACCATTATAAAAATTTGGAACTACGGGCATAA
TTTATTTATTTTGGTACCATGCAAGTTGATGGGATCAGATGCGAATTCATGAAATGCCAC
ATTTTAAGTTTATTACTGTCATTTGTGATAGTGACTGTCATTTTCACGACTTGTCCTATTT
AGAATGATGGATTTTTACCCACCTTGAGTAGCCAGGTTGGTAATGGCGAACTTATGATT
GTCTTGATTAAACGCATTATTCTGAGCTCGATTCTGTTTAAACAACCCAAGATATAAGTG
GGGCCGTGGCGGAGGATGTTACACTGGCCTCCTGAGGGTTTAGGTTCCATGGATGGGT
TCAAAGCTACGATATGAGATGGTTTCCCCGTTATCCAAAAAATAAAAGAACGATGGATT
TGTTTGCAAAATTATTTGGAGATTATAATGAAGCAACTTACAATCAACCGGACATCCGA
CCAAATCCTGAATGCTTGAAATTTGTTATATTTGAATTTAGGTTTGAATACAACTTTAGA
TGCCTTAAATTTTTTTTTTTGGATAAAGTAATGAGTATTAATAACAAAAGGAAACTTATT
TAAGTATTAACAACTTTAGATGCCTTAAAATTACAACTATTTATTACTATTATTATTATTAT
TATTATTATTTGGCTAGATTTAAAATTACAATCACTTGAGATCCATTACCAGAATCCTTTT

ATTATCATTTCATTTACGGCTAAATCTTATATGAAATCACAGGTCGTTTTGTATTTATATAC
ACTTCAGCTCTATGTAGACTCAAATACATTGATTATTGATGATCATTTTGTCTCTCCTTGG
TCCTTGGTGGCTGGTAACTCTATTTGGGGTCAATACTTTGTGCTGGCCACGGCCACTTT
TTGTGGTGGTTGTAAGTAATTATCCTTTTTCTTTTATTCCTCAGTTATATGTAGAGTACTA
GAGTCCTATGCTTTGTCGGCTCATTTTGCCCCTCCATGTGCCATTTAGCTCTATCTAGGG
TCATATCGTTTGTCGGCATTACATATTTTCCCTCTATCTCATCTAAAATTTTTAATTTGAGG
TCAGAAACACAGAATCTGTTTTCACCATTCTGCTCTATGGCTGCATTATCTGTCATTGGT
ACACCTTGAAAATAATACAATATATAATTGGTTCCTGGTTTATAGATTGATAGATCTTTTA
CATATTTAGGATTTAGTTGGATTAGTTTGAACTTTGAAGTTATGAAGGTAAGTTGACCTT
GACATGCTAGTTGGCCTATTTATTTCATGAATATTATATGAGAGTAGACACGCATGTTGA
CCCTGAATGCTTGATATATTTGTTTGCAAAATTATCTGGAGATTATAATGAAGCAATTTAC
AATCAACCGGACATCCGACCAAACCCTGAATGCTTGATATATTTGAATTTAGGTTCAAA
TACAACTTTAGATGCCTTAAAATTACAACTATTTATTATTATTATTTGGGTAGATTTAAAA
TTACAACCACTTGAGATCCATTACCAGAATCCTTTTTCCATCATTTCATTTACGGCTAAA
TCTTGTATGAAATCACAGGTCGTTTTGTGTATATAAACATACACTTCAGCTCTATGTAGG
CTCAAATACATTGATTATTGCTCATTTTGTCCCTCCTTGCTCCTTGGTGACTTGTAGCTCT
ATTTGGGGTCATACTTTGTGCTGGCCATGGCCACTTTTTGTGATGGTTGTAAGTAGTTAT
CCTTTTTCTTTTACACACTTCGGTTATATGTAGAGTACTAGAGTCCTATGCTTTGTCCGTG
GCTTATTTTGCTCCTCCTTGTGCCATTTAGCTCTATCTAGGGTCATATCCTTTGTCAGCAT
TACATATTTTCCCTCTATCTCATCTAACTTTTTTTAATTCGAGGTCAGAAACACAGAATCT
GTTTTCACCATTCTGCTGTATGGCTGTATTATCTGTCATTGGCACACCTTGAAAATAATA
CAATACATTATTGGTTCTTGGTTTATAGATTGATAGATCTTTTATATAATTAGGATTTAGTT
AGAGTAGTTTGAACTTTGAAGTTATGAAGGTAAGTTTACCTTGACATGCTAGTTGGCCT
ATTTATTTCATGAATATTATATGAAAGTAGACATGTTGATGCTGATGACTTGTGGAATAAT
CCTTTACCTTAGCCATGGTTCGATGAAATTTTTTGTTGTATTGGCCCTTATGAAGGTAAT
AGCTTGTCCACCTATCCAAATGAAACCATCCGAATTGTAAAGCCCTAAACTCATGAAAT
CCCTCCCCTTTGTTTTTTTTTCTTCAAATGTTCATCATCCTGCACCCTTTGAGGGATTTC
CTAAGGGTAGGAGCTGGGACTGAAGGCCATGTGGTTTACCAACCTGCATTTTGAGTTT
CTCTTAGAGTTGGCTGCTCGCCCTCTGACCTTTGTGGAAATGGCTGTTCTTTAGTGAAG
CCCCATCTTGAAAGTTGCAATAAGCAATCACTTCTCACCAAATTGTCATTAAAAAGGGA
CTGAGACTTACCATCTAATTGTTATTTTTCAACATTGAAATGTAAAGCCCCCAACATTGG
AATTGTTACCATCTTTGTATATCTGGCGGTTACTTTTGTCAATATTCTGGGAGAGAATTG
CTTTGGTTTCAGAGCTGTGTAGTGTTCTCATCTTCTTGATTGTTCCACTGGAATGCTTTT
GAAAATACTCATTTCAGTCTCCCCGCCTTCTAATTTTGAGTAATTTTCAGTCTAGAGATG
TGAAAATTAGTTCTTTATCTGACTGAAAACATTATGTATTCGGAATGTTAAACCTCTGGT
CAAATTCTTGAAGCCATACACAAATCGAAATGATGAAACAGATTATTGTAACTATGTTG
CAAAATCCAATAATAAAACGATTTTCTGAGTTTGCTGCTTTTGATAATTCTGCCATTCTG
TTAGTTTGCATATCAGAAGGTAGATTTTTCTAATGCCTATAAATTTTTTTTGTTTGTTTGT
ACAGACAAAAGTATCATGTGAAGTATATGTAAATACAAACAATCAGACAATCATCCGTC
AAAATTGTTACCCTGAGGTGAGACATCAATTATTTCCTGCTGCTGTTGTGAGTGCTGAA
TTGGGTTGAACTACTGTGTATAGTTTACATTAACAATATACACATGATTCTATCCTAAAAT
CCAAAACTTGTAACTCCAAAGCTCATCAATTTAAAGTCAATCCACGCTGCCTAATACTC
AGGCCACATTCTCGAAACTGCACCATCCAAAATCTTCTCATTGCTATTCTTTTTTTGGTT
TGTTGCCCATGAATTGGCTGCTCAAAAGCAAGCACTTGTTCATGTTGCTATGAACCATG

GGGTTAATGCTGTCAAAAGGCAGTGGTCAATTGATGCCACAGCTCTTTAAAAATGGAT
CTGAAGAAGATGGTTTACATACACTCACTCTGCTTGTAGGAAACAGTGATTGATCTGTT
TTACTGACTATGTTTTTTGGTCAGTGATAAATTTGAATACTACGTGAAGCAAAGCTAGAT
GGTTCAAGGGTGCCTGTGGCGGCCACTATATATACACGTTTAAACATATTCGGGTATGTA
AAATCTGCAACAGGTAA
>LsfLEA3-2
ATGGCTAAGGTTCCCATTAACAGCTTTGTGATTCTCGGGTTGGTCCTCCTTTGTCATTCC
TTAGCTACAACTCTTCTATATATAATCAATTCCATGTTTTTTTTTTTCTTTTTTCTTCTTCT
TCTTCTTCTTACGGCTTATATATATAGCTATTGAGGGGGTTCTTTATTCTTTATTGTCTGTG
ATGCAGTACGTAGTTGTTATTAATGTCAGCTCATTTAAAATGAAATGATTTGTATTGCAG
AAGGCGATCATATGCAGTAGTAGCGGAGAATGTGAGAGTGCAATACTCAGCAACTAAT
TCAGTAATGAGGAAGGCGGCAGAGTCGGGCTGCAGTACTGTGACGACAGAAGGCGCA
CCTGAGAAGCAGAAGGAGATCTTCTGGATGAAAGATCCAAAGACTGGTAACTGGATTC
CGGAGAATCACTTTGGCGGAATTGATGTTGCAGAGCTGAGAGACAAGGTCCTTTCCAA
GAAGGACAAACTTTAA
>LsfLEA3-3
GCCTCACGCCCCACTGTCTCTCTTGCCTATATAAACCTTATTACTCGTCTTTCTCTTGCA
CATTCGAAACCCTAAAGAGAAGCCTTTGAGGAGGAAGAGAGAGAGCGTTTCGAGGTT
TGATTCGCTGCTGTGGAAGAAGAAGCTTTGCTGCAGTAGCGTCTCAAATTCTCTGTTTT
TTCAAGTTGAATCAGAAATGGCTCGCTCTCTTTCTAGCGCTAAGCTTCTCTCTGCTTTC
GTCGTCGATGAGATTTCCGTTGCTATCAACAGGTACTGCGTGGTGTATCATACGTAGAA
GTTGCTTTGTTGCTTAGAATATATGCCCTGTTTGGTTGTCGAGAAAACAGAGACCGAAA
AAGAGACTATCTTGAAATCATAAACCAACTATTTCAATTTTCTCGTCGCCCCATGTCTTG
CGGGGTATTGATACAGAGATTAATTAGTGTATAACTATATGAATTGTATGTGTTATACAGG
AGAGGATACTCGGCGGCATCACAAGGCGTTGCGTCGAACGTTGCAAGAGGAGGAGGG
GCTAGGATGGTGAAGAAGCAAGGAGAAGAAATAGCTGGGAAAACAGAGACCTCTCCG
TGGGTCCCAGACCCAGTCACTGGATACTACAGACCGGAGAATCGTGCGGAAGAGATTG
ACGTTGCTGAGCTTCGTCACACGCTCTTGAAGCATAAGATCAGAACCCCTAAAAGGCT
AAAAGAAAAAAGGAAAAAAAAAAGCACGCTTAAGAGCACGAAGAAAATTGAGTGGC
TTTGATGTCTAAAGTCGAACCCGTTTGAGCTTTCGGCGGATTCTAGAATTTGTCGGGTC
TATCTATAGATATATACGTATGCTACAATCTTCTGTAGCTCCTCCGTCCTCCGTTATGGGA
CAGCTAGGTTTTCTTTCTCTGTTATATCTAGCAATAATGACAGAATAAATTTCGAAATTC
CACTCATAATTATTGTCATTTATTATTTATTTATTTACTTACAAAGAAATTTACGTATAAGC
GTTTTATAATTTATATAGTGGCTTCATTTTAATTGTTATGTCGGCTCTATCTATAGATATATA
CGATTATACTTCAAATTTATGAAATATTTTTATTTTGAGCATCTAATATC
>LsfLEA2-41
ATGACAGACAGAGTCTACCCTTCGTCGAAACCCCAAGCCGCAAATGGCACCACCACC
ACCGCCAATCCAGCCTTCCCGGCCACCAAAGCCCAACTATACGGGGCCACTCGCCCCG
TCTACCGTCCCCAACCCCACCCCCGCCGCCAGCGGCGGAGCTGCTGCTGTTCCCTCTG
CCTTTGGCTGACCCTCCTCATCATCCTCCTCCTCCTCCTCGCCGCAATAGCCGGTGCAG
TCATATACGTTCTCTACCGTCCCCACCGCCCCGACTTCTCCGTCACCGCCCTCAGAATCT
CCCAATTCAACCTCACTTCCTCTTCTCAACTCAACGCCAAACTCAACCTCACTATCGCG
GCCCGAAACCCCAACAAGAAGCTCGTCTTCTATTACGACGCCATGTCCGTTTCTGTGTC
TTCGAACGGCGTCGACGTCAGCGATGGAACGGTTCCGGCCTTCGTGCACGGCACCAA

GAATACTACTGCGTTGAGAGTTTTGACGACGAGTGCTGCTGCTCAACAACTGGACACT
ACGTCTGCTAGTACGTTGAAGTCTGACATGAAGAAAGGTTTGCCGTTGAAGGTACGGA
TGGACACTAAGGTGAAAGTGAAGATTGGAGGGCTCAAGACCACCAAGCTTAGAATTA
GGGTTTCATGTGATGGAATTGAAGCTACTGTTCCTACCGGAAAGTCGCCCACGACGGC
ATCCACTTCGGGTTCTAAGTGCAAGGTTGATCTCCGGATCAAGATCTGGAAATGGACTT
TCTGA
>LsfLEA2-3
ATGACCAAATCCGACGGCCAGAAGCGAAAATGTTTCGCATATGTTGCCGCTTTTGTGGT
GTTTCAGACCATAATCATCGTGGTCTTTGCACTAACGGTTATGCGTATCAAGAGTCCTAA
ACTAAGGTTTGGCGCCGTTGAGGTCGAGAATTTCAGCACAGCCAACGCCAATTCAGTG
AGCATGAGATTGAATGCCCAAGTCACTGTGAAGAACACAAATTTTGGTCACTTCAAAT
ACCCAAACAGCACCGCCACCATCTTGTATGGAGGCGTGGCAGTTGGAGAGGCCTTTAT
TCCTAGTGGGCGTGCTAAGGCTCGACAGACTAAGAGATTCAACATTACGGTGGACATT
ACTTCTGCCCAGATATCAAGCAACGATGTTAATTCAGGGGTTTTGACATTGACCAGCGA
GGCCAAGTTGAATGGAAAAGTGCACTTGATGAAGGTGATCAAAAAGAAGAAATCTGC
TGAGATGAGCTGCAACATGGCAGTCAATTTGGCGACGCGTGCTGTTCAGAATCTGACG
TGCAAGTGA
>LsfLEA2-54
ATGGCCGAAGAGAGTCAAGTTTCTTCTTTAGCACCTCCCGGAAGCTATGGCAGGAGCG
ACGAAGAATTGGCGTCGCACAAACCCAACGCATCACGCCACAAAAAGAGCAATAAAT
GTTTGGTATATATCCTTGCCGGGATTGTCATCCAGAGCATAATTCTATTATCTTTTGCATT
GGTAGTTCTGCGTGTCACAACTCCTGCTGTTAAACTGAGGTGGGTCGCCGTCAAAAAC
CTCAAGTATGACAGTACCACACCGTCTTCTTCACTTAACGTGACGCTGATTGCCGAGGT
GACGGTTAAGAATAAAAATTTTGGTCGCTTCGAATTCGAGAACAGCACCGCAAGTTTC
CTATATGGGGACATGAAACTTGGTGACATGAAAATTGGCACGGGGCGTGTGAAAGCTA
GGGAGACTAATAGGGTAAATGTTACGGCGGAAGTGAGATCAAACGGGTTACCAGATAA
TATGAATCTTAGCAGTGATATTGGTTCGGGCACGTTGGAACTGAGAAGCTATGCCAAGT
TGAGTGGTAGGGTTCTTGTGATGAAGATAGTTAAGAAGCGCGAGACCTCGGAGATGAA
TTGCAGCATGACTCTTAACTTGACGAGGGGTGAAATCCAGGATTTGTTATGCAACTAA
>LsfLEA2-26
ATGGCTGAGAAGAACGAGAGGCAGGGGTATCCTCTAGCACCGGCAGCCGGTCATCTAA
GAAGTGACCAAGTATCAGCAACTTTGCAATCCAAGGAGTTCCGCCGCAAGCGAAGGA
TTAGAATCGCTGCCTATATTGTTGCCTTTGTTGTGTTCCAATCTATAATCATCGCCATCTT
TGCTGTTACGGTGATGAAATATAGAACTCCTAAATTCAGGTTGGGCAATATCACTGTGG
AAACTCTCATCTCCACCCCCGCGCCTTCGCCTTCCTTTAACACGAGGTTTGTTGCCCAA
GTTACCGTAAAGAATACCAATTTCGGCCCATACAAATTCGACAACACCACGGCCACATT
CACATATGGGGGGATGAAAGTAGGCCAGGTAGTGATTCCTAAAGGTAAGGCTGGGCTT
CGTTCAACCAAAAATATTGATGTAACAGTGGATCTCAATTCTGATGCGTTGGCGAGCAC
GTCGAGCCTTGGCAGCGATATCAGTTCAGGGATATTGAAGCTGAGCACTCAGGCCAAG
TTGAGTGGGAAAGTGGAATTGATGTTTGTAATGAAGAAGAAGAAATCTGCTCAAATGA
ACTGCAACTTGACATTTGACTTGTCAGCAAAGGCACTCCAGGCTTTAGAGTGCAACTG
A
>LsfLEA2-52
ATGGCTGCGAGGGAGCAGGTCAAACCACTCGCCCCGGCAGCCTACTGGATCAGCAGC

GATGGAGGTGAGGCCATGTCCGTGCAGATCCATAAGCTGCGCCAACGCAAATACATCA
AGTGCTGCGGCTGCATCACTGCCCTCCTCTTGATCCAAGTTGTCATCGTTCTTATCCTCA
GCTTCACCGTCTTTCACATTAAAAACCCCGACATCAAGATGAACGCCATAAATTTCCAG
CGCCTGGGCCTCCAGACCCTCAACTCCCGCAAGAACGTGACGGTGGTAGCCGATGTTT
CTATAAAGAATCCAAACGTGGCCACCTTCAGATTCACTAACACCACTACCACGGTTTAC
TACCGTAGCGTGGTGGTTGGCGAGGCGAGGCTTCCTCCAGGGCGGGCCAAGGCGAGG
CGGACACTTCGGATGAATGTTACTGTTGATATTGTTCCATCCCGGATTTTGCCCATTCCT
GGCCTGAAAAGTGATTTGGGATCGGGGGCATTGAGTATGAGCAGCTATACCAAAATTG
GTGGCCGGGTGAAAATACTCAACATCTTCAAGAAATATGTTGTGGTGGAATTGAATTGC
ACCGTCACAGTTGACCCTACTAGCCAGGCGATTAAGGATCAGGAATGCAATCGCCGTG
TTTCGCTCTAG

>LsfLEA2-10

ATGGCCGAGAAGAACCAGAAGCAATCCGCAGCCGGACACACCAGAAGTGACGAAGA
GTCAGCAACCTTGCAATCCAAGGAATTCCGCCGCAAGCGAAGGATCAGAATCGCTGCA
TATATTGTTGCGTTTGCTGTTTTCCAATCTATAATCATCGCCATCTTTGCTGTTACTGTGA
TGAAGTATAGAACTCCCAAATTCAGGCTGGGAAAGATCACTGTGGAAACTCTCGTCTC
CACCCCCGCGCCTTTGCCTTCCTTTAACACGAGGTTCGTTGCCCAAGTTACTGTAAAGA
ATACCAATTTCGGCCCATACAAATTCGACAACACCACGGCCACATTCACATATGGGGGC
ATGACAGTAGGCCAGGTTGTGATTCCTAAAGGTAAGGCTGGGCTTCGTTCCACCAAAA
AAATTGATGCAACTGTGGATCTTAATTCGGATGCGTTGGCGAGCACGTCGAGTCTTGGC
AGCGATATCAGTTCTGGGATATTGAAGCTGAGCACTCATGCCAGGTTGAGTGGGACAG
TGGAATTGATGTTCGTGATGAAGAAGAAGAAATCTGCTCAAATGAACTGCACCTTGAC
ATTTGATTTGTCAGCAAAAGCACTCCAGGCCTTGGACTGCAATTGA

>LsfLEA2-47

ATGGCCGAGAAGAACCAGAAGCAATCCGCTGCCGGACACACCAGAAGTGACGAAGA
GGCAGCAACCTTGCAATCCAAGGAATTCCGCCGCAAGCGAAGGATCAGAATCGCTGC
ATATATTGTTGCCTTTGCTGTTTTCCAATCTATAATCATCGCCATCTTTGCTGTTACTGTG
ATGAAGTATAGAACTCCCAAATTCAGGCTGGGAAAGATCACTGTGGAAACTCTCGTCT
CCACCCCCGCGCCTTCGCCTTCCTTTAACACGAGGTTCGTTGCCCAAGTTACTGTAAAG
AATACCAATTTCGGCCCATACAAATTCGACAACACCACGGCCACATTCACATATGGGGG
CATGACAGTAGGCCAGGTTGTGATTCCTAAAGGTAAGGCTGGGCTTCGTTCCACCAAA
AAAATTGATGTAACTGTGGATCTTAATTCGGATGCGTTGGCGAGCACGTCGAGCCTTGG
CAGCGATATCAGTTCTGGGATATTGAAGCTGAGCACTCAAGCCCGGTTGAGTGGGAAA
GTGGAATTGATGTTCGTGATGAAGAAGAAGAAATCTGCTCAAATGAACTGCACCTTGA
CATTTGATTTGTCAGCAAAAGCACTCCAGGCCTTGGACTGCAATTGA

>LsfLEA2-51

ATGGCCGAGAAGAACCAGAAGCAATCCGCAGCCGGACACACCAGAAGTGACGAAGA
GTCAGCAACCTTGCAATCCAAGGAATTCCGCCGCAAGCGAAGGATCAGAATCGCTGCA
TATATTGTAGCCTTTGCTGTTTTTCAATCTATAATCATCGCCATCTTTGCTGTTACTGTGAT
GAAGTATAGAACTCCCAAATTCAGGCTGGGAAAGATCACTGTGGAAACTCTCGTCTCT
TCCCCCGCGCCTTCGCCTTCCTTTAACACGAGGTTCGTTGCCCAAGTTACTGTAAAGAA
TACCAATTTCGGCCCATACAAATTCGACAACACCACGGCCACATTCACATATGGGGGCA
TTACAGTAGGCCAGGTTGTGATTCCTAAAGGTAAGGCTGGGCTTCGTTCCACCAAAAA
AATTGATGTAACAGTGGATCTCAATTCGGATGCGTTGGCGAGCACGTCGAGCCTTGGC

GGCGATATCAGTTCAGGGATATTGAAGCTGAGCACTCAGGCCAAGTTGAGTGGGAAAG
TGGAATTGATGTTCGTGATGAAGAAGAAGAAATCTGCTCAAATGAACTGCACCTTGAC
ATTTGACTTGTCAGCAAGGGCACTCCAAGCCTTGGAATGTAATTGA
>LsfLEA2-9
ATGGCTAAAGAGGACAGCAAGCGAAACACAAAATGCTTGGCATATTTTGCTGCCTTTG
TGGTGTTTCAGACCATAATCATCGTTGTCTTTGCACTAACCGTCATGCGTATCAAGAGTC
CAAAACTCAGGTTTGGCGCCGTTGCGGTTGAGAATTTCAGCACAGCCAACACCAATTC
AGTGAGCATGACATTGAATGCCCAAGTCACTGTGAAGAACACAAATTTTGGTCACTTC
AAATACCCAAACAGCACCGTCACCATCTTGTATGGAGGCATGGCAGTTGGGGAGGCCT
TTATTCCTAGTGGGCGTGCTAAGGCTCGACAGACTAAGAAATTCAACGTTACGGTGGA
CATCACTTCTTCCCAGATATCAAGCAACGACATCAATTCAGGGGTCTTGACTCTCACCA
GCGAGGCCAAGTTGAATGGAAAAGTGCATTTGATGAAGGTGATCAAGAAGAAGAAAT
CTGCTGCAATGAGCTGCACCATGGCAGTCAATTTAGCGACGCGTGCGGTTCAGAATCT
GAAGTGCAAGTGA
>LsfLEA2-29
ATGGCTGAGAAGAACCAGCAGGTGTACTCTTTAGCACCAGTAGCCGCACACCCCAGA
AGCGATGAAGAGTCAGCAACTTTGCAATCCCAAGAGCCCCGCCGCAAGCAAAGGATC
AAAATCGCTGCATATATAGTTGCCTTTGCTGTCTTCCAATCTATAATCATTGCCATCTTTG
CGATCACTGTGATGAAGTATAGAACTCCTAAATTCAGGCTCGGCAAGATCACTGTGGA
AACTCTCGACTCCACCCCTGCGCCTTCTCCTTCCTTTAACATGAGGTTCGTTGCCCAAG
TTACTGTAAAGAATACCAATTTCGGACCCTACAAATTCGACACCACCACGGCCTTATTC
ACGTATGGGGGCATGACAGTAGGCCAGGTTGTTATTCCTAAAGGTAAGGCCGGGCTTC
GTTCCACCAAAAAAATTGATGTAATAGTGGACCTCAATTCTAGTGCGTTGACAAGCACC
TCGAGCCTTGGCACCGATATCAGTTCAGGGATATTGAAGCTGAGCAGCCAAGCCAGGT
TACGTGGGAAAGTGGAATTAATGTTTGTGATGAAGAAGAAAAAATCTGCTCAAATGGA
CTGCACCTTGATATTGGACTTGTCAGCAAAGACACTCCAGACCATGGATTGCAATTGA
>LsfLEA2-43
ATGGCCAAAGAGGACAGCAAGCGAAACACAAAATGCTTGGCATATGTTGCTGCCTTTG
TGGTGTTTCAGACCATAATCATCGTTGTCTTTGCACTCACCGTTATGCGTATCAAGAGTC
CAAAACTCAGGTTTGGCGCCGTTGCGGTTGAGAATTTCAGCACAGCCAACACCAATTC
AGTGAGCATGACATTGAATGCCCAAGTCACTGTGAAGAACACAAATTTTGGTCACTTC
AAATACCCAAACAGCACCGTCACCATCTTGTATGGAGGCATGGCAGTTGGGGAGGCCT
TTATTCCTAGTGGGCGTGCTAAGGCTCGACAGACTAAGAAATTCAACGTTACGGTGGA
CATCACTTCTGCCCAGATATCAAGCAACGACATCAATTCAGGGGTCTTGACGCTCACCA
GCGAGGCCAAGTTGAATGGAAAAGTGCATTTGATGAAGGTGATCAAGAAGAAGAAAT
CTGCTGCAATGAGCTGCACCATGGCAGTCAATTTAGCGACGCGTGCTGTTCAGAATCT
GAAGTGCAAGTGA
>LsfLEA2-23
ATGTTGGAATCCGAAAAGTTCAAAAACATGCAAAACAGGAAATGTTTTGCATACATTG
TTGCTGGGGTTGTGTTCCAGACCATAATAATTCTCGTGTTTGCATTGACGGTGATGCGTA
TCAAAACTCCCAGCGTTAGGCTAAGATCCGTGACCGTCCAAAATCTCGACTTCAATGC
ATCTGCTGCTTCCCCTTACTTCAACATGACTTTGATTGCCGAAATTGCTGTGAAAAATA
AGAATTTTGGGCATTTCAGATTCGACAACAGCACCGCCAATGTCACTTATGGTGACGCG
ACGGTTGGTGATGGGGAATTACTGAAGGGGCGTGCTAGTGCAAGGAAGACAAAACGA

GTGAATGTTACCATGGATGTGAGCTCAAATGGGTTTTCGGATAGCACCAAGTTGAGCA
ATGATCTGAACTCGCGGATTTTGACGCTTAGCAGCCAAGCGAATTTAAGAGGTAAAGT
GACATTGATGAAGGTGATGAAGAAGCGAAAAACTGCAAAGATGAACTGCACAATGAG
TGTTGATCTAGATAGCAGCATAGTTCAGATCTTGGATTGCAAGTGA
>LsfLEA2-7
ATGGCCGAGAAAAACCAGCAAGTGTATCCTCTAGCACCAGCAGTCGCAAACCCCAGA
AGTGATGAAGAATCAGATTTGCAATCCAAGGAGTTCCGCCGCAAGCGAAGGATCAAA
ATCGCTGGATACATTGTTGCCTTTGCTGTGTTCCAATGTATAGTCATCGCCGTCTTTGCT
GTTACTGTGATGAAGTATAGAACTCCTAAATTCAGGCTGGGAAAGATCACTGAGGAAA
CTCTCGAGTACTCCACCTCCACGGCTTCTCCTTCCCTTAACACCAGCTTCCATGCCCAA
TTTACGGTGAAGAATACCAATTTCGGCCCCTACAAATTCGACGACACCACGGCCACATT
CACATATCAGGGCGTGAAAGTAGGCGAGGTCGTTATTCCTAAAGGTAAGGCTGGGTTT
CGCTCCACCAAAAAAATTAATGTAGTAGTGAACCTCGATTCTAGCGCGTTGGCAAGCA
CGTCTAGCCTCGGCACCGATATCAATTCGGGGGTATTGAATCTGAGCACTGACGCTAAG
TTGAGTGGGAAAGTGGAATTGATGTTTGTGATGAAGAAGAAGAAATCTGCTAAAATGA
ATTGCACCATGACAATTAACTTGCCAACGAAGGCATTCAAGGTCTTGGAGTGCAACCA
AAAACATGAAGCTGTTTGA
>LsfLEA2-16
ATGGCCAAATGCGAGGACGGAAATCGGAGAACAAAATGCTTTGCTTATGTTGCTGCCT
TTGTGGTGTTTCAGACCATAATCATCTTGGTCTTCGCACTCACTGTGATGCGTATCAAG
AGTCCTAAAGTAAGGTTTGGCGCGGGCGCGGTCGCGGTTGAAAATTTCAGCAGTAATA
ACACCAATTCAGCTGCTAGTAGCATGAGATTAACAGCACAAGTGAGTGTGAAGAACAC
AAATTTTGGTCACTTCAAATACGAAAACAGCACGGCCACCATCACGTATGGGGGCATT
GCAGTTGGGGAGGCCTTTATTCCTAAGGGGCGTGCCAGAGCTCGACAAACTAAGAGAT
TCAACATTACGGTGGACATTACTTCTGACAAGCTGTCGGGCAATCCAAACCTTGCAAG
CGACGTTAATTTGGGGGTTTTGACGTTGAGCAGCCAAGCCAAGCTGAGCGGAAAGGT
GCACTTGATGAAGGTGATCAAGAAGAAGAAATCTGCTGAAATGAGCTGCACCATGGC
AATCAATTTAGCTACGCGTGCCGTACAAAATTTGCAGTGCAAGTGA
>LsfLEA6-1
ATGTCGAGGAGCGGCGAGCAGATGGCACGAAAGGCGGAAAGTGAAGAACAGGAAGC
GACGGTTGAAGGGCTGCCTCTAGAGAGCAGTCCCTACGTAAAGTACACCGATTTGGAG
GATTACAAACGCAAAGCCTATGGCACTGAAGGACACCTCGATCCCAAGCCCGGCCGTG
GTGCAGGTGGCACTGATGCTCCTACTCTCTCCGGCAGCGGCCTCTCCGATGGACAAGT
TTCTGCCATTGATACAGCCAACCGCCAAGGCATCCCATAA
>LsfDHN-1
GCGTCATATCCGCGTTGTCCTGCTATACCAATCCAAGCTCTATAAAATCAACCGAACTCT
CTCACTAAACCATCAAATTCAAACGCCAACTCTGTCAGCTGCTAAGTGTTCACTACATA
TTTCATTGCGTTCGTTTAACACCCTTGGTTTTTCTTAGCTCTTTATATTTTGTGTTTGTAC
GTTAACGTCATCGATCAGTATGGCAGAAGAGCACCATCACCACCACCACCACCACCAC
TACCACGACGGCAAGAGCCATGAGTCTGAGACCCCCATCACAACCGAAGAAGTTGAG
ACCAAAGATCGTGGTTTGTTTGATTTCTTGGGGAAGAAAAAAGAGGAGAAGTCCCAA
GAAGAGGTGTTAGTTGCCGAGTTTGAAGAGAAAGTGCAGGTGTCTGAGGCGGAGGGA
AAGATGGAGGAGCCGCCCAAGGAAAAGGAGAAGAAGCCCAGTTTCTCGGAGAAGCT
ACCCCGATCTGGCAGTTCTAGCTCTTTATCCGTAAGTTTACTTCTTATATATATATATATAT

ATATATGTATATGTATATATGTATTGCCATGATCTATTTCATCATTTGTTATGAAGCCTAAA
AATCCTATATCGGCCGATGGTCCAGCCGATTAGAAGGCTAAAGTCGTCATTTCATACGAT
CAGCCTTGCCAAGGATTCACCCTAATAGCTGGGAAATGATCTCCATTTGCCACGTGTAA
ATATGAAGGTTCAATGACTATTTTGCCCACCCATTAATTTCTAGTTGTTGAAAGTTGTTT
GTATATTAATTTGCAGTCTAGCGATGAGGAGGAGGAGGAAGGAGAGGGGAAAGAGAA
GAAGAAGAAAAAGAAGGGATTGAAGGAGTTTCTATCAGGAGAGAAGGAAGAAGAGA
AGCACGAGGACACAACCGTCCCTGTTGAGAAATGTGAGGAACCAGTCTATGTAGCGC
CACCACCACCACTGTACTCAGAGGAGAAGGTCGAGGCAGTCTATGCAAAGCCACCAC
CACCACTGTACACAGAGGAGAAGGTCGACTATGCAAAGCCACCACCACCACTGTACTC
AGATGAGAAGGTCGGGGCAGTCAAGGCAGAGCCACCACCACCACCACCTTACTCAGA
GGAGAAGGTCGAGGCAGTCTATGCAGAGCCACCGCCTCACCCAGAGGAGAAAAAGG
GCATTCTAGAAAAGATCAAGGATAAACTACCCGGACAGCACAAAAAGACCGAAGAGG
TCCCTTCTCCCCATCCGGCTGAGTGCGCTGTAGCAGAGACCCACGAAGGTGAGGCAAA
GGAGAAGAAGGGTATTTTCGAGAAGATCAAGGAGAAGCTTCCTGGGTATCACCCCAA
GACTGAAGAAGAGAAGGAATAGGAAAAGGAAAAAGAGTGTGGTTCCCACTAAGAAG
ATTTCCATATCGTGCTTATAGAATGATGATGCTTGTTTTGTTTGTTCATGTTATTTGTTGAT
AAGGTTATCGCATTTTTTGTCTTTGTGTTTTTTTTTTAAATGAATTTTTATACTATATGCAA
GTGGTTTTAGAGCTAATTCCATGTTTTGAGAAGGGAGTTTATATATCATTCCTTTCTTTAT
TTTGTGATTTTACATATGTAGCATGTCTATTTTCTGTGTGTAAAAACTGGAAAAGAAAAC
AGCTTGTTCTGCTTTAATTTGATTTTTCACTTTTGTGTTTCTGAGTGTT
>LsfLEA2-40
ATGACAGACAGAGTATACCCACAAACCAAACCCACCACCACCACAACCAACCCACCC
CAAACCGTAAACGGCGGCGCTACCACCAACCCAACACCCTTCTCCACCACCAAACCC
CAGCTCTACAACCCCACCCGCCACCCTTACCGCCCTCAACCCCGCAACAACAACCGCC
GTTCTCGTCGGACTTTCACCTGCTGTTGCTGCTGCTTCTGGTTCATTCTCCTCGTCCTCC
TCCTCTCCCTCCTCGCCGCCACAGCCGGCGCCGCCCTCTGGGTCCTCTACCGTCCTCAC
CGCCCCGCTTTCTCCATCTCCGCCTTCAAAATCTCCCTCCTCAACCTCACCACCACTTC
AGATTCCTCCTCCTCCCATCTCACCACCACTTTTAACATCACCCTCTCCGCTCAAAATCC
CAACAAAAAACTCACATTCTTCTACGACCCATTCACCATATCCGTTCTCTCAAACGACG
TGCTCCTGGCAAACGGGTCATTCCCACTGTTCACTAGCTATGCGAAGAACACTACAATT
TTACGGACGACGCTGTCGGCGTCGCGAGATCTAGACGCGGAGTCGGTGACGGCGTTG
AGATCAGATCTGAAGAAGAAAAACGGGTTGCCGTTGAAAATGGAGGTGGATACGAAG
GTGAAGGTGAAGATGGATGGAGTGAAGACGAAGAAGGTGGGGATTAGGGTTACGTGT
GAGGGAATTCATGGGGTTGCACCGACAGGCAAGTCTCCGTCTGTTGCTTCAACTTCTG
AGTCCCAGTGTAAGGTTGATCTTCGAATCAAGATCTGGAAATGGACTTTCTAA
