# Supplementary material for: Genome-wide identification and expression analysis of late embryogenesis abundant (LEA) genes reveal their potential roles in somatic embryogenesis in hybrid sweetgum (Liquidambar styraciflua × Liquidambar formosana)
Source: For Res (Fayettev). 2023 May 29;3:12. doi: 10.48130/FR-2023-0012 (PMC11533890; doi:10.48130/FR-2023-0012)
Supplement: Supplementary file 1 — Supplementary data to this article can be found online. [file FR-2023-0012-S1.zip › 10.48130_FR-2023-0012-Suppl-TableS1.pdf]

S1:The Amino acid sequence of LsfLEA genes

>LsfLEA1-5
MHSAKEKLSDMASAAKEHVDIYKAKAKEKIAHEGRKAKEAQAKMEMHEAKARHAEEK
LNAKQSHLYGHQVHEPPLVAHIIGTMNQWWVSMDTMGRSLLELRPLYLYR
>LsfLEA1-4
MQAGKNAAASAKETAANAAASAKSGMDKTKATMQEKAERMTARDPMQKEMATEKKE
TKKTEAELNKQEAHEHNAAARQMASTGGTAGTYTHSATGATGHPTGGHQMSAMPGHGT
GQTTEGYVAEGAAGSKPTGINTGTGQTTAHNTRAGGGTNTGGFGTGDTYT
>LsfLEA1-1
MQSAKEAVSNMTASAKAGMDKTKATVQQKVEKMTAHDPIEKDMATQRKEAKITEAELN
KQETREHNAASKQAGKAGVAGGTTTTHHSTTGLGGFSTGGHPPTTHSTTGTGGYTTGGH
PTTTHSTTEAAGGYPTGGHHMSAMPGEGTWQPTWQEAEELLVPRGTGTGTGGTTGNTR
LGGGATTGNGTGDAYS
>LsfLEA1-3
MHSAKEKLSDMASAAKEHVDIYKAKAEEKVEKAAARTEEEKKIVHEGRKAKEAQAKME
LHEAKARHAAEKLNAKQSHLYGHHVHEPPLVGAHHRHNEPVVGVHGHHGAQPVGTVAP
LPLPVVGTQHGHNVNEPVVGVQGHQPVGTAAPLGGGTVPTYPLGGHPPRREYM
>LsfLEA1-2
MHSAKEKLSDMASAAKEHVDIYKAKAKEKVEKAAARTEEEKKIAHEGRKAKEAQAKM
EMHEAKARHAEEKLNAKQSHLYGHQVHEPPLVAHIIGTMNQWWVSMDTMGRSLLELRP
LYLYR
>LsfLEA2-42
MEDRRDTVIGYPVEFFRGQSNYQVSHYATTSAAAASTSASATNNNVNTARAHVVQPPTVS
HHHQQRPGKSKLSNSCLALIIASISVAVMFVIYAIVSFLNAFEFPEFTLNSVSISPFNLSVHSH
TTTTNWNVSFYIRNPNRRMSFSYDMVQASLFYKDAQFLSPGTIPPFSQGTEDSTSVQAKL
AASSVYVDDGLRKAILDCWSRRGAAVDLNVKVQALIRFDPGTNSESRGSVWVFCKDVKT
EFLSNSTLGTMVGGSMKCKTRLSLSLQPFHQLSNNHHLEQIKYQQLIESGTLRPCEMPHE
KKALISMF
>LsfLEA2-33
MAEEKPTQEEQIQQPQTNKMNPGCGCGGKTHPSHHLRRGICTCIAIFLLLAGVTALTVWL
VYRPHKPKFAVVGAAIYDLNTTSPPLISTTMQFTIVTRNPNKRVSIYYDQLSAFMSYRNQAI
TPQTMLPPLYHRKHSTVALSPMLGGGMVPVSVEVTNGLAMDEAYGVVALRLVLLGRLR
WKAGAIRTGRYGVYVKCDMLVGVKKGVTGQVPLLGAPECKVDI
>LsfLEA2-3
MTKSDGQKRKCFAYVAAFVVFQTIIIVVFALTVMRIKSPKLRFGAVEVENFSTANANSVSM
RLNAQVTVKNTNFGHFKYPNSTATILYGGVAVGEAFIPSGRAKARQTKRFNITVDITSAQIS
SNDVNSGVLTLTSEAKLNGKVHLMKVIKKKKSAEMSCNMAVNLATRAVQNLTCK
>LsfLEA2-53

MADAKQSHLNGAYYGPSIPPTKSYHRHGRGGGCGCGCCLLSFLFKLIFSAIIIVGLAVFVF
WLIFRPTPVKFHVVDASLTEFNLTNSSTLNYNFALNMTIRNPNKYIGIYYDRIEANAYYGG
QRFNTVPLTPFYQGHKNTSSLNPVFNGQSLVVLGASELSAFDAEKSAGVYNIDVKLYLRIR
FKLRKIKTGRFKPKIKCDLKVPLSSNGASAGTFEATKCEIDF
>LsfLEA2-23
MLESEKFKNMQNRKCFAYIVAGVVFQTIIILVFALTVMRIKTPSVRLRSVTVQNLDFNASA
ASPYFNMTLIAEIAVKNKNFGHFRFDNSTANVTYGDATVGDGELLKGRASARKTKRVNVT
MDVSSNGFSDSTKLSNDLNSRILTLSSQANLRGKVTLMKVMKKRKTAKMNCTMSVDLDS
SIVQILDCK
>LsfLEA2-20
MHTGDGLPVQTPPKTRPIKRHHTTRYYVHRVHESLTTRVSKILCSIFLGLLLVIGVVAFIVW
LSLRPHRPRFHIRDFSVPGLNQVNGIDNAVITFNVTARNANQNIGIYYDSMVGSVYFEDQR
IGSTPLLFPFYQEPKNTTVVYGTFSGATLTVDSQRWTDFKNALAKGSVIFRLELTSTIRFKIS
RWKSKRHHAHADCAIGVGQDGLILAVSKDKRCPTYFT
>LsfLEA2-52
MAAREQVKPLAPAAYWISSDGGEAMSVQIHKLRQRKYIKCCGCITALLLIQVVIVLILSFT
VFHIKNPDIKMNAINFQRLGLQTLNSRKNVTVVADVSIKNPNVATFRFTNTTTTVYYRSVV
VGEARLPPGRAKARRTLRMNVTVDIVPSRILPIPGLKSDLGSGALSMSSYTKIGGRVKILNI
FKKYVVVELNCTVTVDPTSQAIKDQECNRRVSL
>LsfLEA2-49
MAVTMHAKSDSEVTSLDASSPPRSPRRPLYYVQSPSQHDVEKMSYGSSPIGSPHHYHCSPI
HHSRESSTSRFSASLKNAHHHKHSAPWKKMHRVVDVDDDDEEDGDGDGGGSAHKVRLY
LCFVVGFVALFTVFSLILWGASKTYRPKIFVKNIVFRNFNIQAGMDSTGVPTDMLSLNSTV
RISYRNPATFFGVHVSSTPLELYYYQLKVASGHMKEFYQSRKSNRNVVTMVLGYQVPLYG
GVSVLGGARNHYENVAVPLNLTFVMRSRAYILGKLVKSKFYKRIRCSVTLRGNQLGKLQN
LTDSCIYQ
>LsfLEA2-18
MADRVHPADSPSSDTASSHNPDAPSKPTPPQPEKPVPPIGTYVIQIPKDQIYRVPPPENARRF
EKLTRRKTRRSPCCCCLCWLLSFIALLVVLIAISAGIFYLVVRPKSPKYSVDNVSITGFNISSS
LAISPEFDVTVRAENPNKKISIYYEKDSSVNIYYSDVKLCSGVLPVFYQPSKNVTVFQTALS
GSGIVLGSTVHETLVNAQNQGQIPFKLKLKAPVRIKVGSVKTWTITVKVSCDITVNKLTAD
AQILSKDCDFGVKLW
>LsfLEA2-44
MSSKDCGHHHHKWRKRLRRLCIGLLIFNFILLVVILLVWAILQPTKPRFILQDATVFAFNVS
APNTLTSNIQVTISSRNPNDHIGIYYDRLDVYAVYRDQQITLRTSIPPIYQGHKDVNVWSPY
VLGNSAPIAPYNGLALSQRSGEWERAGLDQDGWTGEMESWDIHIGEVSPVRQVSGVSHL
WES
>LsfLEA2-47
MAEKNQKQSAAGHTRSDEEAATLQSKEFRRKRRIRIAAYIVAFAVFQSIIIAIFAVTVMKYR
TPKFRLGKITVETLVSTPAPSPSFNTRFVAQVTVKNTNFGPYKFDNTTATFTYGGMTVGQV
VIPKGKAGLRSTKKIDVTVDLNSDALASTSSLGSDISSGILKLSTQARLSGKVELMFVMKK
KKSAQMNCTLTFDLSAKALQALDCN
>LsfLEA2-37
MAELLEKAKNFMAEKVANIKKPEASVEDVDLKDVSRECITYNAKVAVKNPYGHALPICEI

SYTFKSSGRVIASGTIPDPGSLKANDVTMLDVGLKVPHNVLVTLVKDIGADWDIDYELEV
GLTIDLPIIGNFTIPLSKKGEIKLPTLSDIF
>LsfLEA2-11
MAEKYHHLTPAAPGQATARDEESEILQSKELRRKRRIKIAAYVVAFVVFQSIIIAIFAVTVMK
YRTPQFRLGRVTVESLTSTSTPTPSFNTRFVAQFTVKNTNFGPYKYDNTTVEFSYGGMVVS
QVVIPKGKAGLRSTKKRDVVVELNSAALTSTSSLGSDINSRILTLSSQARLNGKVELMFVM
KKKKSAQMNCTMTFNLDAQSLQGLECN
>LsfLEA2-36
MEVAAASSSSSKDGKAVKRVRARRRRTICIAVIAAILGVVLLIVILGLTVFKPKRAVTTVNSF
KLDHMDVSMDVARLRVNLNITLNIDLSVKNPNKVGFKYTNSSAHLNYRGQVVGEVPIPA
GKISADSTMPMSVTLTLLADRFLSNSDIYSDVLSGTLPLSTYTKISGKVYILKVFKFHVVTE
STCDVVVNVLNRTTPTPECKYKTKL
>LsfLEA2-30
MAEKNQQVYPLALSTGGQPRSADEESATLQSKEFRRKRQIKIAAYVAAFVVFQTIIIAIFAV
TVMKYRTPKFRVGKVTVDTLVSTAAPSPSFNTRFIAQVTVKNTNFGPYKFDNTTAVFTYG
GMTVGQVVIPKGKAGLRSTKKMDVTVELNSDGLMGTSALGSDISSGVLKLSSQAKLNGK
VELMFVMKKKKSAEMNCTLTFDLSAKALQALECN
>LsfLEA2-48
MTLENHHQVSSERKQRQSTPKRRCLIAVGAVLLMLLLLLFVIALILALTVFKAREPRAQVV
SAAVEGIAPRVSLPLIRIQLNITLDLKLLVENPNHASFKHGTGKSVLLYNGHQVGEADIFPG
NIPARGSTTLACRLTLQVDELASDVTPLIRDVLAGELLMETHTGIPGRVSFLGILRKHAVAIS
DCQIAIGIPDMKIRRQDCKQRAKL
>LsfLEA2-45
MAEENQKYPLSPAAAGHPRSDEESATLQSKELRRKKRIKIAAYIVAFAVFQSIIIAIFAVTVM
KYRTPKFRLGKVTMETLTSTPTPSPSFSSRFVAQATIKNTNFGPYKFDNTTAVFTYGGMTIG
QVVIPKGKAGFRSTKKIDVTVDLNSSGLTSIDGLGSDISSGILKVNSQAKISGKVELMFVM
KKRKSAEMNCTLTFDLSAQTLQAHCN
>LsfLEA2-24
MAWTATTSTTEAQDKTVTGYPAQPSQIAAGYPPPTTFPVTTAYPYAVPPPAVFSQTYHPPSP
PRWRYYREGGRSTLLCRLVSAVFAVLFAIAIFFFIIWLILKPRLPEFQVDSASVAPLNVTKTE
LSATWDITFRVRNPNEKLSVYYHRIEAWVVYGEEDLLYGTDLPPFYQEKRNETTVEAQLA
VVSDTADGYVLRAISEEKARGLVSFDVRVLALINFRKGVWQTRRHLLRASCEGVIFGFSAP
NNGTGTLMGGPRACKVGL
>LsfLEA2-4
MCDSKNFYVWLLQVFALLGLLAFLLWLTLRPKNPTYTIVDFSVPTSNQSNNTSSENGAISY
VLEIQNPNKDSGIYYNDILLTFYYGQDSVGEKTIPSFYQGKHKTRQLSDHFDANARVWKA
LRSTILNATAELKVGLVTKIRYKMWGRKSKHHGMNLQGQIRIGSDGKISGKNKKIKLKHT
SKRWRSRYA
>LsfLEA2-50
MAEKNQQVYPLAPAARQPRSDDEETATLQSKEFRRKRQIKIAAYVAAFVVFQTIIIAIFAVT
VMKYRTPKFRVGKVTVDTLASSAAPSPSFNTRFVAQVTVKNTNFGPYKFDNTTAVFTYGG
MTVGQVVIPKGKAGLRSTKKMDVTVELNSDGLSSTSALGSDISSGVLKLSSQAKLNGKV
ELMFVMKKKKSAEMNCTLTFDLSAKALQALECN
>LsfLEA2-9

MAKEDSKRNTKCLAYFAAFVVFQTIIIVVFALTVMRIKSPKLRFGAVAVENFSTANTNSVS
MTLNAQVTVKNTNFGHFKYPNSTVTILYGGMAVGEAFIPSGRAKARQTKKFNVTVDITSS
QISSNDINSGVLTLTSEAKLNGKVHLMKVIKKKKSAAMSCTMAVNLATRAVQNLKCK
>LsfLEA2-54
MAEESQVSSLAPPGSYGRSDEELASHKPNASRHKKSNKCLVYILAGIVIQSIILLSFALVVL
RVTTPAVKLRWVAVKNLKYDSTTPSSSLNVTLIAEVTVKNKNFGRFEFENSTASFLYGDMK
LGDMKIGTGRVKARETNRVNVTAEVRSNGLPDNMNLSSDIGSGTLELRSYAKLSGRVLVM
KIVKKRETSEMNCSMTLNLTRGEIQDLLCN
>LsfLEA2-16
MAKCEDGNRRTKCFAYVAAFVVFQTIIILVFALTVMRIKSPKVRFGAGAVAVENFSSNNTNS
AASSMRLTAQVSVKNTNFGHFKYENSTATITYGGIAVGEAFIPKGRARARQTKRFNITVDIT
SDKLSGNPNLASDVNLGVLTLSSQAKLSGKVHLMKVIKKKKSAEMSCTMAINLATRAVQ
NLQCK
>LsfLEA2-1
MENPPRPVTGYPVTGYPPPQSATNPRPNGYPYNAAPPYYNPNPYIYANPRATFLRRFIAIMI
GLFIITGTIVFIVWLVLRPRIPQVTVDSVSVTNFTTSSSQVTGNWDVRFSVRNPNKKMSIYY
DSIVSSLFYEAAFLSRTTLPPFDQDTRNLTTVRAEFAAAGVYVDGRLVNEINSERARGAVG
FDVTVQARVRFSAGAWKARRRFLRVLCEDVAVGLSSNGASGTLVGGPKECRVGL
>LsfLEA2-21
MAEKEQVDRLSSYDADDKEALLHLKKIRREKCIKCCSCVAALMLVQAIVIIILAFTVFRVK
DPIIKVNGVTVTRIDLINGTIPRPGTNMSVIADVSVKNPNVASFKHGNTTSSLYYRGTLVGE
ARGPPGQSKARRTTRMNITVDIITDRLTSNPNLKSDVGSGLLTMSSFSRVGGRVKIMKIIKR
NVIVTMNCTTTVNISTQAILKQQCKRHVKL
>LsfLEA2-8
MASSPKTQPTLYYHPIPPPPDHHHPQNYVVLPLYYPAGHRRRLPCRLICTISFLLLALAVYV
FWPSDPDVKIVRLRLDRVRIHTIPRISLDISLALTVRVHNVDMYSMDYKHLHVAVGYRGRE
LGHVRSSHGHVRARGSSDVDAELEFNGVEVLYDVVYLLEDLAKGTVPFDTVTKVRGQL
GLFFIRLPLETKVSCEVYVNTNNQTIIRQNCYPEGACGGHYIYTFKHIRVCKICNR
>LsfLEA2-13
MSKAHRPPSGRTNLASCIVATIFLIFVVIIILIVFFTVFKPKDPQISVNAVQLPAFSISNNTANF
TFSQYVSVKNPNRAVFSHYSSTLQLLYSGSQVGFMFIPAGKIESGRTEYMAATFTVLSFPLS
VAPSAAEAGLGPPLSDVGNGFRLGPTLEIESRMDMAGRVRILHFFTHHVEAKAGCRVAISV
ADGSVLGFHC
>LsfLEA2-28
MEDQKTLAIGYPLQFGYPTNTNSASTTTTTTVATNNNNNAVDAHVVQLPPVVPYPEPPCR
SRNPHRYCNIGATLTMMAMLALIITFEVYHNKYSTNSPVFRLDSASVSPFNLSIDSQINGN
WNISFYVRNPNRWTSIAYDVIWALVFYQDQLLSDGTTSPFQQGRRGQTVVTASLAAKSVH
VDDLVRNFIFDDLTSTSGVLDFDLKVEALVRRHPGTLSEAKGGVRVSCNDVKIRFSFNTGT
MVGGSRKCKTYLDDDDERGG
>LsfLEA2-38
MAEKNQKYPLSPAAAGHPRSDEESATLQSKELRRKKRIKIAAYIVAFAVFQSIIIAIFAVTVM
KYRTPKFRLGKITMETLTSTPTPSPSFSSRFVAQATIKNTNFGPYKFDNTTAVFTYGGMTIG
QVVIPKGKAGFRSTKKIDVTVDLNSSGLTSKDGLGNDISSGILKVNSQAKISGKVELMFVM
KKRKSAEMNCTLTFDLSAQTLQALECN

>LsfLEA2-35
MASCNPSRRGLKICCGVTAIFLIIVVIISVTLSLTIFKPKQPQVTAHPTSLENIQFTVVPVPSLN
ITLGMMVSIENQNYGSFKYKNTTAYVNYHGALVAEVPIEHDKVPARGKVNITTSVEIMGD
KLILSPYFWDDVAAGCLNLTSMATLHGKVSVLKILKIRATAFSTCEISVFVQSQNIKSNCQS
KIKL
>LsfLEA2-22
MTSRANANGEHTARPPTNSHHQHYYPSSSSSSSASFKGCCCCLFLLFSFLALLVLAVVLVIV
LAVKPKKPQFDLQQVGVQYVGITANPSSTSTTPTTASLSLNIRMMFTAANPNKVGIKYGES
KFTVMYRGIPLGRASVPGFYQPAHSVRQVETTVAVDRVNLLQADAADLIKDASLNDRVEL
RVLGEVGAKIRVLDFTSPGVQVSVDCAVVISPRKQSLSYKQCGFDGLSV
>LsfLEA2-34
MSVKDCGHHHDERRRRNWRILACLVFLLFLILFTIFLVWIILRPAKPRFILQDATVYSFNVS
SPNFLTSNIQVTVLSRNPNNLIGVYYEKLDIYASYRNQQITLPTSLPTTYQGHKDIVVWSPF
VYGNNIPVAPYLAVALSQDQMAGMVLVNIKINGRVKWKVGTWISSRYHLYVNCPAYIAFG
NRNTGIPVGPAMKFQLAQGCSVDVGK
>LsfLEA2-10
MAEKNQKQSAAGHTRSDEESATLQSKEFRRKRRIRIAAYIVALGKITVETLVSTPAPLPSFN
TRFVAQVTVKNTNFGPYKFDNTTATFTYGGMTVGQVVIPKGKAGLRSTKKIDATVDLNSD
ALASTSSLGSDISSGILKLSTHARLSGTVELMFVMKKKKSAQMNCTLTFDLSAKALQALD
CN
>LsfLEA2-26
MAEKNERQGYPLAPAAGHLRSDQVSATLQSKEFRRKRRIRIAAYIVAFVVFQSIIIAIFAVTV
MKYRTPKFRLGNITVETLISTPAPSPSFNTRFVAQVTVKNTNFGPYKFDNTTATFTYGGMK
VGQVVIPKGKAGLRSTKNIDVTVDLNSDALASTSSLGSDISSGILKLSTQAKLSGKVELMF
VMKKKKSAQMNCNLTFDLSAKALQALECN
>LsfLEA2-43
MAKEDSKRNTKCLAYVAAFVVFQTIIIVVFALTVMRIKSPKLRFGAVAVENFSTANTNSVS
MTLNAQVTVKNTNFGHFKYPNSTVTILYGGMAVGEAFIPSGRAKARQTKKFNVTVDITSA
QISSNDINSGVLTLTSEAKLNGKVHLMKVIKKKKSAAMSCTMAVNLATRAVQNLKCK
>LsfLEA2-5
MVEKDQQAYPLSPVAAGHPRSDEESATFQSKELRRKKRIKIAAYVVAFAVFQSIIIAIFAVTV
MKYRTPKFRLGKITMETLTSNPTPSPSFSSRFVAQATIKNTNFGPYKFDNTTAVFTYGGMTI
GRVVIPKGKVGFRSTKKIDVTVDLNSSGLTSTDGLGSDISSGILKVNSQAKISGKVELMFV
MKKRKSAEMNCTLTFDLSAQTLQALECN
>LsfLEA2-56
MADNQRIHPVVHDPEAPHTPTAPLVPRGSSKSEKGAPADQYHQVPLRRTIPVMHSKPPKK
RSCFCKCLCWTVCLLLLQVIIVGIVGGILFLVFRPKLPKYSIDSLRITQFNLGNDNSLNATLD
VAITARNPNKKIGIYYESGSQITVWYTGTQLCQGSLPKFYQGHRNTTILNVPLTGQTQNAT
GLLSTLQAQQQTGSIPLNLRVNVPVRIKLGKLKLMKVKFRVRCRLVVDSLAADNAISIKSS
SCKFRLRL
>LsfLEA2-12
MADSAIRKTEDSPPSSKPSSNPTSKPVRHVVFSEIPRRPHKPDKERRRGRCCSGCFVVCCA
WGCMIGFALVLVLLVVAAIFVAFLHSEMPEVHVARVSIPSLDVVTASSQQALLSADVDIVV
EVSNKNDRAKLSYGTMTVDVSSEKISLGQTKVVAFSQKPKNSTELKIHTKVTRLAVDKED

GAFLKSNVKDGEMVFDVVLSGTIGVGVGRFTINGLPVTMACHGIQQSQVDIGQEPKCSV
KMFSFRLYTFLFILIIHYL
>LsfLEA2-19
MEGQKPPVIGYPLQFSHGQSPNPLSDNPTAQPPHSTTTTTTAAAHVVQLPPIVGHQDQPRP
RRRRSQICSGTNCGTLAAILALLAFAAIGAFLGNKYSIKSPVFRLNSASLSSFNLFVDSQITA
NWNVTFYVRNPNRWASIAYDVTQASVFYEDQLLSAGTISPFQQGRREQTLVTTASLDAKS
VHVDDWVKTFILDNLTNAGGVLDFNVKVETLVRLHPGTLSEARGRVTVSCNVKIQFSPDT
TSGTMVDGPRECKTHLDDHGHVF
>LsfLEA2-57
MGRIKFSRRGLKICCGVTAILFIIILVVVIVLSLTIFKPKQPKITTQSVTLEHAKLVIFPVFKLN
VTLGIALTIDNRNYGGFKYENSTSYVSYRGTVVAEAPIEHDRIPARGKLNVSTSVEIEGDKL
TLDPQFKQDCDSGIVNFTSSTILHGKVILLNFWKKKATTYSACNISVYVIAQAADSVCHSR
VKF
>LsfLEA2-55
MASSDNPDIVERGDKEKDHKEDDKDEGKGGFIEKVKDFIQDVGEKIEEAIGFGKPTADVA
AIHIPSINLEKAEIVVDVLVKNPNPIPIPLIDINYLIESDGRKLVSGLIPDTGTIHAHGSETVKIP
LTVIYDDIKSTYDDIKPGSIIPYKIKVELIVDVPVFGRLTLPLEKTGEIPIPYKPDIDIEKIQFKR
FSFEETVAVLHLKLENKNDFDLGINPAELSESTKLDKNGIGHINIPITFKPKDFGSALWDMIR
GKGTGYTMKGNIDVDTPFGAMKLPISKEGGTTRLKKNKGEGDDDDNVDVDDDEHALGA
VE
>LsfLEA2-51
MAEKNQKQSAAGHTRSDEESATLQSKEFRRKRRIRIAAYIVAFAVFQSIIIAIFAVTVMKYRT
PKFRLGKITVETLVSSPAPSPSFNTRFVAQVTVKNTNFGPYKFDNTTATFTYGGITVGQVVI
PKGKAGLRSTKKIDVTVDLNSDASGILKLSTQAKLSGKVELMFVMKKKKSAQMNCTLTF
DLSARALQALECN
>LsfLEA2-27
MAEKNQQVYPLALSTGGQPRSADEESATLHSKEFRRKRQIKIAAYVAAFVVFQTIIIAIFAL
TVMKYRTPKFRVGKVTVDTLVSTAAPSPSFNTRFVAQVTVKNTNFGPYRFDNTTAVFTYG
GMTVGQVVIPKGKAGLRSTKKMDVTVELNSDSLTSMSSLGNDISSGVLKLSSQAKLNGK
VELMFVMKKKKSAEMNCTLTFDLSAKALQALECN
>LsfLEA2-39
MADHQKIHPVVDVEAPPTTPLVPHGSFRSENGDPVQQNPPKHATVIPVIHSIPPKTERSRCC
CKCICWTISLLVIILIIIGATAGILYVIFRPKLPQYSINNLKISDFRLNYDMTLYAKFDVKITAQ
NPNKKIGIYYEKGGRLSVWYTGTRLCEGSLPKFYQGHHNTTVLDVSLTGQTQYGSTLMT
ALQQQQQTGRIPLDLKVDVPVAIKLGNLKLKKVRFLVGCKLAVDSLTANNKISITTSDCRF
RLKL
>LsfLEA2-2
MADQTRHNKIIKCSIIVATIVVLIAVYLITVKTIQPRPPRFRVTSVLVSPLNQLNVTTLNNNST
SQQITTAWRIDFLITNPHKWESISYEQVEASVYYKNNFLSSTIIPPFNQDRKEELGRRVTVA
ASSLEVNNGVMNAIAADMSHGAVGFNVMVVARVRVDRRYTWIQKMRGMRVTCEDVKV
GLWSHTTAGTMLGGSTPCNVHL
>LsfLEA2-40
MTDRVYPQTKPTTTTTNPPQTVNGGATTNPTPFSTTKPQLYNPTRHPYRPQPRNNNRRSRR
TFTCCCCCFWFILLVLLLSLLAATAGAALWVLYRPHRPAFSISAFKISLLNLTTTSDSSSHLT

TTFNITLSAQNPNKKLTFFYDPFTISVLSNDVLLANGSFPLFTSYAKNTTILRTTLSASRDLD
AESVTALRSDLKKKNGLPLKMEVDTKVKVKMDGVKTKKVGIRVTCEGIHGVAPTGKSPS
VASTSESQCKVDLRIKIWKWTF
>LsfLEA2-14
MAEKNQQVYPLALSTGGQPRSADEESATLHSKEFRRKRQIKIAAYVAAFVVFQTIIIAIFAL
TVMKYRTPKFRVGKVTMDTLVSTAAPSPSFNTRFVAQVTVKNTNFGPYKFDNTTAVFTYG
GMTVGQVAIPKGKAGLRSTKKMEVTVELNSDSLTSMSSLGNDISSGVLKLSSQAKLNGKV
ELMFVMKKKKSAEMNCTLTFDLSAKALQALDCN
>LsfLEA2-6
MAEKNQNNALAPERPRSDEESAALQSKELRRKKRIKIALYIVAFAVFQSIIIAIFAVTVMKYR
TPKFRLGKITVETLTTTNTSSPSFSTRFVAQATIKNTNFGPYKFDNTTAVFTYGGVKIGQVTI
PKSKAGFRSTKKIDVTVDLNSSGLTSTEGLASDINNGILKVNSHAKISGKVELMFVMKKR
KSAEMNCTLTFDLSAQTLQALECN
>LsfLEA2-7
MAEKNQQVYPLAPAVANPRSDEESDLQSKEFRRKRRIKIAGYIVAFAVFQCIVIAVFAVTVM
KYRTPKFRLGKITEETLEYSTSTASPSLNTSFHAQFTVKNTNFGPYKFDDTTATFTYQGVK
VGEVVIPKGKAGFRSTKKINVVVNLDSSALASTSSLGTDINSGVLNLSTDAKLSGKVELM
FVMKKKKSAKMNCTMTINLPTKAFKVLECNQKHEAV
>LsfLEA2-32
MADSAIRKTEDSPPSSKPSPNPTSKPVRHVVFSEIPCRPHKPDKERRRGRCCSGCFVVCCA
WGCMIGFALVLVLLVVAAIFIAFLHSEMPEVRVGRVSIPSLDVVTASSQQALLSADVDIVVE
VSNKNDRAKLSYGTMTVDVSSEKIRLGQTKVVAFSQKPKNSTELKIHTKVTRSAVDKEDG
AFLKSNVKNGEMVFDVVLSGTIGVGVGRFTINGLPVTMACHGIQQSQVDIGQEPKCSVK
MFSFRLHTFLFILIINY
>LsfLEA2-15
MANPAAPPSQTPLPPRRSKLVRCIALILLALIVLVGLVVLIIWLVVKPKRIVYTVEAASLSGF
NLTHDHLSANFDFMLRSVNPNHKVSIYYDSMEVMVSYYDNILAFAEVQPFYQPRQNVTQ
LEVKPVAKSAALMESASRDLRLEKSSGGVELDIRVKARIRLKMGAWKSRRRTLKVFCPHV
LVHFSSAKKFQRARCDVDL
>LsfLEA2-46
MGRGKCLAYVAAFVVFQTIIILVFALTVMRIKNPKVRLGVVAVEDFSTTNSTSINMRLVAQV
TVKNTNFGHFKYENSTATISYGGIAVGEAFIPKGRARARQTKKFNVTIDVTTDRLSSSSSNL
ANDINSGVLAMSGEAKLSGKVHLMKVIKKKKSGEMNCTMAVNLATRAVQDLKCD
>LsfLEA2-29
MAEKNQQVYSLAPVAAHPRSDEESATLQSQEPRRKQRIKIAAYIVAFAVFQSIIIAIFAITVM
KYRTPKFRLGKITVETLDSTPAPSPSFNMRFVAQVTVKNTNFGPYKFDTTTALFTYGGMTV
GQVVIPKGKAGLRSTKKIDVIVDLNSSALTSTSSLGTDISSGILKLSSQARLRGKVELMFVM
KKKKSAQMDCTLILDLSAKTLQTMDCN
>LsfLEA2-17
MAALLDKAKNFMVEKVAHVKKPEASVEDVDLKDVSRECIDYNAKVAVKNPYGHTLAIC
EITYTFKSAGRVIASGKVPDPGSLKANDITMLDVGLKVPHNVLVTLVKDIGADWDIDYEL
EVGLTIDLPIIGNFTIPISKKGQMKLPTLTDIF
>LsfLEA2-25
MHAKSDSEVTSLDASSPPRSPRRPLYYVQSPSQHDVEKMSYGSSPIGSPHHYHCSPIHHSR

ESSTSRFSASLKNAHHHKHSAPWKKMHRVVDVDDDDDDEEDGDGDGGGSAHKVRLYL
CFVVGFVALFTVFSLILWGASKTYRPKIFVKNIVFKNFNIQAGMDSTGVPTDMLSLNSTVR
ISYRNPATFFGVHVSSTPLELYYYQLKVASGHMREFYQSRKSNQNVVTMVLGYQVPLYGG
VSVLGGARNHYENVAVPLNLTFVMRSRAYILGKLVKSKFYKRIRCSVTLRGNQLGKLQNL
TDSCIYQ
>LsfLEA2-31
MAEPVKPVLQKPPGYRDPNVPVQPAPRPPVRKPALPPSFQPKKKRRSCCRIFCCSLCILTFIL
LLILAVSIGIFYIWFDPKLPVFHLQSFKVERFNVSVTSDGTFLDSRTVVRVEVKNPNSKLTV
YYGGTRVKVTVGEDDTELGTASLKGFTQGKKNTTSLKFTTRVKSEWVDDSVGKKLKAR
YRSRALVVFAEVRTRVGLGVGRWRMGTVGVNVLCGDMTLKKLAAGAMPKCTINLLKWI
NIH
>LsfLEA2-41
MTDRVYPSSKPQAANGTTTTANPAFPATKAQLYGATRPVYRPQPHPRRQRRSCCCSLCLW
LTLLIILLLLLAAIAGAVIYVLYRPHRPDFSVTALRISQFNLTSSSQLNAKLNLTIAARNPNKK
LVFYYDAMSVSVSSNGVDVSDGTVPAFVHGTKNTTALRVLTTSAAAQQLDTTSASTLKSD
MKKGLPLKVRMDTKVKVKIGGLKTTKLRIRVSCDGIEATVPTGKSPTTASTSGSKCKVDL
RIKIWKWTF
>LsfLEA3-3
MARSLSSAKLLSAFVVDEISVAINRRGYSAASQGVASNVARGGGARMVKKQGEEIAGKTE
TSPWVPDPVTGYYRPENRAEEIDVAELRHTLLKHKIRTPKRLKEKRKKKSTLKSTKKIEWL
>LsfLEA3-2
MAKVPINSFVILGRRSYAVVAENVRVQYSATNSVMRKAAESGCSTVTTEGAPEKQKEIFW
MKDPKTGNWIPENHFGGIDVAELRDKVLSKKDKL
>LsfLEA3-1
MARSLSNAKFLAASVVDSLSLAICRRGYAAASQGMVSASVSKGGSRTGIVGRVEERAVIK
EDSSWGPDPVTGYYRPVNRGAEIDVAELRDTLLNNKVRRAH
>LsfLEA4-1
MAELSDIKREDEEAIKWSLKAKNNKQEREDVDVASDKAREAKEGAEERSQEEKGKVGEY
KDYTAEKAKETKDSALEKAREYKDSAAEKTRETKDYTMGKVGEYKDSASEKAKEMKDS
ALEKAREYKDSAAEKARETKDSTMGKVGEYKDSAAEKARETKDSTVGKVGEYKDSAAE
KAREMKDSALGKAESTRIVQKESEARKDSNHGEDGRV
>LsfLEA4-2
MAPGMKGRERLKKGQRKDLRREGKVGEYKDYAAEKAKETKDSALEKAREYKDSAAEK
TRETKDSTMEKVGVYKDSAVEKAKETKDSALEKAREYKDSTAEKARETKDSTMGKVGE
YKDSAEKAERRRTQRLGRQESTRIVPQRKRGKDSIMRRRWESIRIIVRESERGKEATLGKF
SELKEICGDVARRAMEFLSRKKEEQRRRRRDRGGDQGEAERDGRGSQTEDGRDEEMKLE
GQEYKDEAGQKAQEPEMTLKRKGKLTHPTDVVEETRARREHGGTGGKTPELGVAPTLK
ASDQMTDQTFNDVGRLGDEGVARDDRQSKM
>LsfLEA5-3
MASNQKQREELDARARQGETVVPGGTGGKSLEAQEHLAEGRSRGGQTRREQLGTGGYQ
EMGSKGGQTRKEQLGTEGYQEMGRKGGLSTTDQSGGERAAELGVDIDESKFKTSSG
>LsfLEA5-1
MASGQRQQKEELEARARQGETVVPGGTGGGSLEAQEHLAEGRSKGGQTRKEQLGSEGY
QEMGRKGGLSTTDQSGGERAAQEGIEIDESKFRKSG

>LsfLEA5-2
MASNQKQREELDARARQGETVVPGGTGGKSLEAQEHLAEGRSRGGQTRREQLGTEGYQ
EMGSKGGQTRKEQLGTEGYQEMGRKGGLSTTDQSGGERAAELGVDIDESKFKTSSG
>LsfLEA6-2
MEKSGEQMGRKGESEQKGKLDGLPLETSPYTQNSDLEDYKRQGYGTEGHLQPKTGRGA
GFTDAPTLSGGAVSSDVKASAVDAINRKGVP
>LsfLEA6-1
MSRSGEQMARKAESEEQEATVEGLPLESSPYVKYTDLEDYKRKAYGTEGHLDPKPGRGA
GGTDAPTLSGSGLSDGQVSAIDTANRQGIP
>LsfSMP-1
MSQEQPQRPRADREPVKYGDVFPLSGDLASKPVAPQNAAMMQTAETIVWGQTQKGGPA
AAMQSAATLNERAGLVDHRDLTGITGDQGVTVAETNIPGSRIVTESIAGQVVGQYVEPSP
MVQTTPGQVLLQSAITIGEALEATARTVGDKPVDQSDAAAIQAAEVRATGSTVINPGGLA
ASAQSAASFNAAVDQDEDRIKLKDILADATGKLPADKAATREDAEGVMSAEMRHNPNLA
THPGGVAASVAAAARLNEQLDI
>LsfDHN-1
MAEEHHHHHHHHHYHDGKSHESETPITTEEVETKDRGLFDFLGKKKEEKSQEEVLVAEFE
EKVQVSEAEGKMEEPPKEKEKKPSFSEKLPRSGSSSSLSSSDEEEEEGEGKEKKKKKKGLK
EFLSGEKEEEKHEDTTVPVEKCEEPVYVAPPPPLYSEEKVEAVYAKPPPPLYTEEKVDYAKP
PPPLYSDEKVGAVKAEPPPPPPYSEEKVEAVYAEPPPHPEEKKGILEKIKDKLPGQHKKTEE
VPSPHPAECAVAETHEGEAKEKKGIFEKIKEKLPGYHPKTEEEKE
>LsfDHN-4
MADQHFQCHEKEPRDRGMFDFLGKKEEKPQTEMSMTDCNMACLSEPQEKKEVEKPTLV
EELRRSHSSSSSSSDEEGGEKKKKKKGLKEKIKEKMACQGEEEVTEISVDKCDNIVDAETT
HPEGKKGILEKIKEKLPGQHKKGEEGATTIPPKCAPDGHSTEGEAKEKKGILEKIKEKLPG
YHKNAEEEKEEEN
>LsfDHN-2
MADLRDEYGSPIQLTDERGNPVQLTDEYGNPMHLSGVATTHGTTATTGMHGGAPEIPIGLE
TVKHGGAPEAGAGHGGGQRELQQVQRSSSSSSSSSEDDGQGGRRKKKKGLKEKIKEKLT
GGKSKDEREQTTGFTASTTTTTTSTTTPGLHHQEHEKKSIVEKIKEKLPGHHKH
>LsfDHN-6
MSYQDQYGNPARQTDEFGNPIRQTDQYGNPIHHTGTTGEYGATHPSGTTGGVLGSGLGG
GQHQQHHQQVQEHQGLSGTLHRSGSSSSSEDDGQGGRRKKKGLKEKIKGRTPGGGHKD
EYSSATTNTTTPGVYGAGEQHEKKGVMEKIKEKLPGHR
>LsfDHN-5
MADQHFQCHEKEPRDRGMFDFLGKKEEKPQTEMSMTDCNLACLSEPQEKKEVEKPTLVE
ELRRSHSSSSSSSDEGGGEKKKKKGLKEKIKEKMARQGEEEVTEIPVDKCDNIVDAETTHP
EEKKGILEKIKEKLPGQHKKGEEGATTIPPECAPVGHPSEAEEKKGILEKIKEKLPGYHKNA
EEEKEEN
>LsfDHN-3
MANLRDEYGNPIQLTDERGNPVQLTDEYGNPMHLSGVATTHGTTATTGMHGGARETRTG
LETVKHGGAPEAGAGHGGGQREQQRVQRSSSSSSSSSEDDGQGGRRKKKKGLKEKIKEK
LSGGKSKDEREQTTGVTASTTTTTTSTTTPGLHHQEHEKKSIVEKTKEKLPGRHNH
